# Tuning the Transparency and Exciton Transition of D‐π‐A‐π‐D Type Small Molecules

**DOI:** 10.1002/chem.202500657

**Published:** 2025-07-22

**Authors:** Ecem Aydan Alkan, Houssam Metni, Patrick Reiser, Christian Kupfer, Juan S. Rocha‐Ortiz, Anastasia Barabash, Miroslaw Batentschuk, Jens A. Hauch, Pascal Friederich, Christoph J. Brabec

**Affiliations:** ^1^ Department of Materials Science and Engineering, Institute of Materials for Electronics and Energy Technology (i‐MEET) Friedrich‐Alexander‐Universität Erlangen‐Nürnberg Martensstraße 7 91058 Erlangen Germany; ^2^ Helmholtz‐Institute Erlangen−Nürnberg (HI‐ERN) Forschungszentrum Jülich GmbH Immerwahrstr. 2 91058 Erlangen Germany; ^3^ Karlsruhe Institute of Technology (KIT) Institute of Nanotechnology Kaiserstr. 12 76131 Karlsruhe Germany; ^4^ Karlsruhe Institute of Technology (KIT) Institute of Theoretical Informatics Kaiserstr. 12 76131 Karlsruhe Germany

**Keywords:** microwave‐assisted synthesis, optical properties, organic small molecules, TDDFT calculations, transparency

## Abstract

Organic small molecules possess significant potential for semitransparent optoelectronic applications due to their tunable optical properties and inherent transparency. However, tailoring these materials is challenging as their optoelectronic properties are sensitive to subtle structural changes, compounded by the existence of over a million potential structural designs. To address these complexities, we present a material discovery workflow that combines literature‐based molecule preselection with TDDFT calculations, creating customized small molecule structures with adjustable transparency windows. We identified fifty‐four small molecules with a D‐π‐A‐π‐D architecture, incorporating nine central (A) and six end (D) units connected by a thiophene π‐bridge. Through TDDFT calculations, we determined the theoretical absorption spectra and energy levels of the identified molecules. Ultimately, we synthesized twenty‐four molecules that exhibit promising transparency properties by selectively absorbing photons in the ultraviolet (UV) and near‐infrared (NIR) regions, with a significant optical transmission band relevant to the visible spectrum, which we will refer to as “optical window”. Characterization of the resultant small molecules revealed that six of them, in particular, exhibited selective absorption with the broadest “optical window”. We believe that our study will provide valuable insights to establish an effective material discovery workflow for highly transparent conjugated organic small molecules.

## Graphical abstract

Literature‐based molecule preselection has been combined with TDDFT calculations to enhance the discovery of transparent organic small molecules. 54 candidates featuring a D‐π‐A‐π‐D architecture were identified, and 24 molecules were selected for synthesis based on their favorable transparency. Among them, 6 molecules exhibit broad optical windows with selective absorption in the UV and NIR regions.

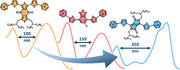

## 1 Introduction

Demand for the design and discovery of new conjugated organic materials is growing due to the diverse possible applications expected from optoelectronic devices such as organic photovoltaics^[^ ^1^, ^2^ ^]^, electrochromic devices^[^ ^3^, ^4^ ^],^ organic light‐emitting diodes^[^ ^5^, ^6^ ^],^ and organic field‐effect transistors.^[^ ^7^, ^8^ ^]^ Transparency is a highly desired aspect, providing unique application opportunities for these devices, including building‐integrated photovoltaics,^[^ ^9^ ^]^ agricultural photovoltaics,^[^ ^10^ ^]^ augmented reality devices,^[^ ^11^ ^]^ head‐up displays,^[^ ^12^ ^]^ smart windows,^[^ ^13^ ^]^ wearable displays,^[^ ^14^ ^]^ and photodetectors.^[^ ^15^, ^16^ ^]^ An ideal semi‐transparent material should selectively absorb photons in the ultraviolet (UV) and/or near‐infrared (NIR) regions while allowing the majority of visible light to pass through to maintain high transparency.^[^ ^17^ ^]^ This approach requires fine‐tuning the absorption spectrum and excitonic transitions of the resulting material through band gap engineering to create an “optical window”. Although organic semiconductors possess the advantageous ability to exhibit tunable optical and electronic properties through structural modifications^[^ ^18^, ^19^ ^],^ enhancing the absorption properties of these materials remains challenging. This is particularly relevant when improving molecular design from a vast array of available structures in a library.^[^ ^20^ ^]^ To address this issue, it is crucial to establish a workflow that predicts effective design principles for optimized molecular structures exhibiting improved transparency.

**Figure 1:**
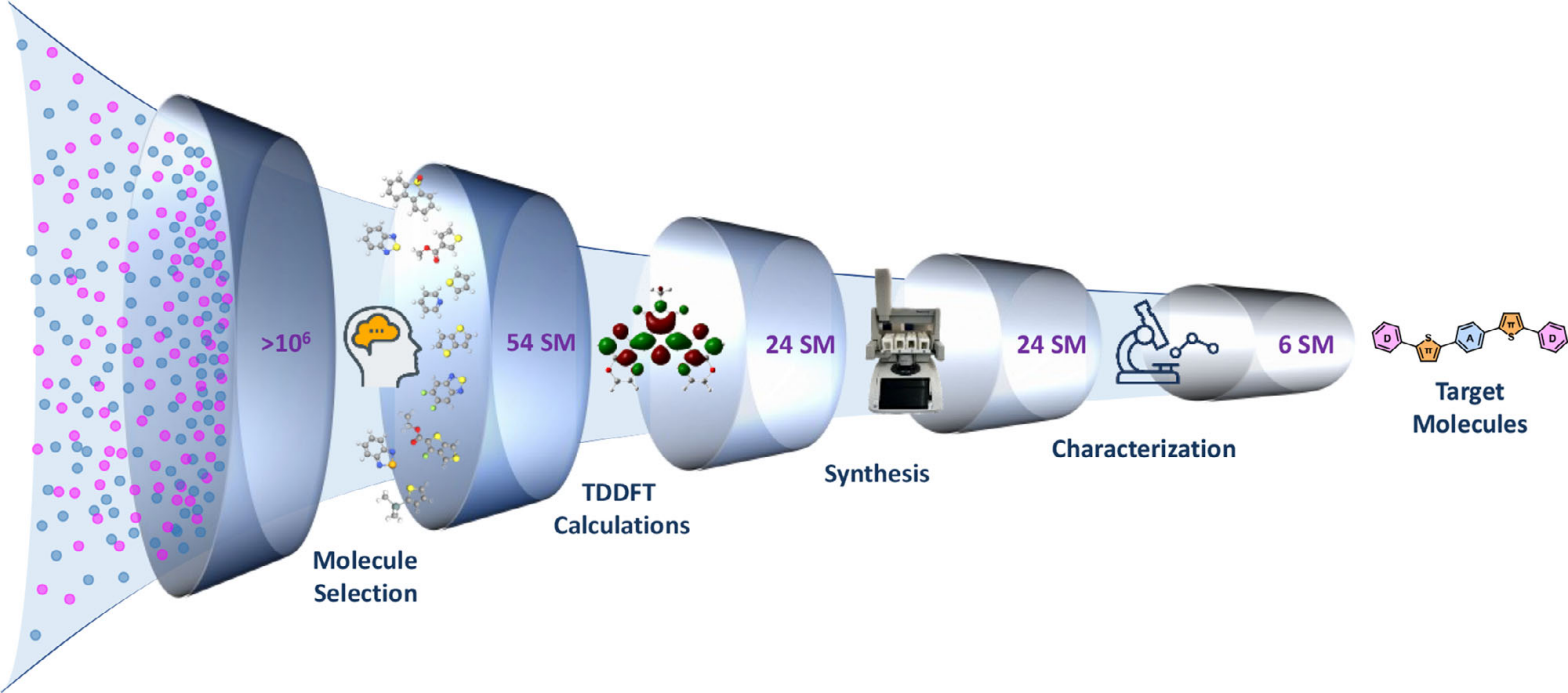
Workflow of creating semi‐transparent D‐π‐A‐π‐D type small molecules consisting of literature‐based molecule selection, TDDFT calculations, synthesis, and characterization.

**Figure 2:**
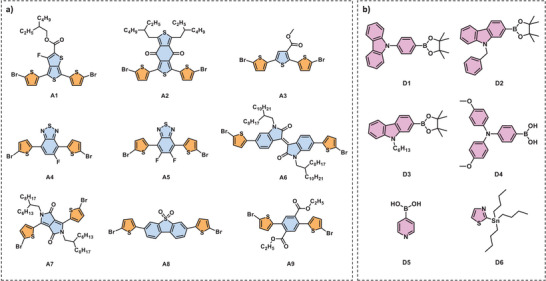
Chemical structures of a) the central unit consisting of π‐A‐π building blocks b) end units as D building blocks.

Organic small molecules, in particular, offer advantages such as well‐defined structure and molecular weight, ease of purification, and minimal batch‐to‐batch variations, which enhance the repeatability of synthesis compared to their polymeric counterparts. Moreover, their well‐defined structures facilitate the determination of the optical and electronic effects of different structural features more easily.^[^ ^21^, ^22^ ^]^ The donor‐acceptor (D‐A) approach is a widely used method for controlling the HOMO and LUMO energy levels of conjugated organic materials by incorporating various electron‐donating and withdrawing moieties and narrowing the energy gap through intramolecular charge transfer (ICT).^[^ ^23^, ^24^ ^]^ Numerous studies have shown that incorporating conjugated π‐linkers between donor and acceptor units enhances the ICT by facilitating electron polarization and charge transfer.^[^ ^25^, ^26^ ^]^ Consequently, the D‐π‐A‐π‐D architecture enables precise control over the absorption properties and charge separation of organic small molecules due to its extended conjugation and the strategic arrangement of donor and acceptor units. The thiophene unit is widely used as a π‐bridge in such materials due to its unique electronic and structural properties. It has been shown to induce intermolecular interactions, leading to effective molecular packing, which in turn enhances high stability and high charge carrier mobility.^[^ ^27^, ^28^ ^]^ However, more than a million D‑π‑A‑π‑D small molecules can be synthesized by combining commercially available dibromoarenes with arene boron or stannane moieties, using thiophene as the π‑bridge. Therefore, in this study, we intend to combine literature‐based preselection of the molecules with time‐dependent density‐functional theory (TDDFT) to generate and downsize a pool of materials to be synthesized and characterized (Figure 1). In recent studies, 3‐fluorothieno [3,4‐b] thiophene‐2‐carboxylate (FTT),^[^ ^29^ ^]^ benzo [1,2‐c:4,5‐c']dithiophene‐4,8‐dione (BDD),^[^ ^30^ ^]^ 5‐fluorobenzo[c][1,2,5]thiadiazole (FBT),^[^ ^31^ ^]^ 5,6‐difluorobenzo[c][1,2,5]thiadiazole (DFBT),^[^ ^32^ ^]^ isoindigo (IID)^[^ ^33^ ^]^ and 2,5‐dihydropyrrolo[3,4‐c]pyrrole‐1,4‐dione (DPP)^[^ ^34^ ^]^ units are commonly used as acceptor moieties for the synthesis of narrow band‐gap organic semiconductor due to their highly electron deficient nature (Figure 2a). Their highly electron‐withdrawing substitutions can result in molecules with a narrow energy gap and redshifted absorption, along with a wide “optical window”. In contrast to these moieties, units such as methylthiophene‐3‐ carboxylate (3MT),^[^ ^35^ ^]^ dibenzo[b,d]thiophene 5,5‐dioxide (DBTSO)^[^ ^36^ ^]^ and diethyl 2,5‐terephthalate (DET)^[^ ^37^ ^]^ are the frequently used less electron‐poor acceptor moieties resulting in wide band‐gap conjugated small molecules or polymers (Figure 2a). Incorporating these units can shift the absorption to the UV region while transmitting photons in the visible part of the spectrum. In addition to that, carbazole derivatives^[^ ^38^ ^]^ and triphenyl amine (TPA)^[^ ^39^ ^]^ units are promising electron‐donor moieties due to their high thermal and photochemical stability and strong electron‐donating nature, which are used widely in literature. Pyridine^[^ ^40^ ^]^ and thiazine^[^ ^41^ ^]^ derivatives were used as end units to elucidate the effect of end‐group charge transfer characteristics on the studied material set (Figure 2b). It has been proven that they significantly enhance intermolecular interactions through hydrogen bonding and dipole–dipole interactions due to their polar nature and the presence of heteroatoms.

Considering the design rules mentioned above, a subset of fifty‐four small molecules with D‐π‐A‐π‐D architecture incorporating the mentioned commercially available dibromoarenes and arene boron or stannane moieties with thiophene π‐spacers was generated. While the literature thoroughly investigates structural modifications like the donor‐acceptor approach, planarity, and substitution effects, few studies focus on how symmetrical features of organic molecules affect their optical and electronic properties.^[^ ^19^, ^22^ ^]^ Therefore, building blocks with various degrees of electron‐donating and electron‐withdrawing effects, planarity, and symmetrical features were selected for this study. Theoretical calculations were performed to pre‐select the molecules that exhibit desired properties. Selected molecules were subsequently synthesized and characterized to determine design principles for achieving high transparency in conjugated organic small molecules.

## 2 Results and Discussion

More than a million D–π–A–π–D small molecule structures can be synthesized by combining commercially available electron‐acceptor and electron‐donor moieties, specifically 1132 dibromoarene acceptor (A) units and 954 arene‐based donor (D) units (boronic acids, esters, MIDA boronates, or stannanes) from commercial supplier libraries, using thiophene as the π‐bridge. Based on literature precedents, a subset of fifty‐four small molecules with D‐π‐A‐π‐D architecture, combining nine central (A) and six end (D) units spaced by a π‐bridge, was created. (Figure S1) Central units with varying functional groups, symmetrical features, such as FTT (A1), BDD (A2), 3MT (A3), FBT (A4), DFBT (A5), IID (A6), DPP (A7), DBTSO (A8) and DET (A9) units (Figure 2a) were selected to elucidate the effect of electron withdrawing strength as well as the symmetry on the properties of resultant molecules. Thiophene was used as the π‐bridge due to its ability to enhance stability and improve charge carrier mobility by facilitating intramolecular charge transfer (ICT). Carbazole derivatives (D1, D2, D3), triphenyl amine (D4), pyridine (D5), and thiazine (D6) units (Figure 2b) were chosen as the end‐groups due to their polarity and presence of heteroatoms in order to increase the intermolecular interactions.

### 2.1 Theoretical Characterizations

**Figure 3:**
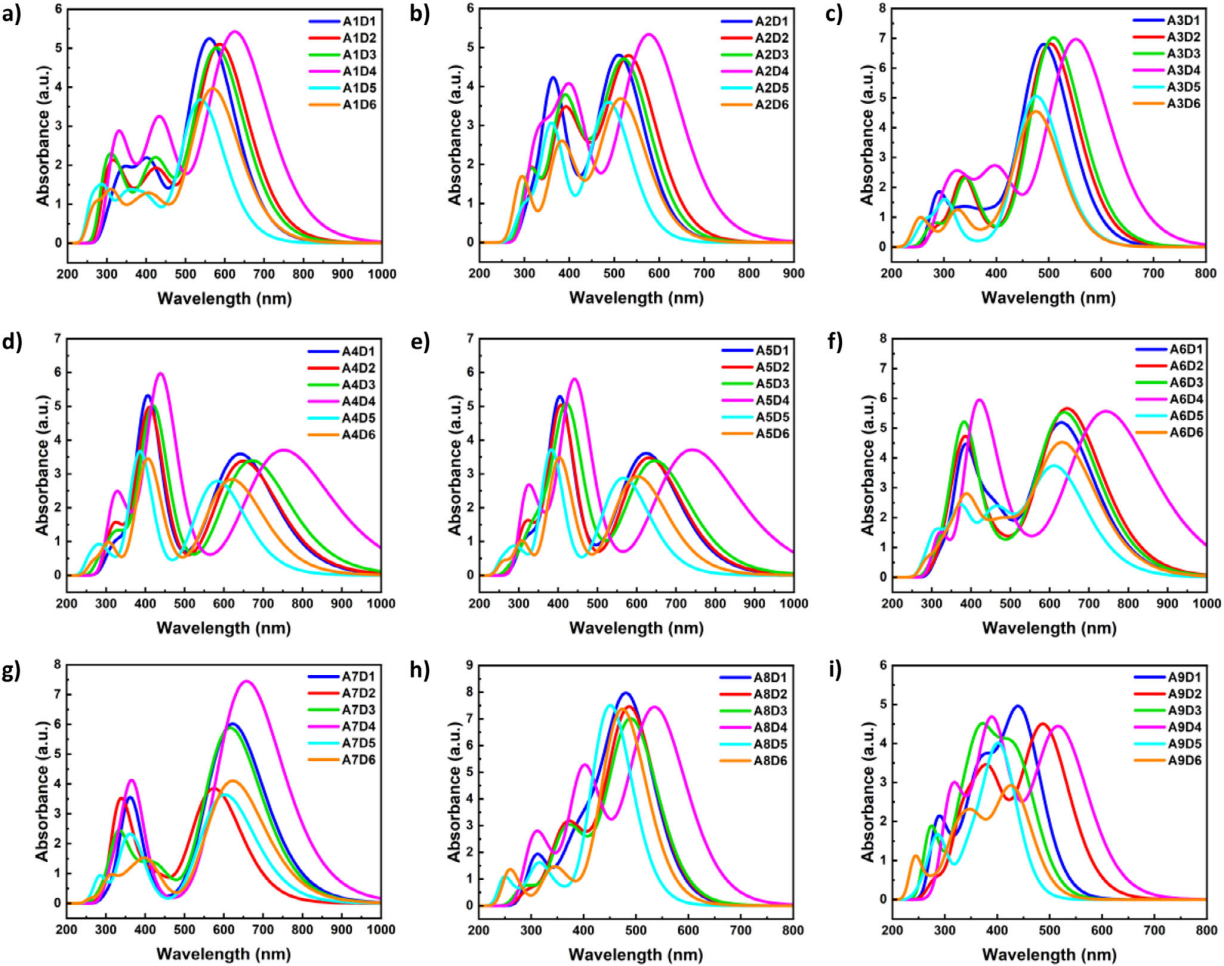
Theoretical absorption spectra of preselected fifty‐four small molecules grouped as six molecules with respect to nine different central units, namely a) A1 b) A2 c) A3 d) A4 e) A5 f) A6 g) A7 h) A8, and i) A9.

Theoretical energy levels (Table S1) and absorption spectra (Figure 3) of the small molecules, which were constructed from the selected building blocks, were determined using time‐dependent density‐functional theory (TDDFT) calculations in vacuum on a B3‐LYP/def2‐TZVP^[^ ^42^, ^43^ ^]^ level of theory. (The Graphical Representation of the optimized geometries of the small molecules is presented in Figure S2.) This is done to predict the structures presenting ideal semi‐transparent material properties, which can be defined as selective absorption in UV and NIR regions with an “optical window”. Molecules comprising A1, A2, A4, A5, A6, and A7 exhibited a strong red‐shifted absorption due to possessing strong electron acceptor units with highly electron‐withdrawing substitutions. Notably, the D4 unit shifted the absorption of the resulting molecules further to the NIR region when combined with all nine central units as a result of its low oxidation potential. Moreover, molecules bearing A2, A4, A5, A6, and A7 units presented the desired selective absorption with an “optical window”. (Figure 3b,d,e,f, and g).^[^ ^17^ ^]^ This can be attributed to restricted transitions between involved orbitals as a result of symmetry constraints. It is also observed that the “optical window” widens as the core unit is substituted in the sequence A2 < A4 < A5 < A6 < A7.

**Figure 4:**
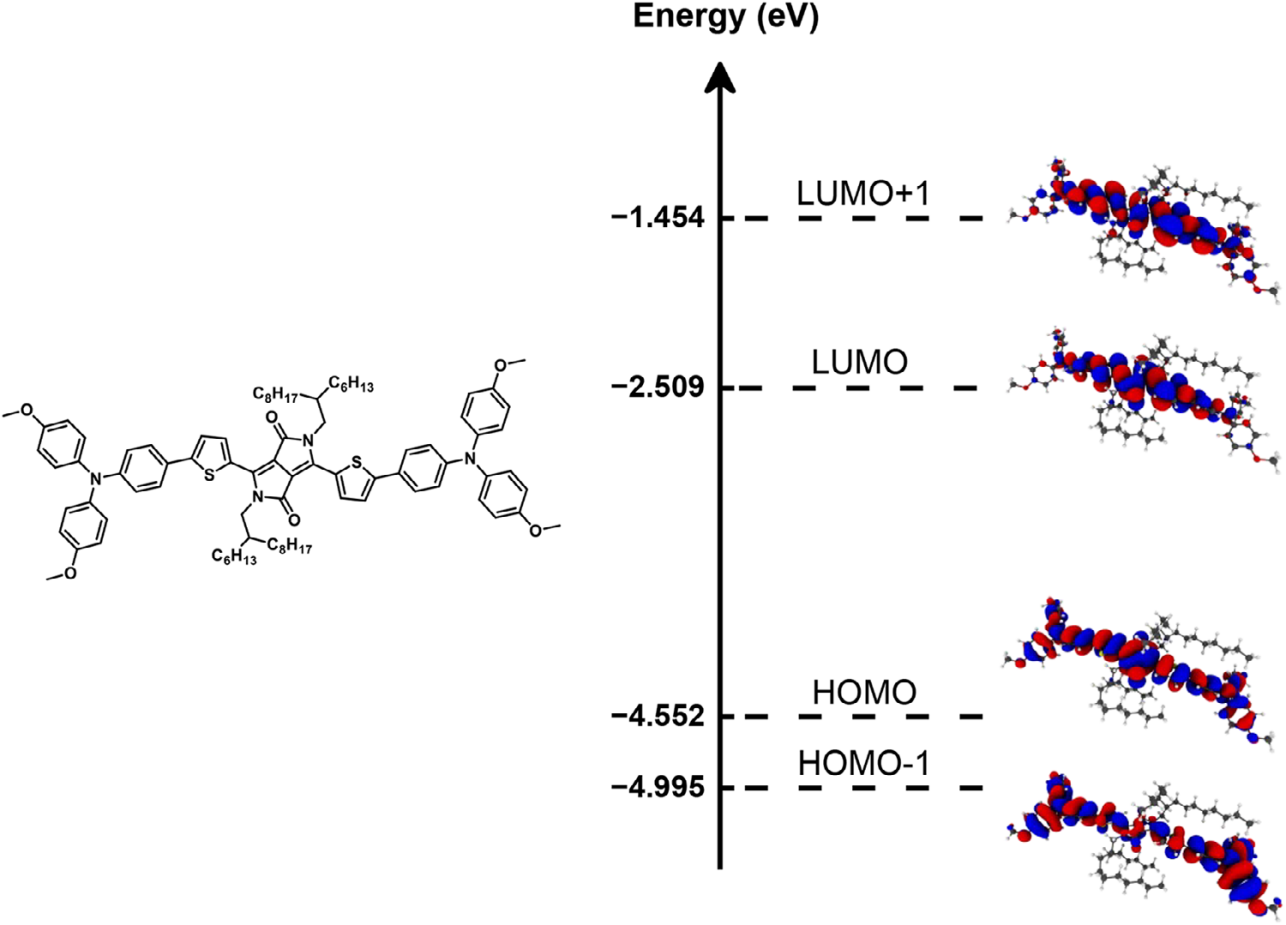
Structure and calculated energy level diagram for the frontier molecular orbitals of A7D4 small molecule.

When the symmetry of frontier orbitals is considered, it can be seen that the small molecules comprising highly symmetric central units have a symmetry‐reduced oscillator strength for some excitations between the S_0_‐S_1_ transition and higher S_0_‐S_n_ transitions, more towards UV wavelengths. (Details of excitations and pertinent oscillator strengths of all the small molecules can be found in Figures S3–S26.) While for all molecules investigated here, the HOMO to LUMO transition is allowed because HOMO and LUMO orbitals have different symmetries, which is required for a non‐zero oscillator strength, which is proportional to ⟨phi1|d|phi2⟩ where phi1 and phi2 are the involved orbitals and d is the dipole operator. In many cases, the HOMO‐1 to LUMO as well as the HOMO to LUMO+1 transition is not allowed due to having identical symmetries. (Structure and calculated energy level diagram for the frontier molecular orbitals of A7D4 small molecule is provided in Figure 4 as an example.) In more detail, building blocks A2, A5, and A8 exhibit plane and rotation symmetry, which causes the frontier orbitals to also be either plane or rotation symmetric. Depending on whether the symmetry is alternating among the frontier orbitals (e.g., in most A2Dx molecules) or not (e.g., in most A4Dx molecules, where LUMO and LUMO + 1 both have a plane symmetry), the gap in the absorption spectrum comprises of the S_2_ and S_3_ excitations or only of the S_2_ excitation. A1, A3, and A4 have in principle the same types of symmetries, but asymmetric side‐group substitution leads to a small symmetry‐breaking effect which can increase the oscillator strength and thus the absorption also for the symmetry forbidden transitions. Building blocks A6, A7, and A9 have rotation and inversion symmetries, and so do the frontier orbitals of the molecules with those building blocks. Additionally, the symmetry of the frontier orbitals can alternate or not, causing more or less symmetry forbidden excitations above the S_1_ state. Therefore, overall, a combination of the electron‐withdrawing strengths of the central units (shifting the S_1_ transition) as well as the order of frontier orbital symmetries of the central units (determining the number of symmetry‐forbidden transitions above the S_1_ transition) can be pinpointed as the reason for the widening of the “optical window” in the sequence A2 < A4 < A5 < A6 < A7.

### 2.2 Structural Characterizations

**Figure 5:**
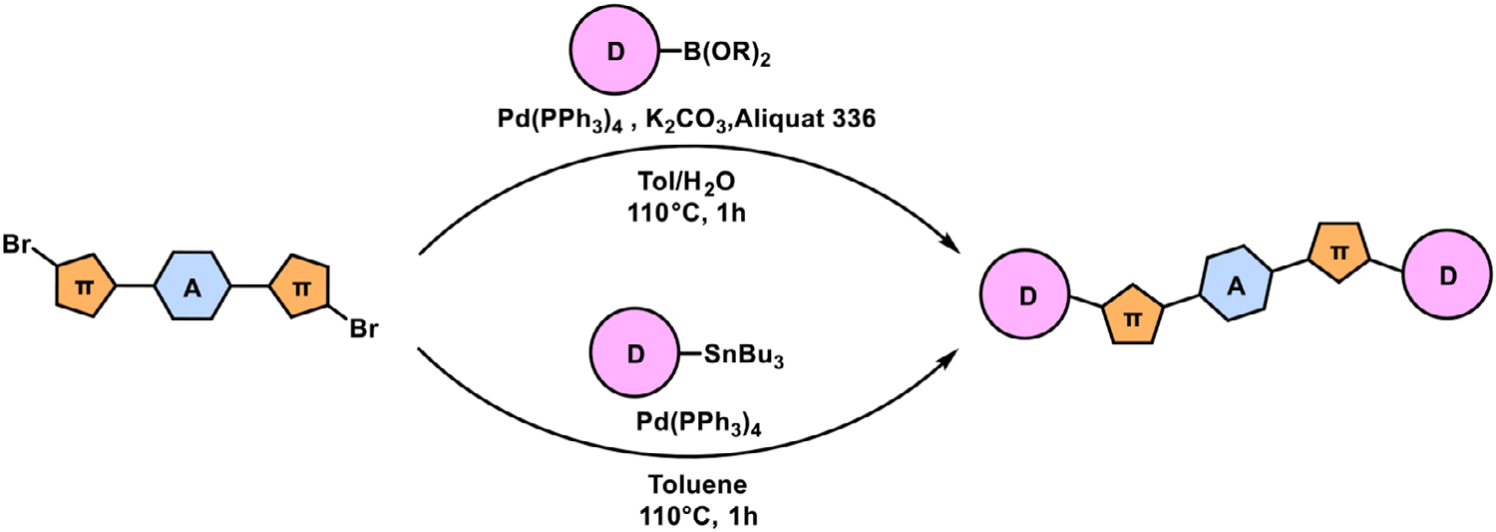
Synthetic pathways of the D‐π‐A‐π‐D type small molecules via Suzuki‐coupling and Stille‐coupling reactions.

For synthesis, twenty‐four small molecules incorporating A2, A4, A5, and A7 central units were chosen as they exhibited the desired calculated absorption spectra and facilitated a one‐step semi‐automated synthesis workflow consisting of microwave‐assisted palladium‐catalyzed cross‐coupling reactions (Figure 5). All the synthesized molecules were characterized by ^1^H nuclear magnetic resonance (NMR) spectrometry, matrix‐assisted laser desorption ionization mass spectrometry (MALDI‐MS), and elemental analysis to determine their purity. Remarkably, the compounds could be measured without the need for a matrix. This suggests that all the materials synthesized in this library undergo intermolecular electron transfer ionization when subjected to irradiation from the MALDI laser.^[^ ^44^ ^]^ (Characterization data for the small molecules, including NMR, MALDI‐TOF‐MS, and elemental analysis, are provided in the Supporting Information.)

### 2.3 Electrochemical Characterizations

**Figure 6:**
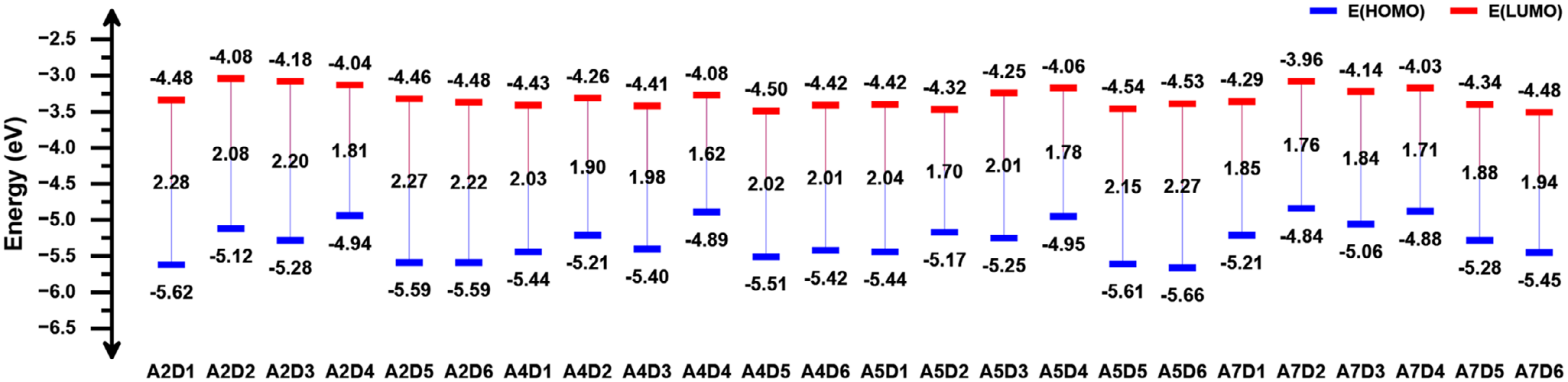
Energy level diagram of the synthesized twenty‐four small molecules representing HOMO and LUMO energy levels and energy gaps.

Electrochemical characterization of the small molecules was performed via cyclic voltammetry (CV) in order to determine their highest occupied (HOMO) and lowest unoccupied (LUMO) molecular orbital energy levels and electronic energy gaps (Figure 6). It was determined that molecules comprising the D4 unit as the end group have high‐lying HOMO levels, which consequently contribute to reducing their energy gap. The HOMO levels were calculated for A2D4, A4D4, A5D4, and A7D4 as −4.94, −4.89, −4.95, and −4.88 eV, respectively. This could be attributed to the low oxidation potential of the TPA unit, which leads to a lower band gap when combined with electron‐deficient molecules.^[^ ^45^ ^]^ When considering the average energy gaps of the small molecules with different central units, it was seen that the energy gap decreases in the order of A2 > A5 > A4 > A7. This can be explained due to the differing electron‐withdrawing strengths of these units, even though they all exhibit relatively strong electron‐withdrawing characteristics, facilitating electron delocalization and demonstrating significant intramolecular charge transfer (ICT). Specifically, the A2 unit exhibits a more localized electron‐withdrawing effect through its carbonyl substitutions. In contrast, the A4 and A5 units have stronger electron‐withdrawing effects, which are primarily localized on their thiadiazole and fluorine functional groups. When compared, it can be seen that A7, with its more extensive conjugation in the centrosymmetric lactam groups, exhibits higher electron delocalization, which results in a narrower energy gap.

### 2.4 Optical Characterizations

**Figure 7:**
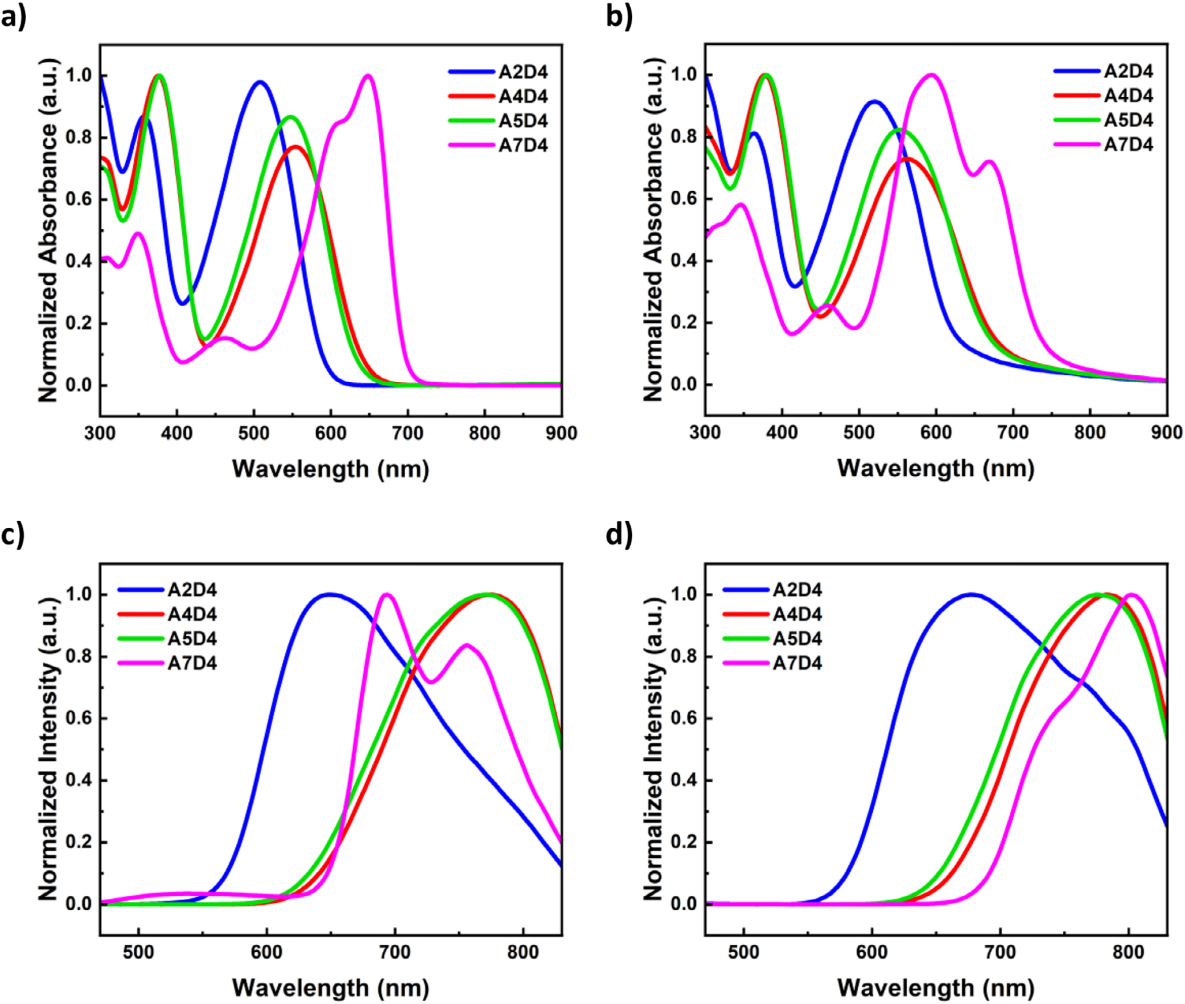
UV–vis–NIR spectra of small molecules, A4D4, A5D4 and A7D4 a) as 0.1 mg mL^−1^ solution in chlorobenzene, b) as film on glass substrates; photoluminescence (PL) spectra of small molecules A2D4, A4D4, A5D4 and A7D4 c) as 0.1 mg mL^−1^ solution in chlorobenzene, d) as film on glass substrates via excitation at 420 nm.

High‐throughput UV–vis–NIR and photoluminescence (PL) spectroscopy measurements were conducted on the small molecules to determine their optical properties in both solution and solid states. The absorption spectra of the molecules, named as A2D4, A4D4, A5D4, and A7D4, are presented in Figure 7a,b for comparative analysis. (Absorption spectra of all the synthesized materials are displayed in Figure S33.) The normalized UV–vis–NIR spectra of small molecules incorporating the A2 unit exhibited two high‐intensity absorption peaks in the range of 330–510 nm. The peaks between 330–360 nm can be attributed to π−π* transitions, and the peaks between 440–510 nm are the result of ICT. Similarly, the spectra of molecules bearing A4 also showed two strong absorption bands between 343–554 nm. The peaks in the range of 343–375 nm corresponded to π−π* transition, and those between 473–554 nm can be ascribed to ICT. The spectra of the A5 small molecules also revealed two prominent absorption bands within the range of 340–550 nm. Peaks in the 340–377 nm range can be assigned to π−π* transitions, while those in the 463–550 nm range are associated with ICT. The spectra of molecules featuring the A7 unit displayed two strong absorption bands along with a smaller band between 327–650 nm. The peaks within the range of 327–353 nm were attributed to π−π* transition, those in the 410–460 nm range corresponded to n−π* transition, while peaks between 606–650 nm are related to ICT. It was seen that the optical band gap is decreased and the absorption band undergoes a redshift as the central unit is changed in the sequence A2 > A4 ≅ A5 < A7.^[^ ^24^, ^25^ ^]^

In line with TDDFT calculations, “optical window” broadens as the core unit is changed from A2 to A4 to A5 to A7, with widths of 100 nm for A2, 150 nm for A4 and A5, and 250 nm for A7 small molecules. This relates to the symmetry‐reduced oscillator strength for some excitations between the S_0_‐S_1_ transition and higher S_0_‐S_N_ transitions as a result of symmetry constraints. Consequently, the widening of the “optical window” in the sequence A2 < A4 ≅ A5 < A7 can be attributed to the combined electron‐withdrawing strengths of the building blocks, along with the symmetry of the frontier orbitals of the central units. In addition, the D4 unit was found to cause a redshift in the absorption maxima when combined with all the central units that were used in the synthesis. This effect can be attributed to its low oxidation potential and strong electron‐donating character, which may consequently enhance intramolecular charge transfer (ICT), particularly when combined with strong central acceptor units. This is caused by the presence of electron‐rich heteroatoms and effective conjugation that allows versatile electron donation. In contrast to D4, the D5 and D6 units caused wider energy gaps and a blue shift in the absorption due to their relatively weak electron‐donating abilities. The UV–vis–NIR absorption spectra of thin films of the small molecules A2, A4, and A5 closely resembled their corresponding spectra in solution. In contrast, the absorption spectra of A7 in thin film differed significantly from its solution‐phase spectra. In thin films, A7 exhibited a broader absorption band with higher intensity at shorter wavelengths, while the peak at the longest wavelength was red‐shifted and of lower intensity. This behaviour suggests the formation of H‐aggregates in the A7 molecules.

In Figure 7c,d, the normalized PL spectra of the small molecules, named as A2D4, A4D4, A5D4, and A7D4, are displayed for comparative investigation. (PL spectra of all the synthesized molecules can be found in Figure S34.) They showed that materials have mirrored images of the S_0_‐S_1_ absorption, suggesting similar vibrational levels in both electronic states. The emission maxima of the molecules were determined in the ranges of 562–650 nm, 586–773 nm, 570–771 nm, and 635–692 nm for the molecules comprising A2, A4, A5, and A7 units, respectively. Consistent with the absorption spectra of the molecules, solid‐state emission spectra exhibited a red shift as the central unit was varied in the order A2 > A5 > A4 > A7. Similarly, the D4 unit was found to induce a redshift in the emission maxima in comparison to the molecules bearing the other end units. (Absorption maxima λ _max, abs_, emission maxima λ _max, em,_ and optical energy gap values of all synthesized small molecules are summarized in Table S2.)

**Figure 8:**
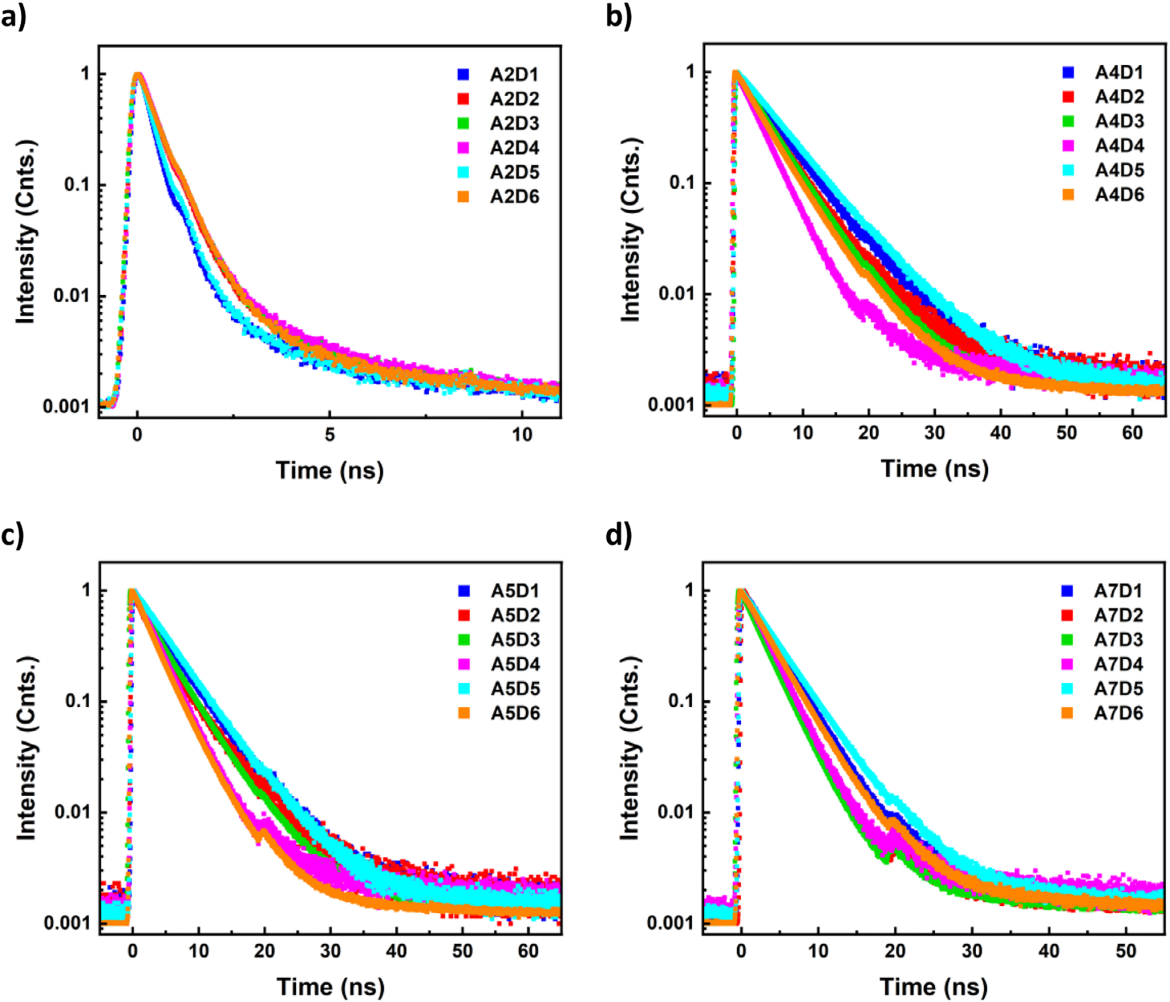
Time‐resolved photoluminescence decay of small molecules with a) A2 unit, b) A4 unit, c) A5 unit, and d) A7 unit as a 8 mg mL^−1^ solution in chlorobenzene.

Time‐resolved photoluminescence measurements of the small molecules in chlorobenzene solution were performed to determine excited‐state lifetimes and are represented in Figure 8. Molecules with the same core unit exhibited similar excited state lifetimes. Lifetimes of the materials were reported in Table S3. Excited state lifetimes are longer for molecules comprising the A4 unit (between 3.51 and 6.10 ns) and slightly shorter for A5 molecules (between 3.27 and 5.15 ns) while A7 molecules displayed even shorter lifetimes (between 2.95 and 4.30 ns) and molecules with A2 unit exhibited the shortest excited‐state lifetimes (between 0.34 and 0.53 ns) of all small molecules. The longer excited‐state lifetime of molecules with A4 and A5 units might be attributed to the molecular rigidity, electron delocalization, and heavy atom effect on benzothiadiazole structure, while shorter decay lifetimes of A7 and A2 might be caused by their flexible molecular structures that facilitate non‐radiative decay processes, vibrational coupling and potential for intersystem crossing.

## 3 Conclusion

This study developed a workflow to screen the optoelectronic properties of a subset of organic small molecules, aiming to identify structures that exhibit ideal semi‐transparent material properties. We generated a subset of fifty‐four materials using literature‐based preselection, followed by TDDFT studies to determine their theoretical energy levels and absorption spectra. This approach facilitated narrowing down the pool of materials for further investigation. From this subset, thirty molecules exhibited relatively narrow energy gaps and redshifted absorption maxima. These properties suggest potential for selective photon capture, while offering an “optical window” that could enhance transparency. Twenty‐four of these molecules were subsequently synthesized using microwave‐assisted palladium‐catalysed cross‐coupling reactions. Their energy levels, energy gaps, optical properties, and excited‐state lifetimes were then characterized. We observed that altering the central unit in the sequence A2 > A5 > A4 > A7 resulted in a narrower energy gap and a redshift in the absorption spectra. The “optical window” also widened as the core unit transitioned from A2 to A4, A5, and A7. The redshift and increase in the “optical window” in the sequence A2 < A4 < A5 < A7 can be attributed to the shifting of the S_1_ transition to higher wavelengths and restricted transitions between the involved orbitals. This is believed to be a result of electron‐withdrawing strengths and the symmetry constraints of the central units. Notably, the molecules containing the A7 unit exhibited the most red‐shifted and wavelength‐selective absorption, featuring a 250 nm‐wide “optical window” that enables enhanced transparency. Additionally, the formation of H‐aggregates was observed in their absorbance in solution. Both PL and TRPL studies demonstrated effective charge carrier formation and transport in these small molecules, a crucial characteristic for their potential applications in optoelectronic devices. TRPL results indicated that while molecules with A4 and A5 units exhibited longer excited‐state lifetimes, those with the A7 unit displayed comparable lifetimes. Overall, this research presents an effective workflow for material discovery and characterization that identifies target molecules for optoelectronic applications with enhanced transparency.

## 4 Experimental Section

### Theoretical calculations

Initial geometries of all molecules in the combinational library were generated from SMILES codes using RDKit. Conformers were generated based on the initial geometries using the CREST software package^[^ ^46^ ^]^ based on the GFNFF level of theory. The lowest energy conformer was optimized using GFN2‐xTB^[^ ^47^ ^]^ and then further optimized using density functional theory on a B3‐LYP/def2‐TZVP^[^ ^42^, ^43^ ^]^ level of theory using the TURBOMOLE^[^ ^48^ ^]^ package and ASE.^[^ ^49^ ^]^ Excited states were calculated using TDDFT^[^ ^50^ ^]^ on the same level of theory. Excited state energies and corresponding oscillator strengths with a constant broadening were used to obtain absorption spectra. The main contributing molecular orbitals of the selected transitions were analysed and visualized using VMD to investigate their symmetries. Theoretically calculated values of HOMO and LUMO energy levels of the small molecules are summarized in Table S1.

### General procedure for suzuki‐coupling reaction between dibromo arenes (A2, A4, A5 and A7) and boronic acid/acid ester arenes (D1, D2, D3, D4, D5)

Dibromo arene (A) (1 eq.), boronic acid/acid ester arene (D) (2.5 eq.), tetrakis(‐triphenylphosphine) palladium (0) (0.1 eq.), K_2_CO_3_ (2.5 m, 10 eq) and aliquat 336 (1–2 drops) were taken into a 10 mL pyrex vessel. Toluene (3 mL) was also added to the vessel to dissolve the mixture, and the cap of the vessel was sealed in the glove box under an inert atmosphere. The reaction mixture was stirred and purged with N_2_ at room temperature for 15 min. Then, the mixture was irradiated at 110 °C for 1 h using a CEM Discover 2.0 Microwave Reactor by moderating the initial power at 150 W. After the reaction was completed, the organic phase was separated from the aqueous phase via decantation, and the organic phase was concentrated in a sample concentrator. The crude product was purified with column chromatography on silica gel filled in 20 mL syringe cartages using the vacuum manifold in a gradient solvent system of hexane/chloroform. Small molecules with lower solubility, namely A4D1, A4D2, A5D1, and A5D2 were purified via recrystallization through dissolution in chloroform and precipitation over hexane.

### General procedure for stille‐coupling reaction between dibromo arenes (A2, A4, A5 and A7) and stannyl arenes (D6)

Dibromo arene (A) (1 eq.), stannyl arene (D) (2.5 eq.) and tetrakis(‐triphenylphosphine) palladium (0) (0.1 eq.) were added into a 10‐mL pyrex vessel. The mixture was dissolved by adding toluene (3 mL) into the vessel inside a glove box under an inert N_2_ atmosphere, and the cap of the vessel was sealed. Next, the reaction mixture was stirred and purged with N_2_ at room temperature for 15 min. Then, the mixture was irradiated with an initial power of 150 W at 110 °C for 1 h using a CEM Discover 2.0 Microwave Reactor. The crude product was purified through column chromatography on silica gel filled in 20 mL syringe cartages using the vacuum manifold in a gradient solvent system of hexane/chloroform. (The synthesis details of the small molecules are provided in the Supporting Information).

### Electrochemical measurements

Electrochemical characterization of the small molecules was performed via cyclic voltammetry (CV) in a three‐electrode cell system where the ITO‐coated glass substrate was used as the working electrode, Pt wire as the counter electrode, and Ag/Ag^+^ as the reference electrode in 0.1 m electrolyte solution of tetrabutylammonium hexafluorophosphate/acetonitrile (NBu_4_PF_6_/ACN). Solutions of the materials were prepared by dissolving them in DCM, and their films were prepared on indium tin oxide (ITO)‐coated glass substrate via drop casting the solutions and followed by annealing at 50 °C. Potential was applied with a scan rate of 100 mV s^−1^ via Ossila Potentiostat in a triangular waveform, while the current is plotted against the applied potential. Cyclic voltammograms were plotted via automated fitting, and onsets of first oxidation and reduction potentials were determined via the fitting (Figures S27, S28, S29, and S30). The HOMO and LUMO energy levels of the molecules were calculated from the onsets of first oxidation and reduction potentials, respectively. (Detailed calculation can be found in Supporting Information.) The values of onsets of first oxidation and reduction potentials, and HOMO and LUMO energy levels, and the electronic energy gaps of the molecules are summarized in Tables S1 and S2 and shown in Figure 6. Experimentally obtained and theoretically calculated HOMO and LUMO energy levels were compared and presented in Figure S31.

### Optical measurements

High‐throughput UV–vis–NIR and photoluminescence (PL) spectroscopy measurements of the small molecules were performed both in solution and on film by the TECAN platform with a Microplate Reader infinite 200 Pro. In order to perform solution‐state measurements, 0.1 mg mL^−1^ solutions of all molecules were prepared in chlorobenzene, and 20 µL of each was added on a well plate. Thin films were prepared using 8 mg mL^−1^ of solution of the small molecules in chloroform and spin‐coating them on glass substrates with a Spinbot at 1000 rpm and followed by an annealing step at 120 °C in order to conduct solid‐state measurements (Films of the molecules are presented in Figure S32). UV–vis–NIR and PL spectroscopies of both solutions and films were measured automatically on a Tecan plate reader. Electronic, optical, and theoretically calculated energy gap values are compared and shown in Figure S35.

### Time‐Resolved Photoluminescence (TRPL) measurements

Solution of small molecules with a concentration of 8 mg mL^−1^ in chlorobenzene was prepared, and 0.1 mL of them were taken to a well‐plate. Time‐resolved photoluminescence measurements were then performed with a custom‐built high‐throughput characterization setup based on a Picoquant FluoTime300 photoluminescence spectrometer. Excitation was provided by a 402nm LDH‐P‐C‐405B pulsed nanosecond laser, coupled to an optical Y‐fiber to direct the laser onto the sample. The emitted luminescence was collected by a lens and directed through the same fiber and a 435 nm longpass filter to the detector of the FluoTime300. The luminescence signals were recorded in time‐correlated single photon counting mode at a wavelength of 402.2 nm and with a bin width of 25 ps, with a laser repetition rate of 10.0 MHz.

Due to the mostly mono‐exponential behaviour of the data (as seen by the linear slope in the semilogarithmic plot), the recorded signals were subsequently fit with a mono‐exponential decay function of the form $I\;\left(t\right)=\;A*e^{-\frac{t}{\tau }}$ using the Picoquant FluoFit software. Here, A is the amplitude of the signal, and τ is the charge carrier lifetime. Importantly, the additional peak found in the signals after roughly 20 ns is an artefact of the setup, produced by imperfect outcoupling of the light from the fiber. This leads to a reflection at the end of the fiber and thus an additional delayed signal.

## Author Contributions

E.A.A. contributed to the conceptualization, investigation, and writing of the original manuscript. E.A.A. performed the synthetic works, electrochemical characterization, and optical characterization. H.M. and P.R. performed the theoretical calculations. P.F. helped to analyse the theoretical data and contributed to writing and reviewing the manuscript. C.K. performed the TRPL analysis and contributed to writing. J.S.R.O. conducted the MALDI‐TOF MS and elemental analysis and contributed to writing. A.B. provided revisions on the manuscript. M.B., J.A.H., and C.J.B. provided supervision and revised the manuscript. All authors contributed to the discussion of experimental results and the manuscript.

## Conflict of Interest

The authors have no conflict of interest to declare.

## Supporting information



Supporting Information

Supporting Information

## Data Availability

The data that support the findings of this study are available in the supplementary material of this article.
