# Supplementary material for: Tuning the Transparency and Exciton Transition of D‐π‐A‐π‐D Type Small Molecules
Source: Chemistry. 2025 Jul 22;31(45):e00657. doi: 10.1002/chem.202500657 (PMC12351442; doi:10.1002/chem.202500657)
Supplement: Supplementary file 1 — Supporting Information [file CHEM-31-e00657-s002.docx]

Tuning the Transparency and Exciton Transition of D-π-A-π-D Type Small Molecules

Ecem Aydan Alkan,* Houssam Metni, Patrick Reiser, Christian Kupfer, Juan S. Rocha-Ortiz, Anastasia Barabash, Miroslaw Batentschuk, Jens A. Hauch, Pascal Friederich, Christoph J. Brabec*

E. Aydan Alkan, C. Kupfer, J. S. Rocha-Ortiz, A. Barabash, M. Batentschuk, J. A. Hauch, C. J. Brabec

Friedrich-Alexander-Universität Erlangen-Nürnberg

Department of Materials Science and Engineering

Institute of Materials for Electronics and Energy Technology (i-MEET)

Martensstraße 7, 91058 Erlangen, Germany
E-mail: [ecem.alkan@fau.de](mailto:ecem.alkan@fau.de); [christoph.brabec@fau.de](mailto:christoph.brabec@fau.de)

E. Aydan Alkan, C. Kupfer, J. S. Rocha-Ortiz, J. A. Hauch, C. J. Brabec

Forschungszentrum Jülich GmbH

Helmholtz-Institute Erlangen−Nürnberg (HI-ERN)

Immerwahrstr. 2, 91058 Erlangen, Germany

H. Metni, P. Reiser, P. Friederich
Karlsruhe Institute of Technology (KIT)

Institute of Nanotechnology

Kaiserstr. 12, 76131 Karlsruhe, Germany

H. Metni, P. Reiser, P. Friederich

Karlsruhe Institute of Technology (KIT)

Institute of Theoretical Informatics

Kaiserstr. 12, 76131 Karlsruhe, Germany

1. **Materials and Methods**

All chemicals and solvents were purchased from Merck, TCI, BLD, Ossila, Biosynth or VWR and used without further purification in the synthesis and characterization unless otherwise mentioned. Reactions were performed under microwave irradiation using CEM Discover 2.0 Microwave Synthesizer. Thin-layer chromatography plates was performed using commercially available silica gel plates (Merck TLC Silica Gel 60 F_254_) for monitoring the reactions and assisting the purification processes. UV light (254 or 366 nm) purchased from Analytikjena was used for detection. Column chromatography performed using 20-mL syringes filled with Silica Gel (Fluorochem Silica Gel 60A 40-63 u) for the purification process. Silica gel in the syringe-columns were flashed with eluent under applied vacuum using Supelco Visiprep Vacuum Manifold and Kaf Laboport Vacuum Pump. Products were dried using Stuart Sample Concentrator. Structural characterizations of the compounds were performed with ^1^H nuclear magnetic resonance (NMR) spectra via Brucker Avance III 400 MHz using CDCl_3_ or DMSO as the solvent. The chemical shifts were reported in parts per million (ppm) downfield from an internal trimethylsilane (TMS) reference. Coupling constants (J) were reported in Hertz (Hz), and the spin multiplicities were specified by the symbols of s (singlet), d (doublet), t (triplet), and m (multiplet). MALDI-ToF (Smarthbeam II laser, 335 nm up to 2 kHz repetition rate) mass spectra were obtained by using a Bruker autoflex max TOF/TOF or on a Bruker ultraflex TOF/TOF spectrometer with graphite (Faber-Castell 6B pencil) or matrix free. Elemental analysis data were obtained by placing approximately 1–2 mg of the material in tin boats, which were then loaded into the autosampler of the UNICUBE elemental analyzer, equipped with a Thermal Conductivity Detector (TCD) for carbon, hydrogen, nitrogen, and sulfur, and an additional Infrared (IR) Detector for sulfur. Electrochemical studies were utilized by Ossila Potentiostat in a three-electrode cell system where indium tin oxide (ITO)-coated glass substrate as the working electrode, Pt wire as the counter electrode and A/Ag^+^ as the reference electrode in 0.1 M electrolyte solution of tetrabutylammonium hexafluorophosphate/acetonitrile (ACN). Potential applied with a scan rate of 100 mV/s in a triangular waveform and the current was plotted against the applied potential. Cyclic voltammograms were plotted and onsets of first oxidation and reduction potentials were determined via automated fitting created in our research group. Highest occupied molecular orbital (HOMO) and lowest unoccupied molecular orbital (LUMO) energy levels of molecules were calculated considering the value of NHE as −4.8 eV with respect to the vacuum level. Uv-vis-NIR and PL measurements of the small molecules were done both in solution and on film by TECAN platform with a Microplate Reader infinite 200 Pro. For solution-state measurements, 0.1 mg/mL solution of molecules were prepared in chlorobenzene. In order to prepare thin films, 8 mg/mL of solution of small molecules in chloroform were spin coated on glass substrate at 1000 rpm using Sciprios SpinBot platform and annealed at 120 °C. Glass substrates were cleaned sequentially in DI water, acetone, and isopropanol in an ultrasonic bath for 10 minutes each. PL transients were recorded with a Fluotime300 steady-state and lifetime spectrometer (PicoQuant) with a 402 nm pump laser by using solution of small molecules of 8 mg/mL in chlorobenzene. The solution-state UV-vis-NIR, PL and TRPL measurements were done in Microplate 96/U-PP well-plates purchased from Eppendorf.

1. **Theoretical Characterizations**

All theoretical properties of prescreened computed molecules were added to Supporting Information as a separate excel file.

Figure S1. Structures of 54 small molecules.

Figure S2. The Graphical Representation of the optimized geometries of the small molecules.


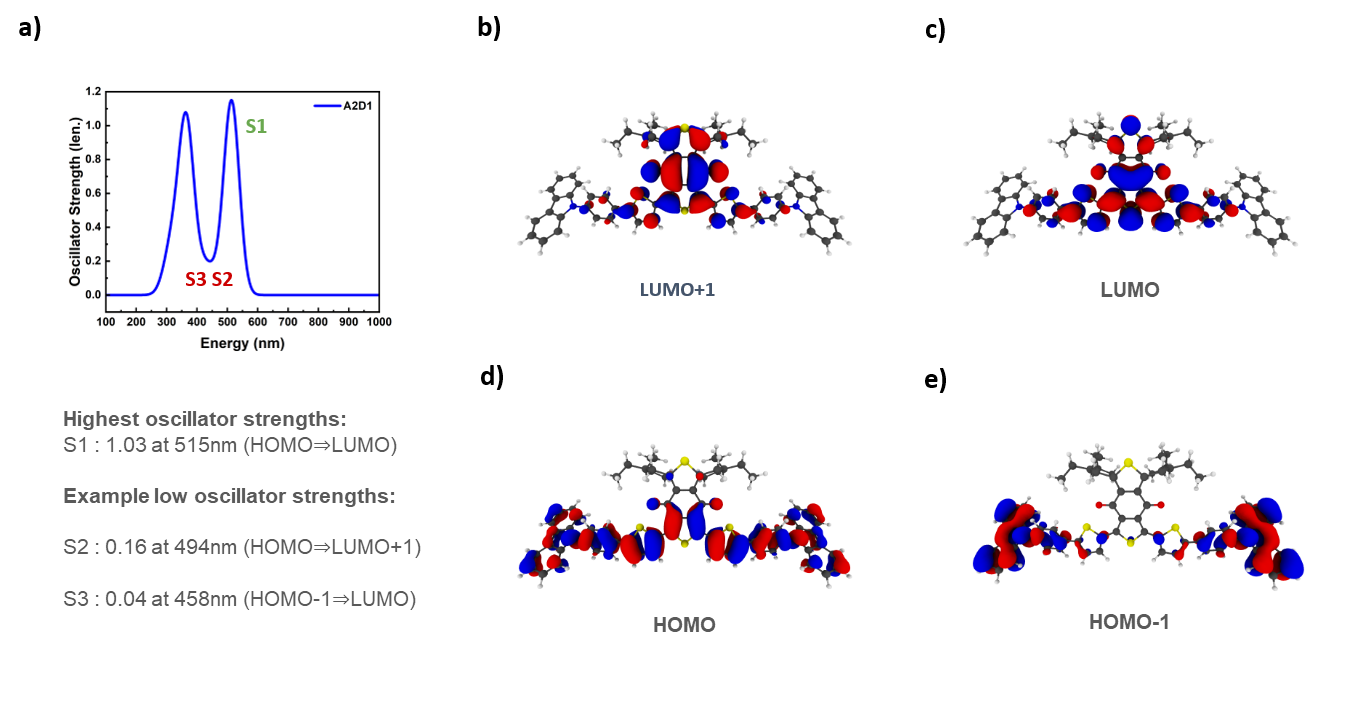


Figure S3. a) Oscillator strength versus transition energy from the ground state to higher states for the computed A2D1 molecule and graphical representation of its b) LUMO+1, c) LUMO, d) HOMO, f) HOMO-1 orbitals.


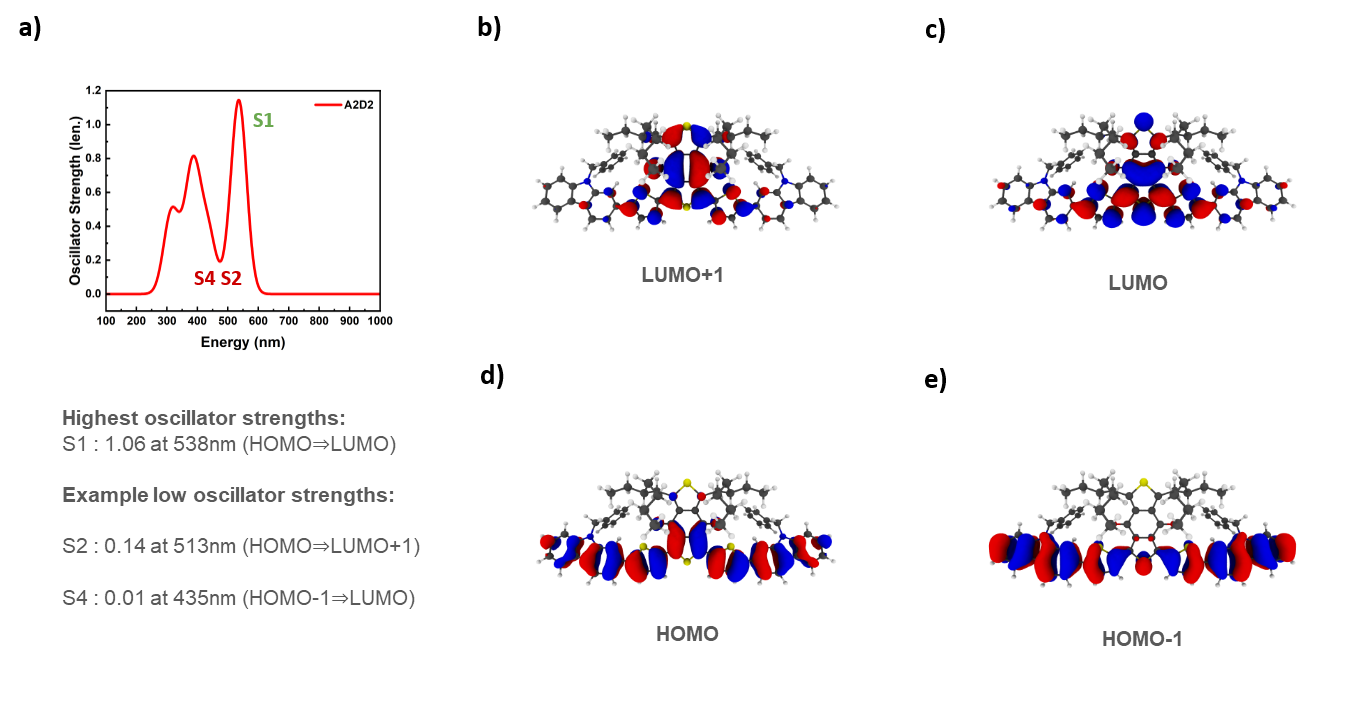


Figure S4. a) Oscillator strength versus transition energy from the ground state to higher states for the computed A2D2 molecule and graphical representation of its b) LUMO+1, c) LUMO, d) HOMO, f) HOMO-1 orbitals.


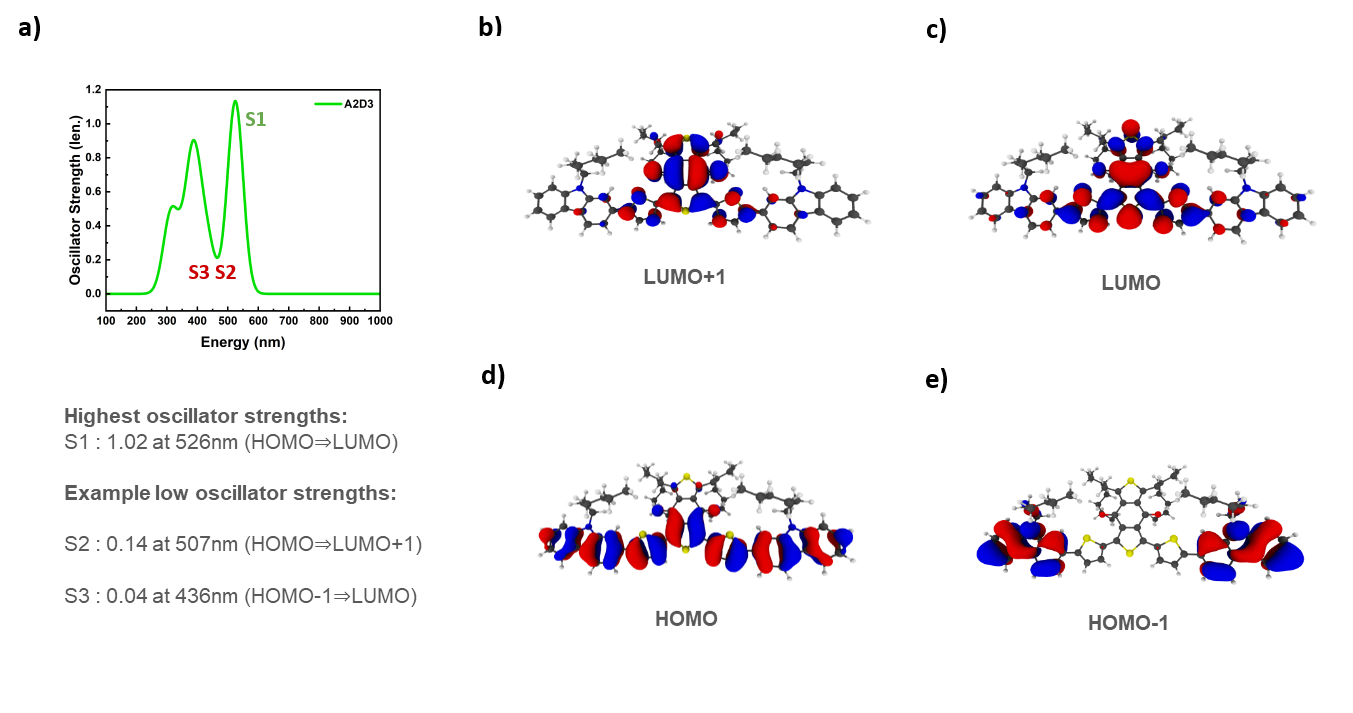


Figure S5. a) Oscillator strength versus transition energy from the ground state to higher states for the computed A2D3 molecule and graphical representation of its b) LUMO+1, c) LUMO, d) HOMO, f) HOMO-1 orbitals.


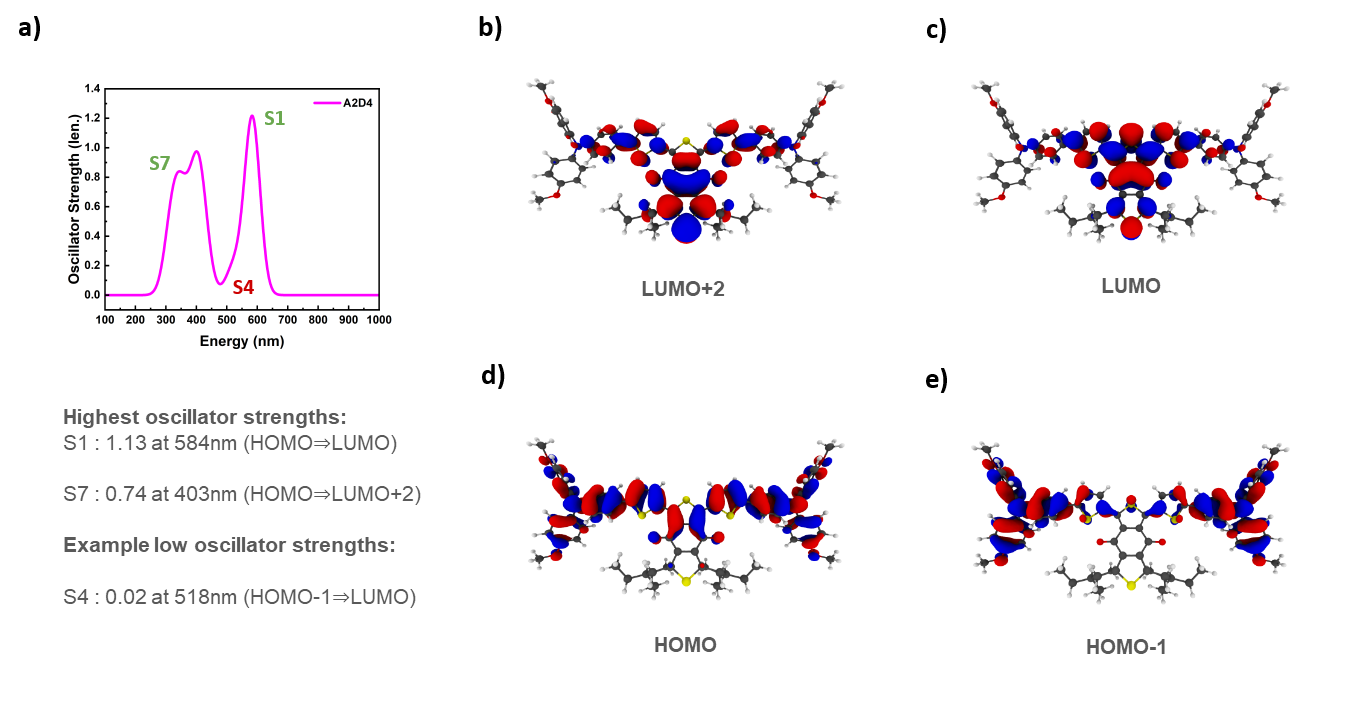


Figure S6. a) Oscillator strength versus transition energy from the ground state to higher states for the computed A2D4 molecule and graphical representation of its b) LUMO+2, c) LUMO, d) HOMO, f) HOMO-1 orbitals.


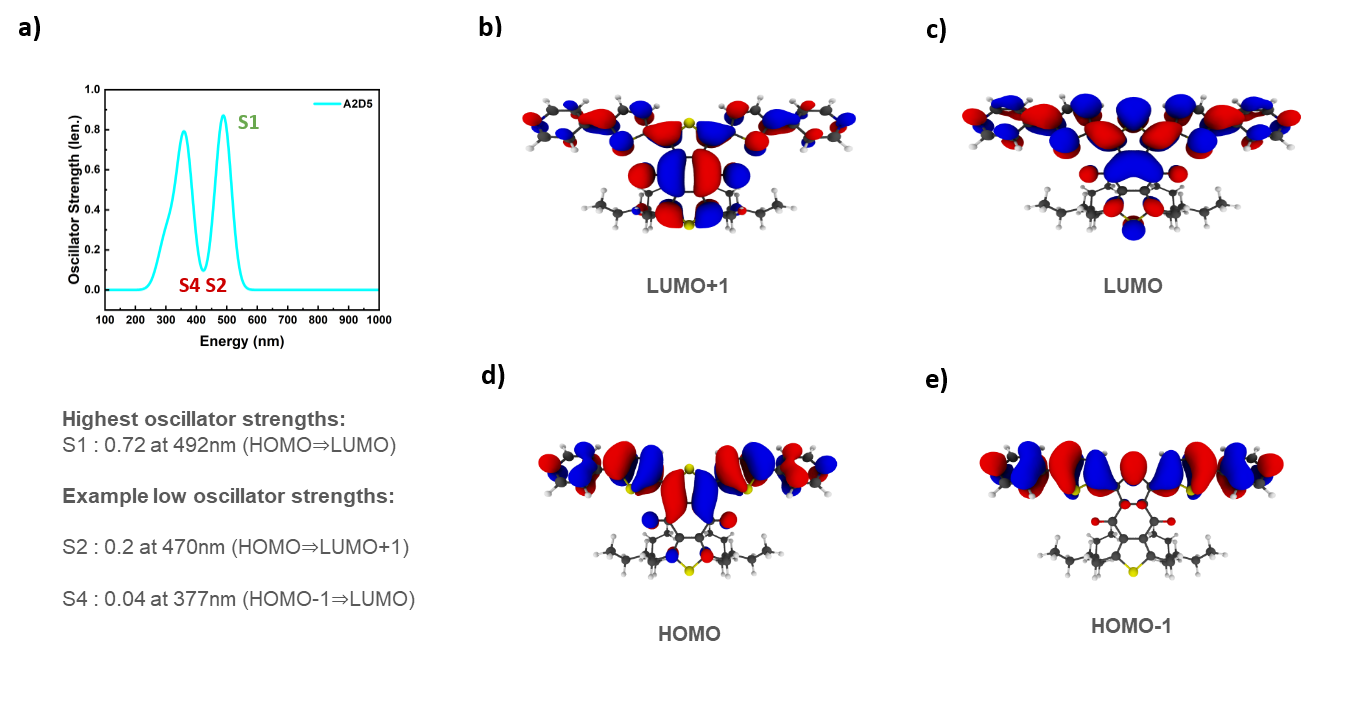


Figure S7. a) Oscillator strength versus transition energy from the ground state to higher states for the computed A2D5 molecule and graphical representation of its b) LUMO+1, c) LUMO, d) HOMO, f) HOMO-1 orbitals.


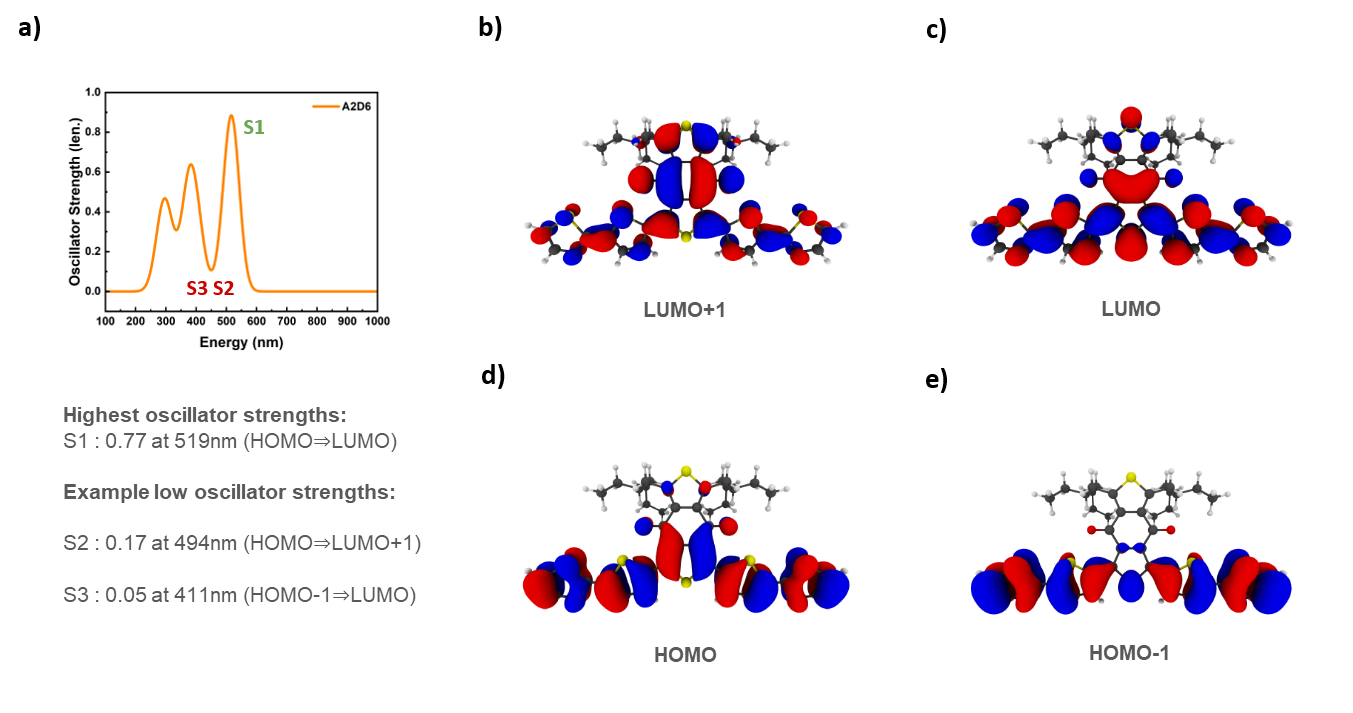


Figure S8. a) Oscillator strength versus transition energy from the ground state to higher states for the computed A2D6 molecule and graphical representation of its b) LUMO+1, c) LUMO, d) HOMO, f) HOMO-1 orbitals.


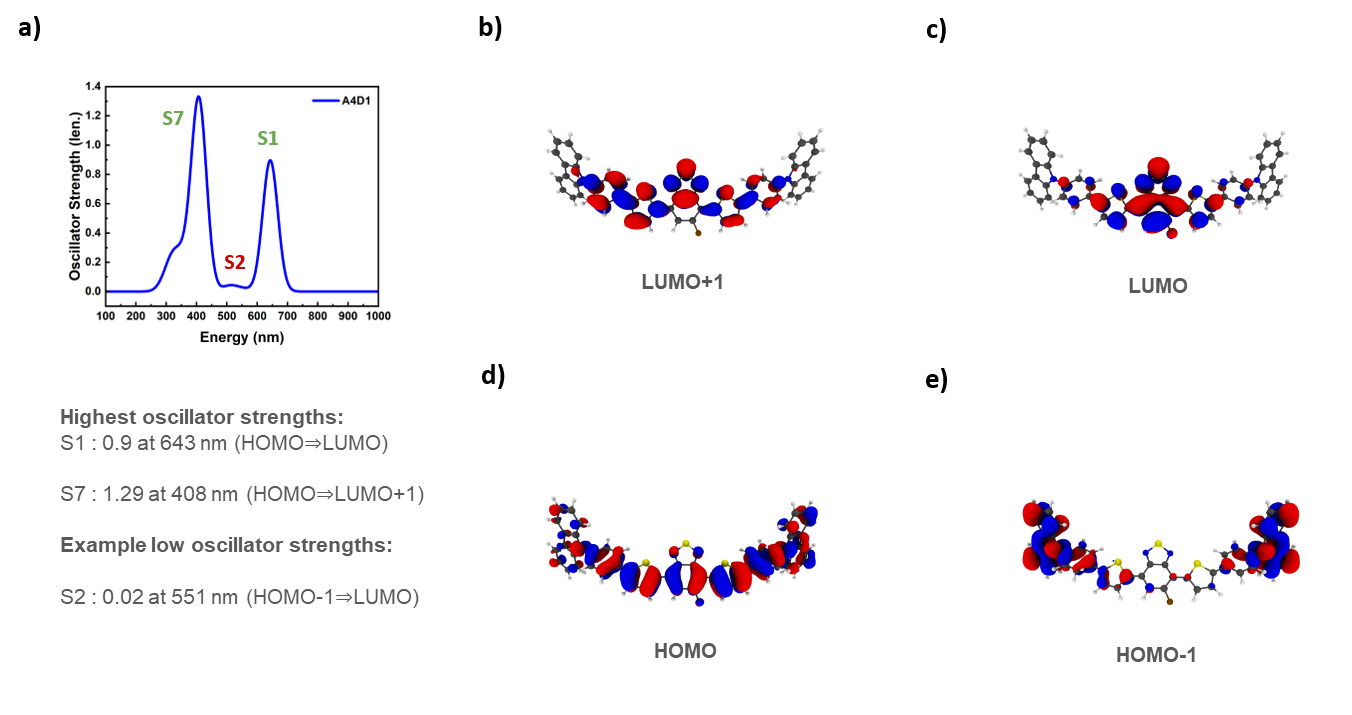


Figure S9. a) Oscillator strength versus transition energy from the ground state to higher states for the computed A4D1 molecule and graphical representation of its b) LUMO+1, c) LUMO, d) HOMO, f) HOMO-1 orbitals.


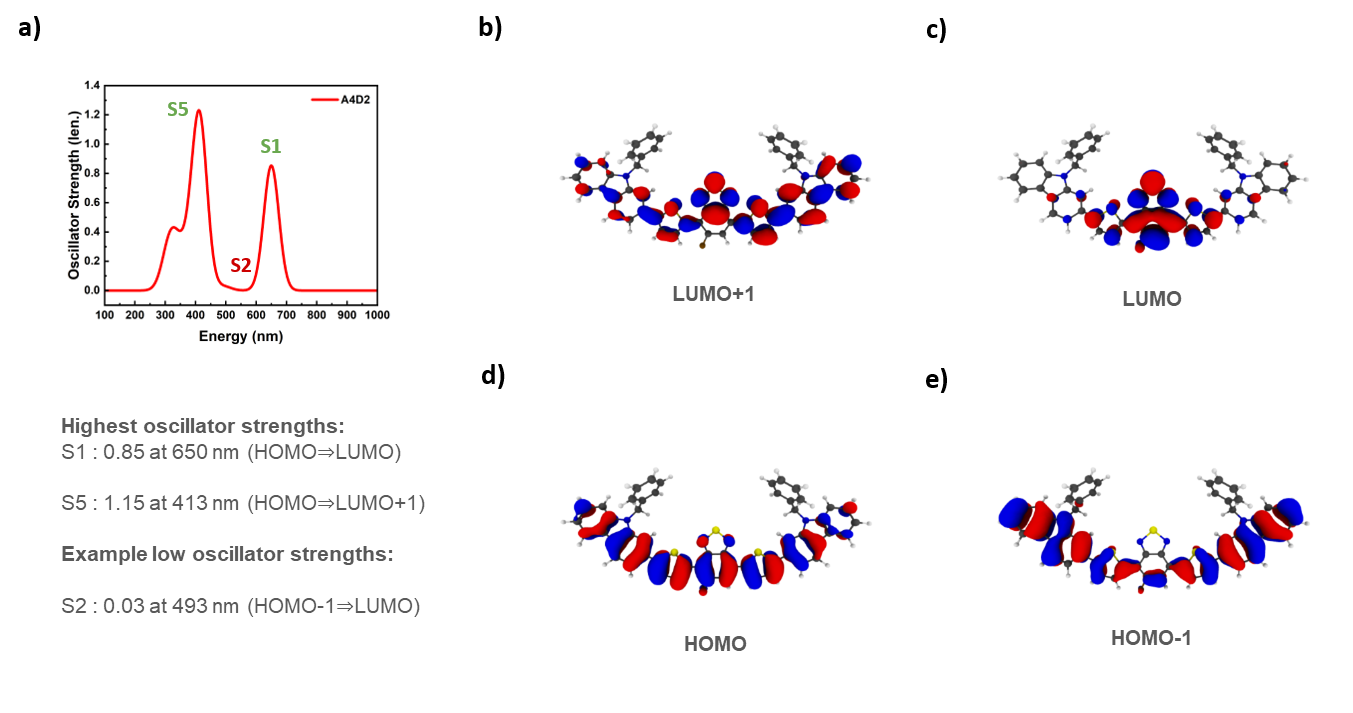


Figure S10. a) Oscillator strength versus transition energy from the ground state to higher states for the computed A4D2 molecule and graphical representation of b) LUMO+1, c) LUMO, d) HOMO, f) HOMO-1 orbitals.


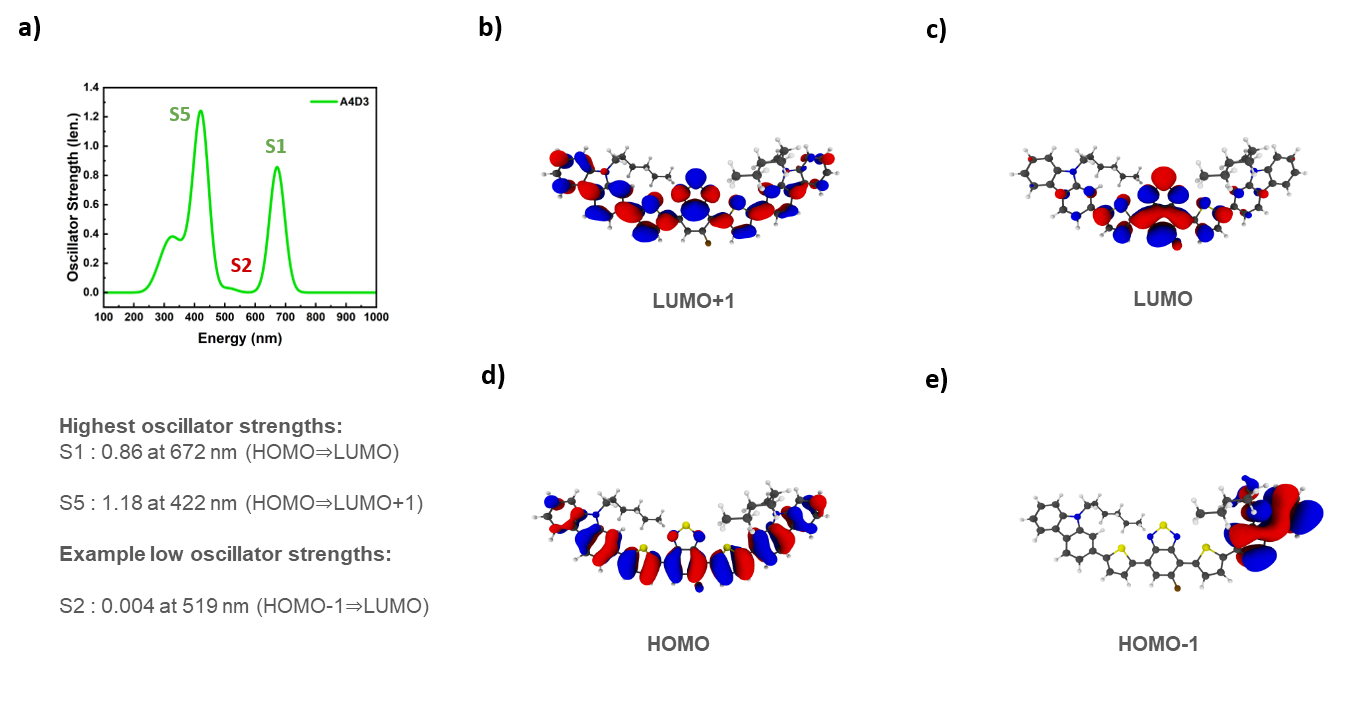


Figure S11. a) Oscillator strength versus transition energy from the ground state to higher states for the computed A4D3 molecule and graphical representation of its b) LUMO+1, c) LUMO, d) HOMO, f) HOMO-1 orbitals.


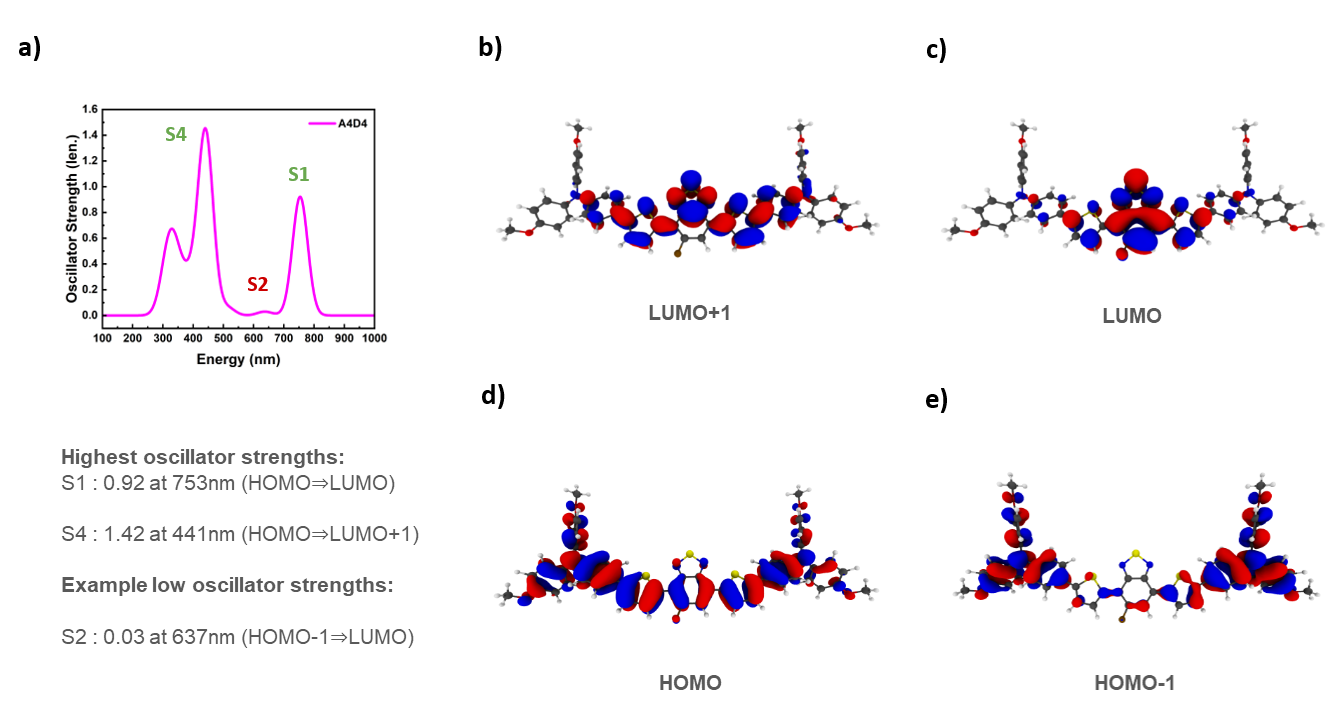


Figure S12. a) Oscillator strength versus transition energy from the ground state to higher states for the computed A4D4 molecule and graphical representation of its b) LUMO+1, c) LUMO, d) HOMO, f) HOMO-1 orbitals.


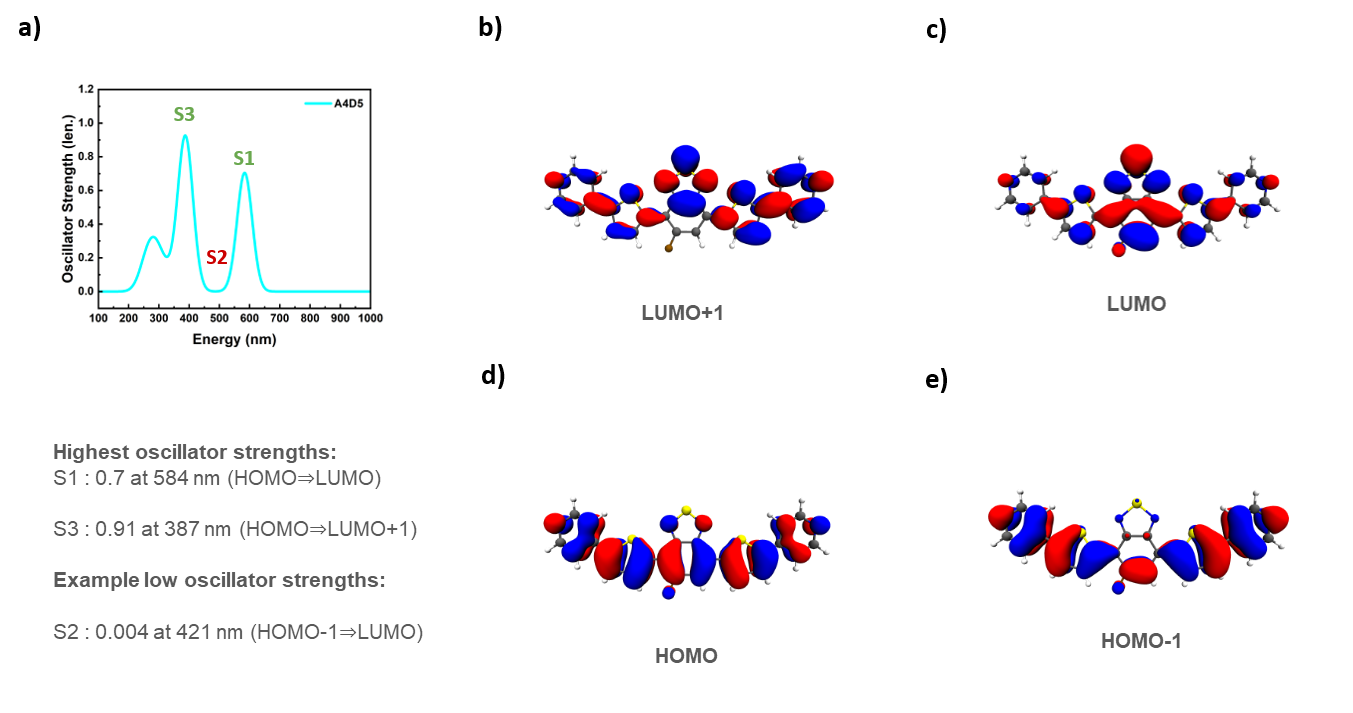


Figure S13. a) Oscillator strength versus transition energy from the ground state to higher states for the computed A4D5 molecule and graphical representation of its b) LUMO+1, c) LUMO, d) HOMO, f) HOMO-1 orbitals.


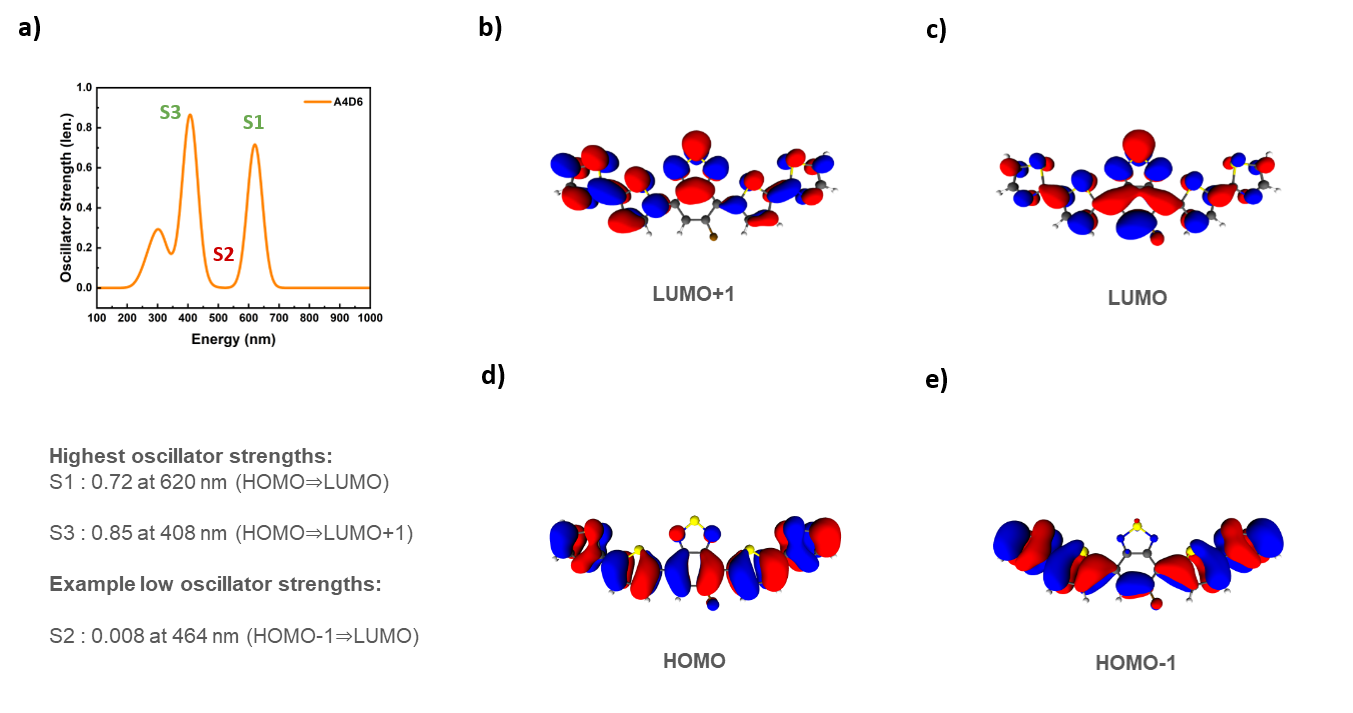


Figure S14. a) Oscillator strength versus transition energy from the ground state to higher states for the computed A4D6 molecule and graphical representation of its b) LUMO+1, c) LUMO, d) HOMO, f) HOMO-1 orbitals.


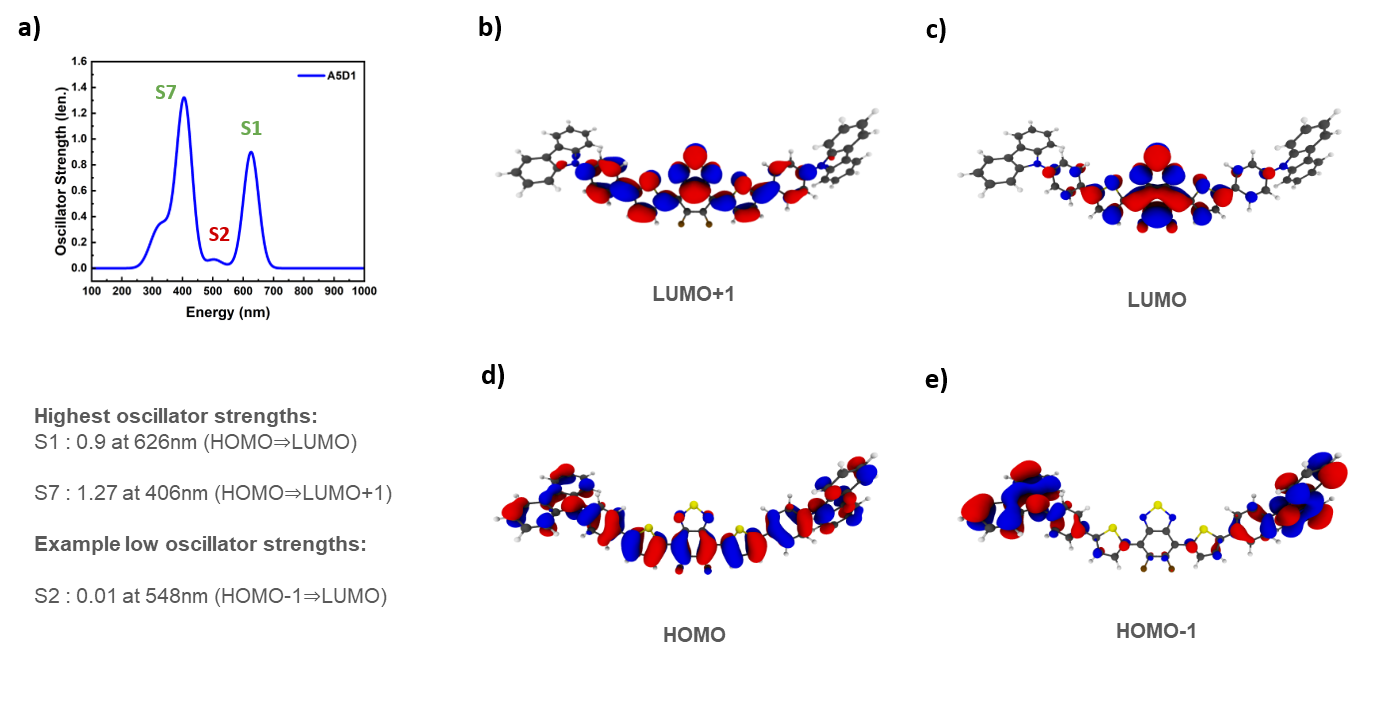


Figure S15. a) Oscillator strength versus transition energy from the ground state to higher states for the computed A5D1 molecule and graphical representation of b) LUMO+1, c) LUMO, d) HOMO, f) HOMO-1 orbitals.


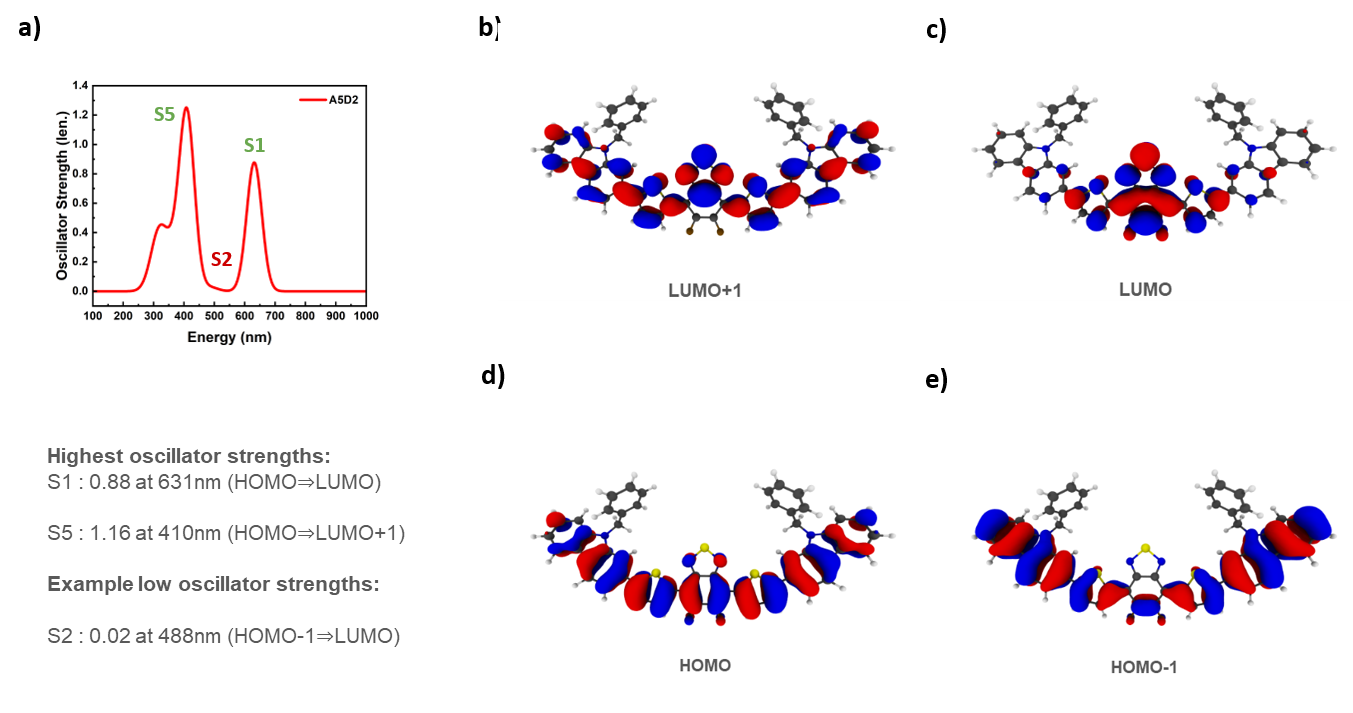


Figure S16. a) Oscillator strength versus transition energy from the ground state to higher states for the computed A5D2 molecule and graphical representation of its b) LUMO+1, c) LUMO, d) HOMO, f) HOMO-1 orbitals.


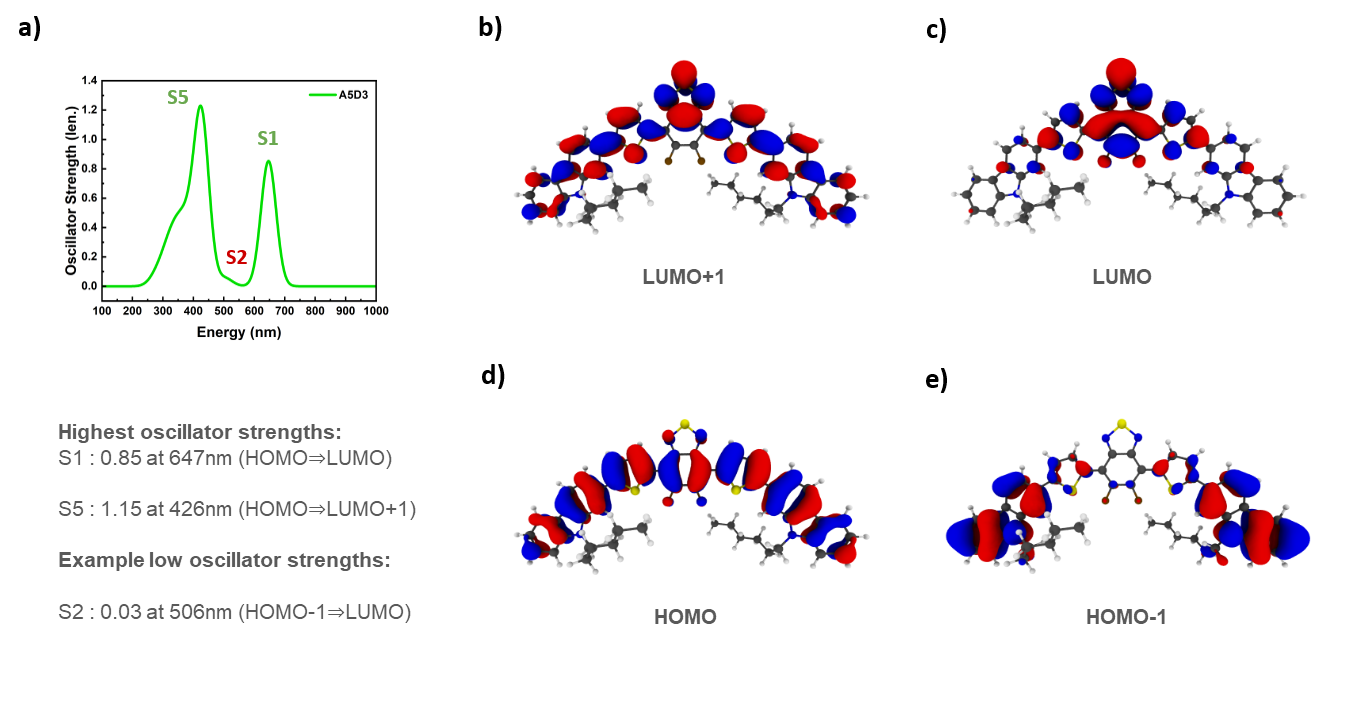


Figure S17. a) Oscillator strength versus transition energy from the ground state to higher states for the computed A5D3 molecule and graphical representation of its b) LUMO+1, c) LUMO, d) HOMO, f) HOMO-1 orbitals.


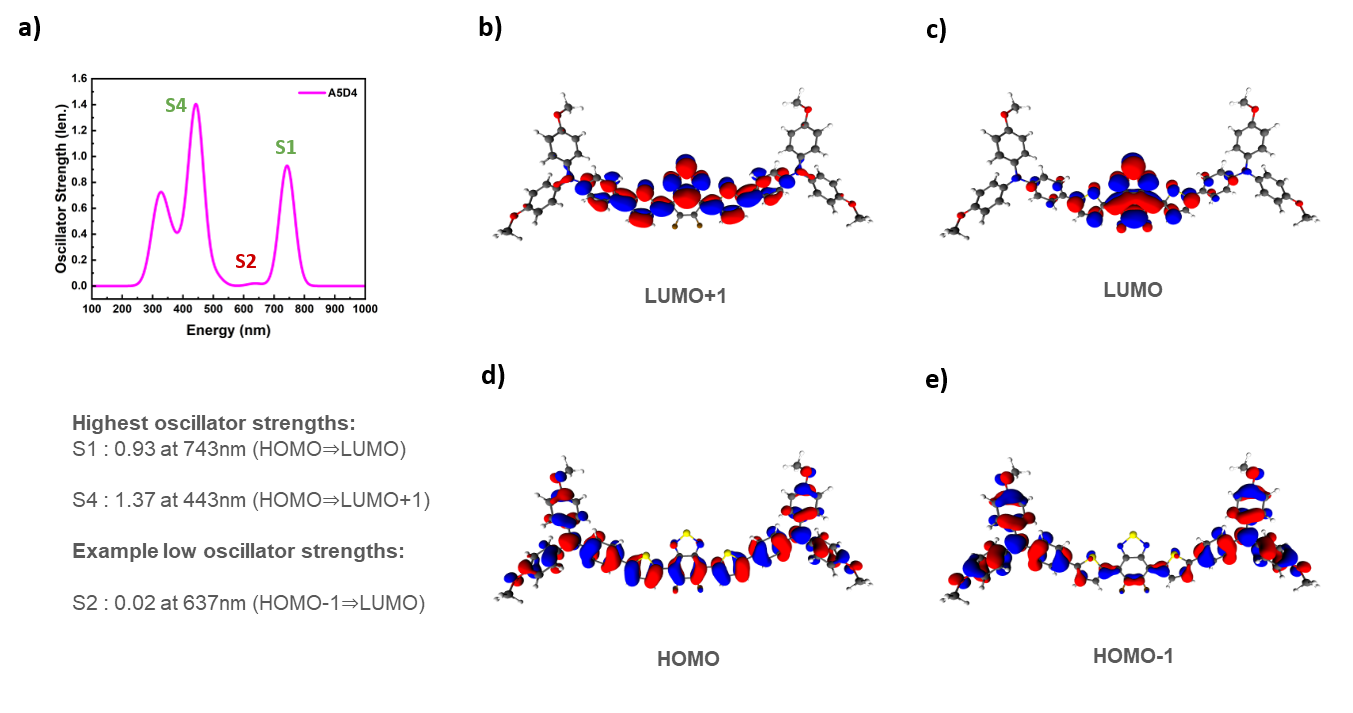
*Figure S18. a) Oscillator strength versus transition energy from the ground state to higher states for the computed A5D4 molecule and graphical representation of its b) LUMO+1, c) LUMO, d) HOMO, f) HOMO-1 orbitals.*


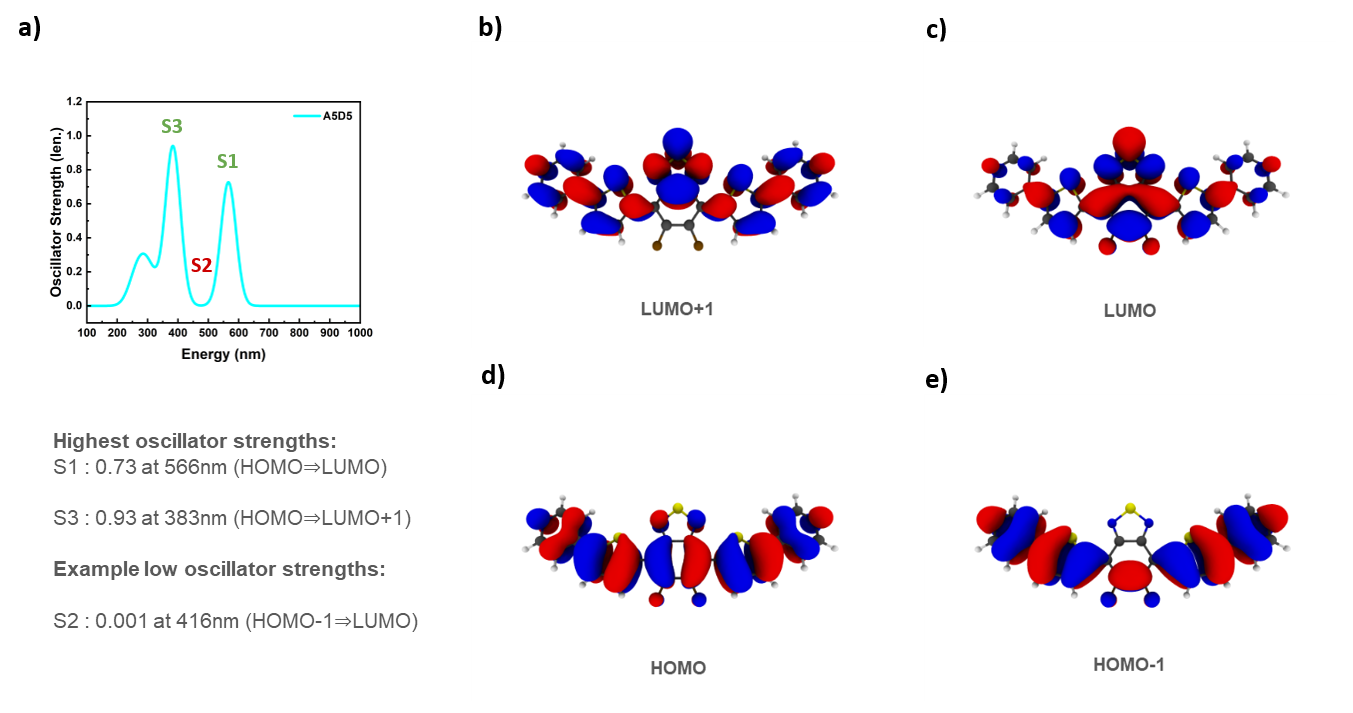


Figure S19. a) Oscillator strength versus transition energy from the ground state to higher states for the computed A5D5 molecule and graphical representation of its b) LUMO+1, c) LUMO, d) HOMO, f) HOMO-1 orbitals.


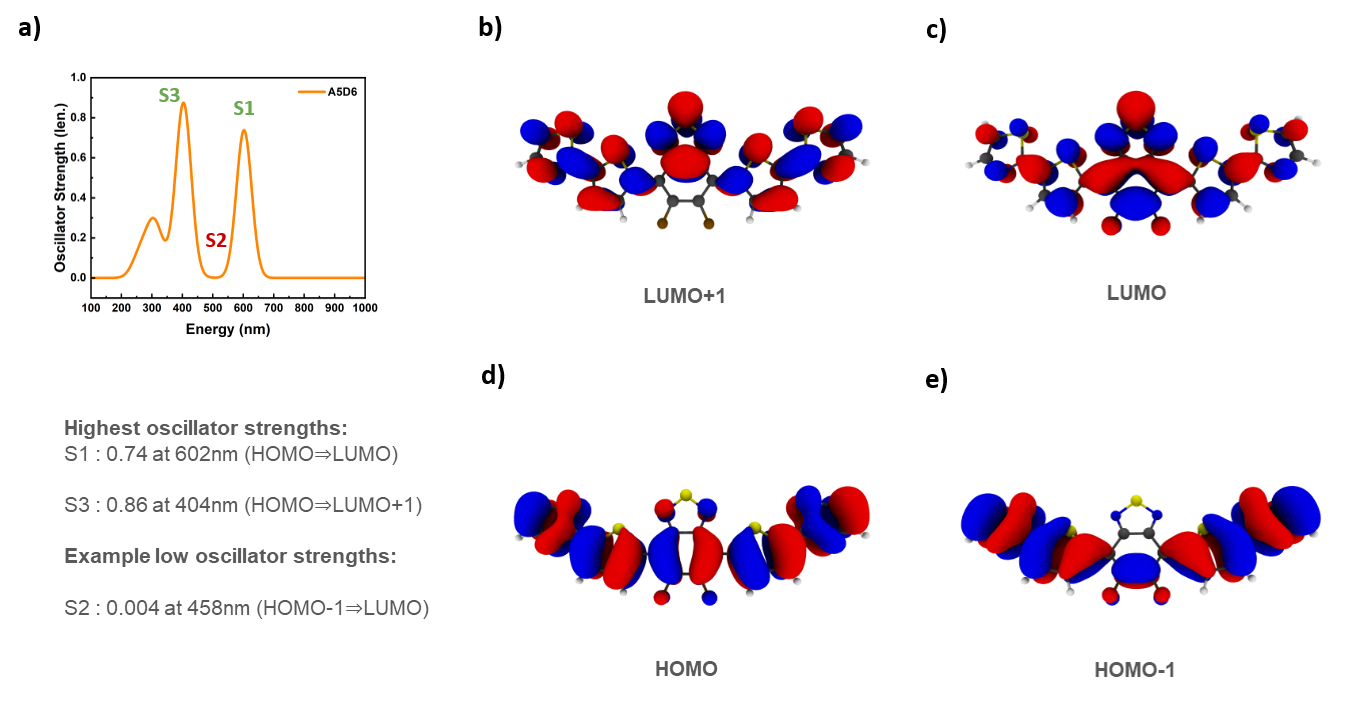


Figure S20. a) Oscillator strength versus transition energy from the ground state to higher states for the computed A5D6 molecule and graphical representation of its b) LUMO+1, c) LUMO, d) HOMO, f) HOMO-1 orbitals.


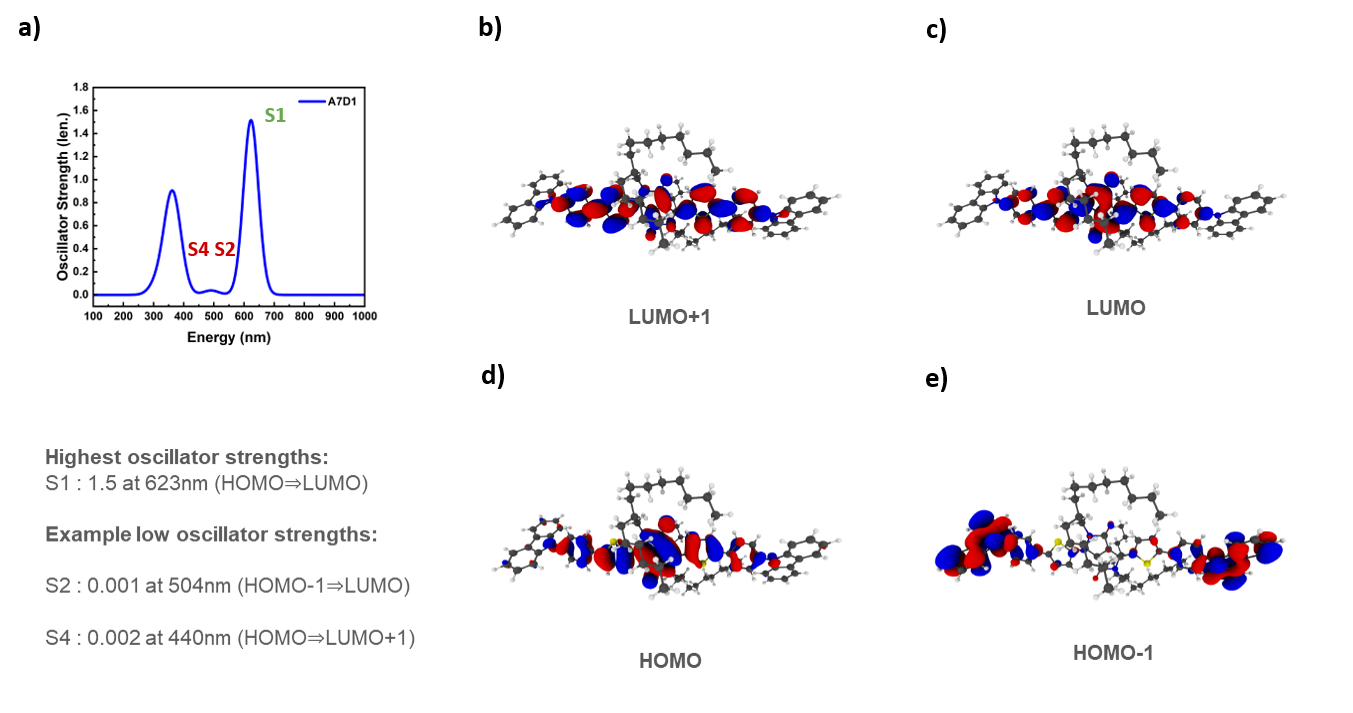


Figure S21. a) Oscillator strength versus transition energy from the ground state to higher states for the computed A7D1 molecule and graphical representation of its b) LUMO+1, c) LUMO, d) HOMO, f) HOMO-1 orbitals.


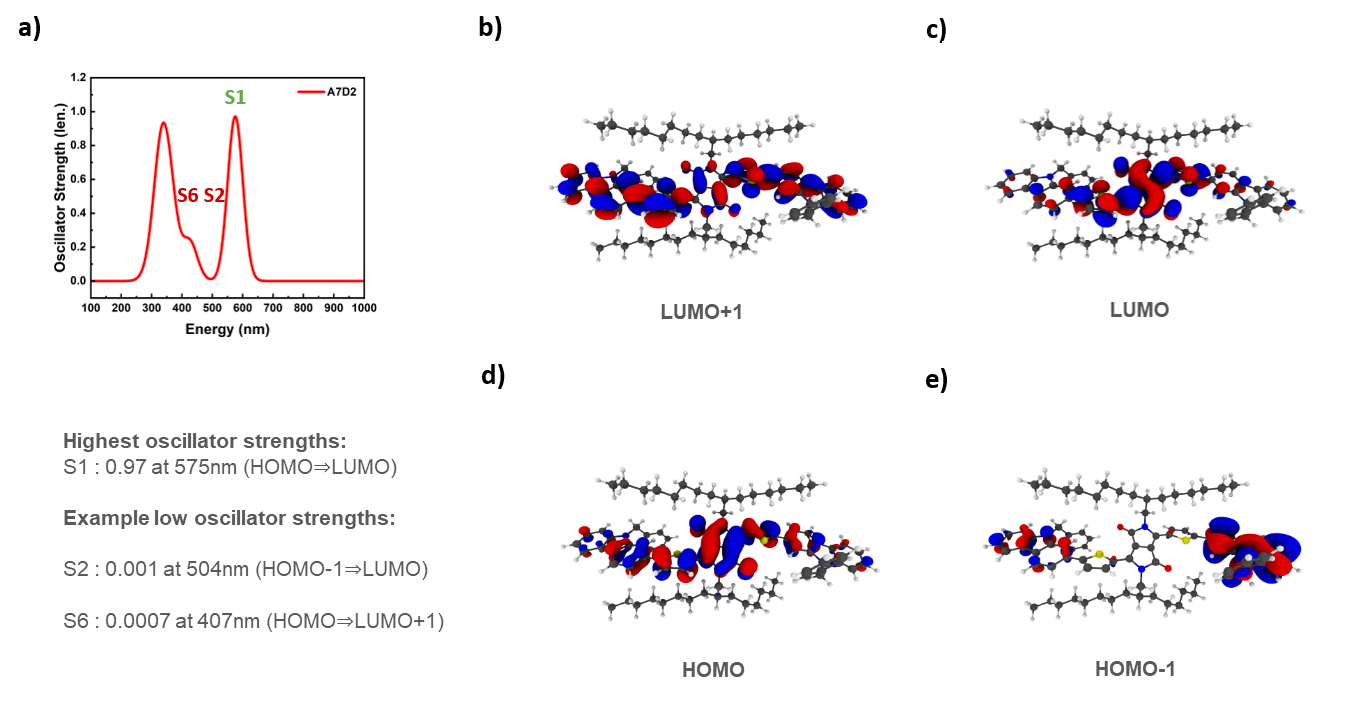


Figure S22. a) Oscillator strength versus transition energy from the ground state to higher states for the computed A7D2 molecule and graphical representation of its b) LUMO+1, c) LUMO, d) HOMO, f) HOMO-1 orbitals.


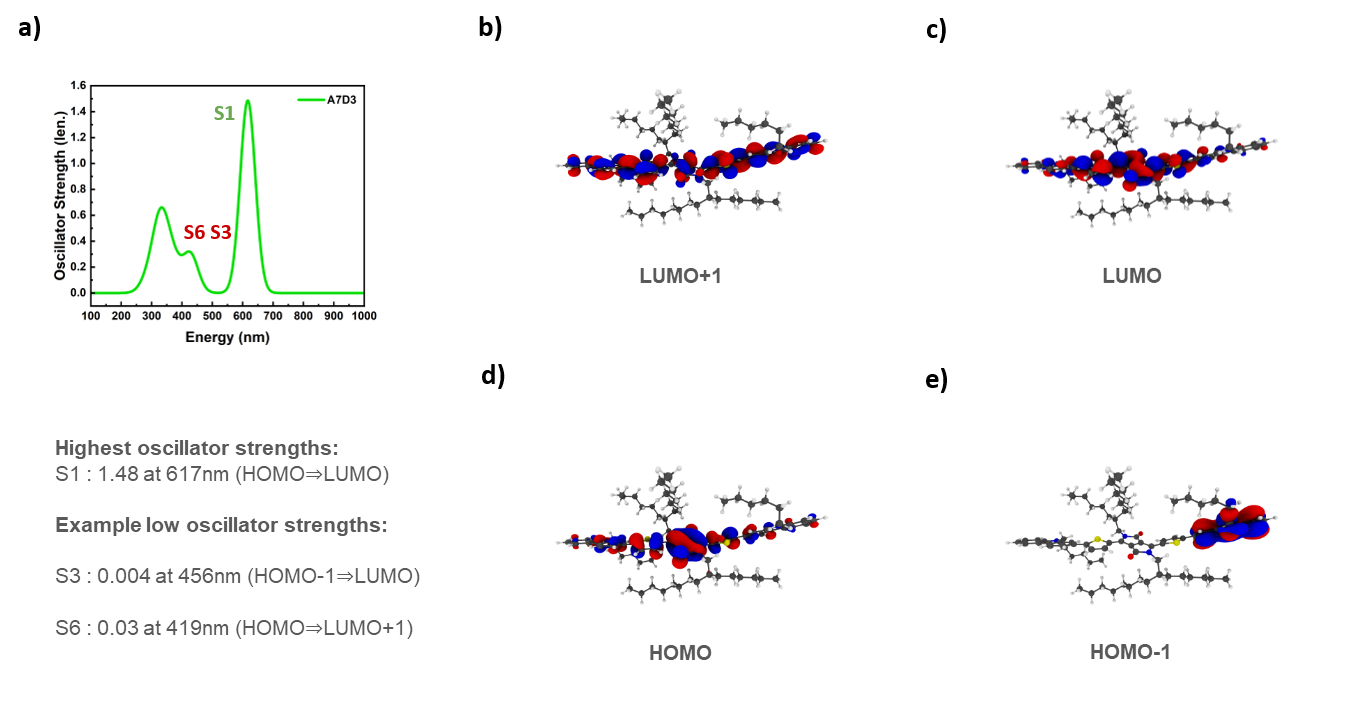


Figure S23. a) Oscillator strength versus transition energy from the ground state to higher states for the computed A7D3 molecule and graphical representation of its b) LUMO+1, c) LUMO, d) HOMO, f) HOMO-1 orbitals.


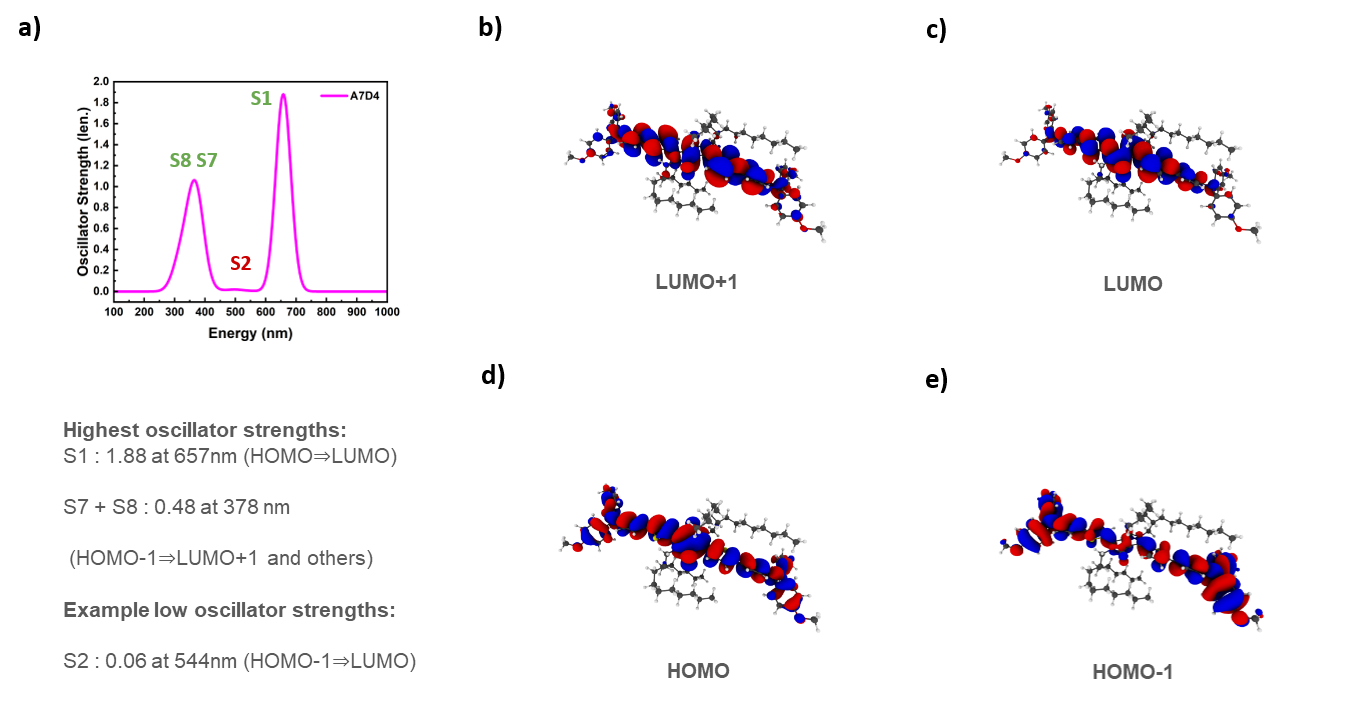


Figure S24. a) Oscillator strength versus transition energy from the ground state to higher states for the computed A7D4 molecule and graphical representation of its b) LUMO+1, c) LUMO, d) HOMO, f) HOMO-1 orbitals.


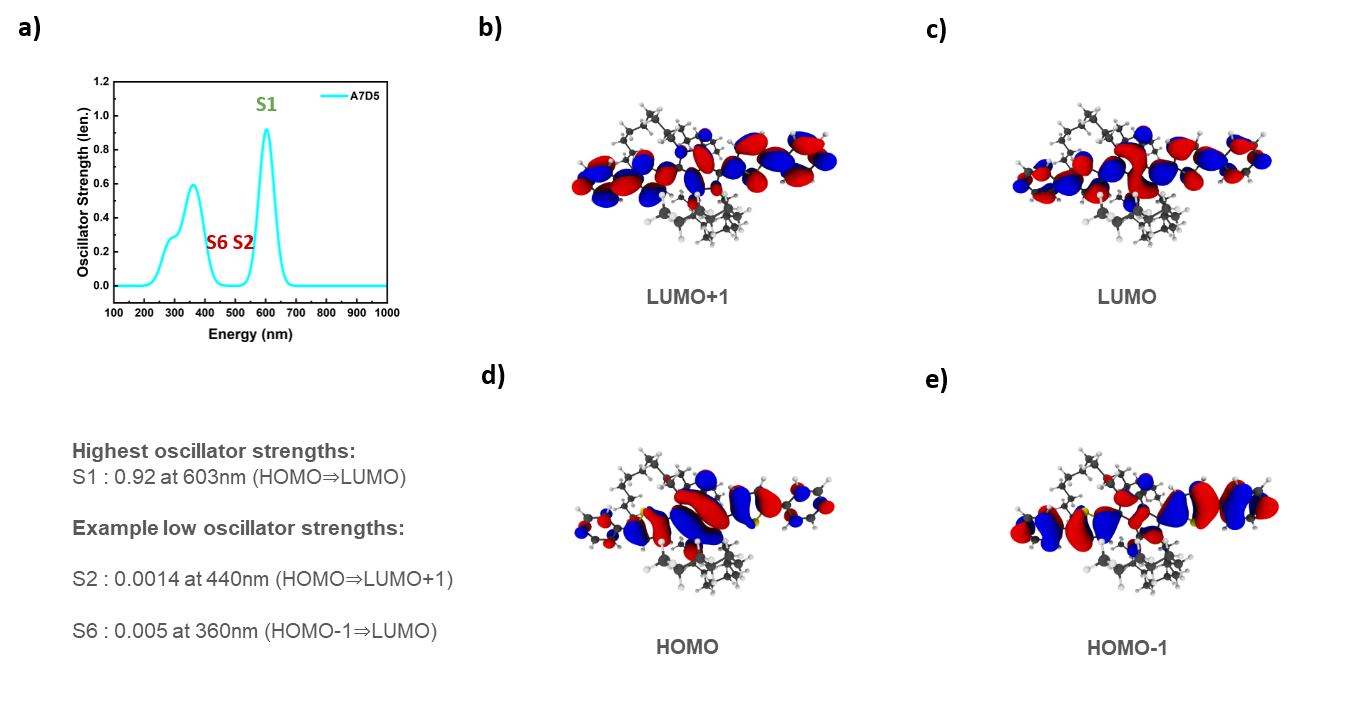
*Figure S25. a) Oscillator strength versus transition energy from the ground state to higher states for the computed A7D5 molecule and graphical representation of its b) LUMO+1, c) LUMO, d) HOMO, f) HOMO-1 orbitals.*


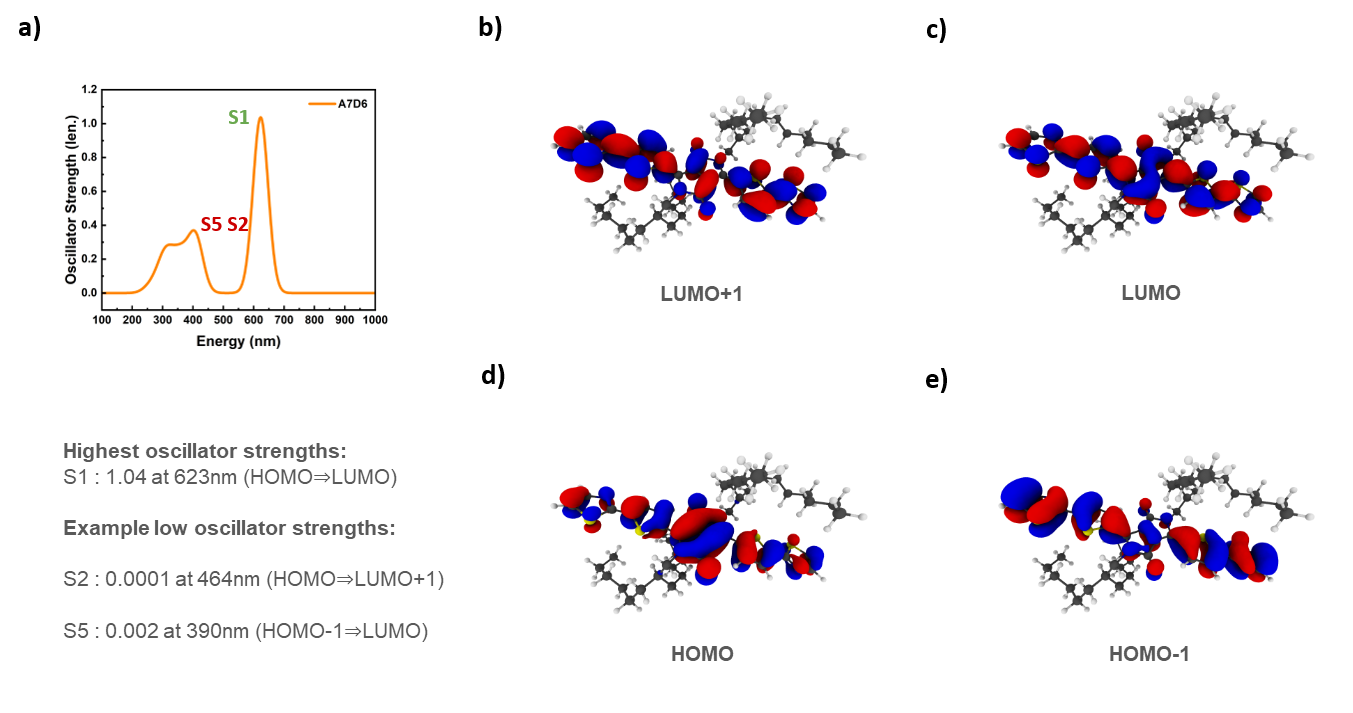


Figure S26. a) Oscillator strength versus transition energy from the ground state to higher states for the computed A7D6 molecule and graphical representation of its b) LUMO+1, c) LUMO, d) HOMO, f) HOMO-1 orbitals.

1. **Electrochemical Characterizations**

Figure S27. Cyclic voltammograms, oxidation and reduction potentials, LUMO and HOMO energy levels for a) A2D1 b) A2D2 c) A2D3 d) A2D4 e) A2D5 and f) A2D6.

Figure S28. Cyclic voltammograms, oxidation and reduction potentials, LUMO and HOMO energy levels for a) A4D1 b) A4D2 c) A4D3 d) A4D4 e) A4D5 and f) A4D6.

Figure S29. Cyclic voltammograms, oxidation and reduction potentials, LUMO and HOMO energy levels for a) A5D1 b) A5D2 c) A5D3 d) A5D4 e) A5D5 and f) A5D6.

Figure S30. Cyclic voltammograms, oxidation and reduction potentials, LUMO and HOMO energy levels for a) A7D1 b) A7D2 c) A7D3 d) A7D4 e) A7D5 and f) A7D6.

The HOMO energy level (E_HOMO_), LUMO energy level (E_LUMO_) and the electronic energy gap (E_g_^el^) of small molecules were calculated through following equations:

$$E_{\mathrm{HOMO}}(eV)= -\left( E_{\mathrm{ox}}^{\mathrm{onset}}+4.80 \right)\mathrm{eV}$$

$$E_{\mathrm{LUMO}}\left( \mathrm{eV} \right)= -\left( E_{\mathrm{red}}^{\mathrm{onset}}+4.80 \right)\mathrm{eV}$$

$$E_{g}\left( \mathrm{eV} \right)=E_{\mathrm{LUMO}}(eV)-E_{\mathrm{HOMO}}(eV)$$

Where $E_{\mathrm{ox}}^{\mathrm{onset}}$ is the onset of oxidation potential, $E_{\mathrm{red}}^{\mathrm{onset}}$ is the onset of reduction potential versus the ferrocene standard. The value of standard electrode potential of normal hydrogen electrode (NHE) was considered as −4.8 V with respect to the vacuum level.

| **Table S1.** Electrochemical properties, theoretical and experimental energy levels of the small molecules. | | | | | | | | | |
| --- | --- | --- | --- | --- | --- | --- | --- | --- | --- |
| Small Molecule |  | E_HOMO_ [eV] | E_LUMO_  [eV] |  | E_ox_^onset^  [V] | E_red_^onset^ [V] |  | E_HOMO_ [eV] | E_LUMO_  [eV] |
|  |  | TDDFT | |  | CV | |  | CV | |
| **A2D1** |  | -5,35 | -2,63 |  | 1,04 | -1,24 |  | -5,62 | -3,34 |
| **A2D2** |  | -5,12 | -2,49 |  | 0,54 | -1,54 |  | -5,12 | -3,04 |
| **A2D3** |  | -5,12 | -2,56 |  | 0,70 | -1,50 |  | -5,28 | -3,08 |
| **A2D4** |  | -4,66 | -2,24 |  | 0,36 | -1,45 |  | -4,94 | -3,13 |
| **A2D5** |  | -5,70 | -2,83 |  | 1,01 | -1,26 |  | -5,59 | -3,32 |
| **A2D6** |  | -5,46 | -2,74 |  | 1,01 | -1,21 |  | -5,59 | -3,37 |
| **A4D1** |  | -5,31 | -3,08 |  | 0,86 | -1,17 |  | -5,44 | -3,41 |
| **A4D2** |  | -5,07 | -2,84 |  | 0,63 | -1,27 |  | -5,21 | -3,31 |
| **A4D3** |  | -5,07 | -2,90 |  | 0,82 | -1,16 |  | -5,40 | -3,42 |
| **A4D4** |  | -4,60 | -2,67 |  | 0,31 | -1,31 |  | -4,89 | -3,27 |
| **A4D5** |  | -5,69 | -3,26 |  | 0,93 | -1,09 |  | -5,51 | -3,49 |
| **A4D6** |  | -5,42 | -3,15 |  | 0,84 | -1,17 |  | -5,42 | -3,41 |
| **A5D1** |  | -5,37 | -3,08 |  | 0,86 | -1,18 |  | -5,44 | -3,40 |
| **A5D2** |  | -5,15 | -2,85 |  | 0,59 | -1,11 |  | -5,17 | -3,47 |
| **A5D3** |  | -5,15 | -2,89 |  | 0,67 | -1,34 |  | -5,25 | -3,24 |
| **A5D4** |  | -4,65 | -2,70 |  | 0,37 | -1,41 |  | -4,95 | -3,17 |
| **A5D5** |  | -5,78 | -3,28 |  | 1,03 | -1,12 |  | -5,61 | -3,46 |
| **A5D6** |  | -5,51 | -3,17 |  | 1,08 | -1,19 |  | -5,66 | -3,39 |
| **A7D1** |  | -5,07 | -2,96 |  | 0,63 | -1,22 |  | -5,21 | -3,36 |
| **A7D2** |  | -5,06 | -2,69 |  | 0,26 | -1,50 |  | -4,84 | -3,08 |
| **A7D3** |  | -4,88 | -2,73 |  | 0,48 | -1,36 |  | -5,06 | -3,22 |
| **A7D4** |  | -4,55 | -2,51 |  | 0,30 | -1,41 |  | -4,88 | -3,17 |
| **A7D5** |  | -5,35 | -3,20 |  | 0,70 | -1,18 |  | -5,28 | -3,40 |
| **A7D6** |  | -5,15 | -3,08 |  | 0,87 | -1,07 |  | -5,45 | -3,51 |

Figure S31. Comparison of experimental and theoretical (TDDFT) a) HOMO, b) LUMO energy levels.

1. **Optical Characterization**

**
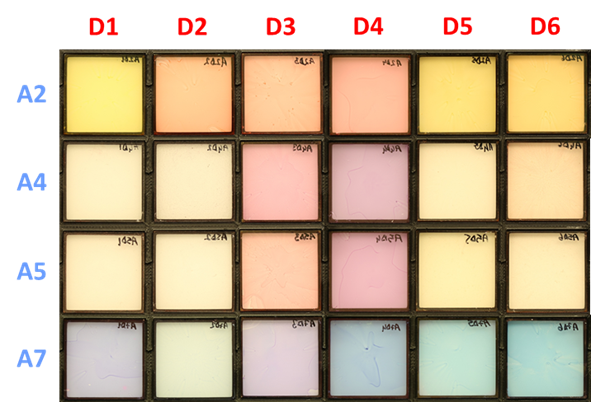
**

Figure S32. Films of synthesized twenty-four small molecules prepared via spin coating using 8 mg/mL solution in chloroform on glass substrate for solid state characterization.

Figure S33. Normalized absorption spectra of small molecules with a) A2 unit, b) A4 unit, c) A5 unit, d) A7 unit as 0.1 mg/mL solution in chlorobenzene, e) A2 unit, f) A4 unit, g) A5, h) A7 unit as film on glass substrates.

Figure S34. Normalized photoluminescence (PL) spectra of small molecules with a) A2 unit, b) A4 unit, c) A5 unit, d) A7 unit as 0.1 mg/mL solution in chlorobenzene, e) A2 unit, f) A4 unit, g) A5 unit, h) A7 unit as film on glass substrates.

| **Table S2.** Optical properties of the small molecules and energy levels. | | | | | | | | | | |
| --- | --- | --- | --- | --- | --- | --- | --- | --- | --- | --- |
| Small Molecule |  | λ _max, abs._ [nm] | |  | λ _max, em._ [nm] | |  | E_g_ [eV] | | |
|  |  | Solution | Film |  | Solution | Film |  | TDDFT | Electronic | Optical |
| **A2D1** |  | 445,47 | 452,53 |  | 564,23 | 776,57 |  | 2,71 | 2,28 | 2,24 |
| **A2D2** |  | 501,04 | 507,25 |  | 594,35 | 642,69 |  | 2,64 | 2,08 | 2,07 |
| **A2D3** |  | 519,69 | 519,02 |  | 586,80 | 644,83 |  | 2,56 | 2,20 | 2,03 |
| **A2D4** |  | 519,7 | 520,52 |  | 648,44 | 679,30 |  | 2,42 | 1,81 | 1,95 |
| **A2D5** |  | 466,35 | 466,62 |  | 562,83 | 717,47 |  | 2,88 | 2,27 | 2,20 |
| **A2D6** |  | 480,1 | 485,09 |  | 569,40 | 769,59 |  | 2,72 | 2,22 | 2,16 |
| **A4D1** |  | 502,25 | 495,89 |  | 636,37 | 766,06 |  | 2,24 | 2,03 | 2,11 |
| **A4D2** |  | 533,86 | 539,95 |  | 701,30 | 733,80 |  | 2,23 | 1,90 | 1,85 |
| **A4D3** |  | 539,4 | 547,74 |  | 669,86 | 725,43 |  | 2,16 | 1,98 | 1,90 |
| **A4D4** |  | 561,35 | 564,71 |  | 772,54 | 782,72 |  | 1,93 | 1,62 | 1,81 |
| **A4D5** |  | 490,36 | 488,92 |  | 586,63 | 724,36 |  | 2,43 | 2,02 | 2,10 |
| **A4D6** |  | 490,36 | 485,09 |  | 602,31 | 777,14 |  | 2,27 | 2,01 | 1,99 |
| **A5D1** |  | 488,3 | 470,86 |  | 620,70 | 715,09 |  | 2,29 | 2,04 | 2,17 |
| **A5D2** |  | 537,96 | 536,39 |  | 675,28 | 719,27 |  | 2,30 | 1,70 | 1,80 |
| **A5D3** |  | 520,73 | 531,05 |  | 638,67 | 675,20 |  | 2,25 | 2,01 | 1,94 |
| **A5D4** |  | 551,71 | 554,72 |  | 770,82 | 776,98 |  | 1,95 | 1,78 | 1,82 |
| **A5D5** |  | 472,71 | 469,36 |  | 571,53 | 712,46 |  | 2,50 | 2,14 | 2,16 |
| **A5D6** |  | 485,63 | 483,86 |  | 586,22 | 757,03 |  | 2,34 | 2,27 | 2,07 |
| **A7D1** |  | 581,26 | 560,19 |  | 646,47 | 815,14 |  | 2,11 | 1,85 | 1,78 |
| **A7D2** |  | 575,92 | 681,67 |  | 657,72 | 803,32 |  | 2,37 | 1,76 | 1,72 |
| **A7D3** |  | 565,46 | 579,07 |  | 655,91 | 801,19 |  | 2,16 | 1,84 | 1,73 |
| **A7D4** |  | 593,78 | 576,06 |  | 692,02 | 817,69 |  | 2,04 | 1,71 | 1,68 |
| **A7D5** |  | 668,26 | 622,85 |  | 635,23 | 701,55 |  | 2,15 | 1,88 | 1,73 |
| **A7D6** |  | 651,03 | 650,75 |  | 650,98 | 748,99 |  | 2,06 | 1,94 | 1,80 |


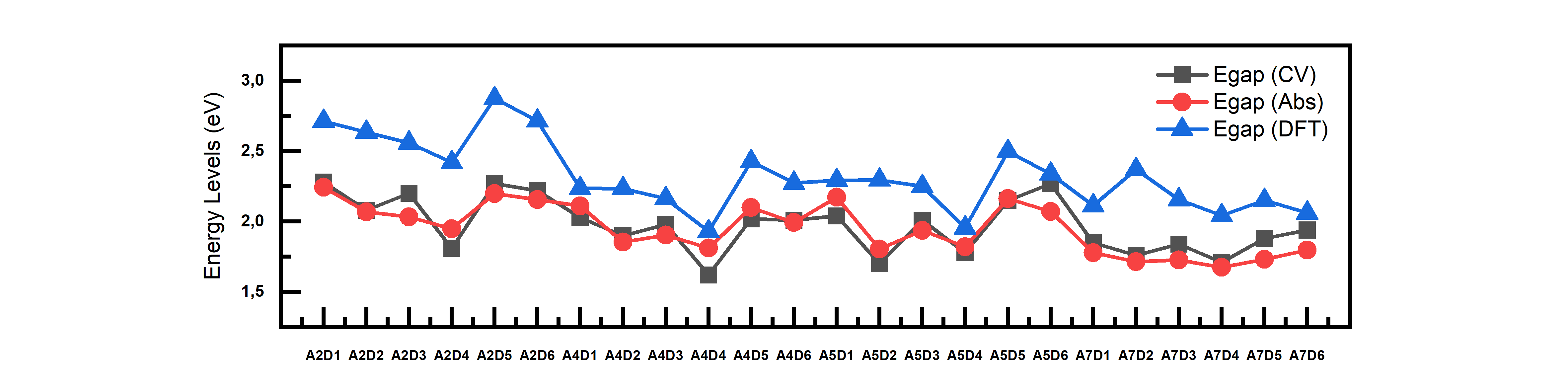


Figure S35. Comparison of electronic, optical and theoretical energy gaps of the small molecules.

| **Table S3.** Excited state lifetimes of the small molecules. | | | | |
| --- | --- | --- | --- | --- |
| Small Molecule |  | τ_1_ [ns] |  | A_1_ |
| **A2D1** |  | 0,34019 ± 0,00320 |  | 1,15050 ± 0,00708 |
| **A2D2** |  | 0,46969 ± 0,00247 |  | 1,11191 ± 0,00376 |
| **A2D3** |  | 0,51728 ± 0,00458 |  | 1,04820 ± 0,00525 |
| **A2D4** |  | 0,47076 ± 0,00223 |  | 1,16251 ± 0,00379 |
| **A2D5** |  | 0,40171 ± 0,00402 |  | 1,04757 ± 0,00612 |
| **A2D6** |  | 0,53 ± 0,00594 |  | 1,01503 ± 0,00615 |
| **A4D1** |  | 5,47646 ± 0,00261 |  | 0,97435 ± 3,06819E-4 |
| **A4D2** |  | 4,72202 ± 0,00289 |  | 0,96053 ± 3,88461E-4 |
| **A4D3** |  | 4,56003 ± 7,14357E-4 |  | 1,00547 ± 1,10076E-4 |
| **A4D4** |  | 3,51355 ± 0,00184 |  | 0,94375 ± 3,10765E-4 |
| **A4D5** |  | 6,09998 ± 0,00324 |  | 1,00956 ± 3,46031E-4 |
| **A4D6** |  | 4,32175 ± 9,05954E-4 |  | 0,95721 ± 1,31307E-4 |
| **A5D1** |  | 4,77031 ± 0,00269 |  | 1,01880 ± 3,94879E-4 |
| **A5D2** |  | 3,89503 ± 0,00701 |  | 0,92711 ± 0,00112 |
| **A5D3** |  | 4,27338 ± 7,19605E-4 |  | 0,9457 ± 1,04086E-4 |
| **A5D4** |  | 3,43896 ± 0,00182 |  | 1,02329± 3,67803E-4 |
| **A5D5** |  | 5,15218 ± 0,0028 |  | 1,02332 ± 3,74979E-4 |
| **A5D6** |  | 3,27654 ± 7,25876E-4 |  | 0,98385 ± 1,463E-4 |
| **A7D1** |  | 3,86349 ± 0,00246 |  | 1,05444± 4,64386E-4 |
| **A7D2** |  | 2,97919 ± 5,90195E-4 |  | 1,11995 ± 1,69419E-4 |
| **A7D3** |  | 2,94957 ± 5,75821E-4 |  | 0,94066 ± 1,17699E-4 |
| **A7D4** |  | 3,25217 ± 0,00347 |  | 1,00765 ± 7,01688E-4 |
| **A7D5** |  | 4,30217 ± 0,00150 |  | 1,00366 ± 2,37322E-4 |
| **A7D6** |  | 3,72019 ± 7,65794E-4 |  | 0,97983 ± 1,34666E-4 |

1. **Synthesis and Structural Characterizations**

*Synthesis of A2D1:* 1,3-Bis(5-bromo-2-thienyl)-5,7-bis(2-ethylhexyl)-4H,8H-benzo[1,2-c:4,5-c′]dithiophene-4,8-dione (100.0 mg, 0.1 mmol), 9H-carbazole-9-(4-phenyl) boronic acid pinacol ester (120.0 mg, 0.3 mmol), tetrakis(-triphenylphosphine) palladium (0) (15.0 mg, 13.0 µmol), K_2_CO_3_ (0.4 mL, 2.5 M) and aliquat 336 (1-2 drops) were taken into a 10-mL pyrex vessel. Mixture was dissolved by 3 mL of toluene and the cap of the vessel was sealed in a glove box under an inert atmosphere. The reaction mixture was stirred and purged with nitrogen at room temperature for 15 minutes. Subsequently, the mixture was irradiated at 110 °C for 1 hour in a microwave reactor, with the initial power set at 150 W. Upon completion of the reaction, the organic phase was separated from the aqueous phase through decantation, and the organic layer was concentrated using a sample concentrator. The crude product was purified by column chromatography on silica gel within 20 mL syringe cartridges, utilizing a vacuum manifold and a gradient solvent system of hexane and chloroform. The final product was obtained as a yellow solid and its structure and composition were supported by the subsequent characterization data. (89.8 mg, 63.3 %) ^1^H NMR (400 MHz, CDCl_3_, δ): 8.18 – 8.12 (m, 4H), 7.93 – 7.81 (m, 4H), 7.66 – 7.58 (m, 4H), 7.49 – 7.41 (m, 10H), 7.34 – 7.29 (m, 4H), 7.08 (dd, J = 8.3, 4.1 Hz, 2H), 3.32 (d, J = 7.5 Hz, 4H), 1.80 – 1.76 (m, 2H), 1.38 – 1.30 (m, 16H), 0.93 – 0.89 (m, 12H). MALDI-TOF-MS (m/z): [M + H]^+^ calcd. for C_70_H_62_N_2_O_2_S_4_, 1091.5; found, 1090.4. Anal. calcd. for C_70_H_62_N_2_O_2_S_4_: C, 77.03; H, 5.73; N, 2.57; 11.75; found: C, 64.54; H, 5.40; N, 1.15; S, 11.79.

*Synthesis of A2D2:* 1,3-Bis(5-bromo-2-thienyl)-5,7-bis(2-ethylhexyl)-4H,8H-benzo[1,2-c:4,5-c′]dithiophene-4,8-dione (60.0 mg, 78.3 µmol), 9-benzyl-9H-carbazole-3-boronic acid pinacol ester (75.0 mg, 0.2 mmol), tetrakis(-triphenylphosphine) palladium (0) (9.0 mg, 7.8 µmol), K_2_CO_3_ (0.3 mL, 2.5 M) and aliquat 336 (1-2 drops) were mixed in a 10-mL pyrex vessel. The mixture was dissolved by adding 3 mL of toluene, and the vessel was sealed in an inert atmosphere. The reaction mixture was stirred and purged with nitrogen at room temperature for 15 minutes, followed by irradiation at 110 °C for 1 hour in a microwave reactor while maintaining the initial power at 150 W. Once the reaction was complete, the organic phase was separated from the aqueous phase by decantation, and the organic layer was concentrated using a sample concentrator. The crude product was purified through silica gel column chromatography using 20 mL syringe cartridges and a vacuum manifold with a gradient solvent system of hexane/chloroform. The final pure product was attained as a yellow-orange solid. (72.7 mg, 83.3 %) ^1^H NMR (400 MHz, CDCl_3_, δ): 8.43 (d, J = 2.1 Hz, 2H), 8.19 (d, J = 7.6 Hz, 2H), 7.86 (d, J = 4.0 Hz, 2H), 7.77 (dd, J = 8.5, 1.9 Hz, 2H), 7.49 – 7.43 (m, 2H), 7.40 – 7.35 (m, 6H), 7.29 (s, 8H), 7.18 – 7.14 (m, 4H), 5.53 (s, 4H), 3.37 (dd, J = 7.1, 3.6 Hz, 4H), 1.82 (t, J = 5.9 Hz, 2H), 1.47 – 1.30 (m, 16H), 0.99 – 0.87 (m, 12H). MALDI-TOF-MS (m/z): [M + H]^+^ calcd. for C_72_H_66_N_2_O_2_S_4_, 1119.6; found, 1120.3. Anal. calcd. for C_72_H_66_N_2_O_2_S_4_: C, 77.24; H, 5.94; N, 2.50; S, 11.45; found: C, 78.41; H, 5.99; N, 2.46; S, 11.59.

*Synthesis of A2D3:* 1,3-Bis(5-bromo-2-thienyl)-5,7-bis(2-ethylhexyl)-4H,8H-benzo[1,2-c:4,5-c′]dithiophene-4,8-dione (50.0 mg, 65.2 µmol), 9-hexyl-2-(4,4,5,5-tetramethyl-1,3,2-dioxaborolan-2-yl)-9H-carbazole (61.5 mg, 0.2 mmol), tetrakis(-triphenylphosphine) palladium (0) (3.8 mg, 3.3 µmol), K_2_CO_3_ (0.4 mL, 2.5 M) and aliquat 336 (1-2 drops) were combined in a 10-mL Pyrex vessel. The mixture was dissolved by adding 3 mL of toluene, and the vessel cap was sealed in an inert atmosphere within a glove box. The reaction mixture was stirred and purged with nitrogen at room temperature for 15 minutes. After that, it was irradiated at 110 °C for 1 hour in a microwave reactor, with the initial power adjusted to 150 W. Once the reaction was complete, the organic phase was separated from the aqueous phase by decantation, and the organic layer was concentrated using a sample concentrator. The crude product was purified by column chromatography on silica gel contained within 20 mL syringe cartridges, utilizing a vacuum manifold with a gradient solvent system of hexane and chloroform. The final purified product was attained as an orange solid, its structure and composition were confirmed by characterization data. (43.6 mg, 60.4 %) ^1^H NMR (400 MHz, CDCl_3_, δ): 8.11 (dd, J = 7.8, 3.8 Hz, 4H), 7.88 (d, J = 3.9 Hz, 2H), 7.68 (s, 2H), 7.59 (d, J = 8.1 Hz, 2H), 7.52 – 7.40 (m, 6H), 7.24 (d, J = 7.8 Hz, 2H), 4.36 (t, J = 7.2 Hz, 4H), 3.37 (qd, J = 14.9, 7.1 Hz, 4H), 1.91 (q, J = 7.3 Hz, 4H), 1.81 (d, J = 5.9 Hz, 2H), 1.46 – 1.28 (m, 28H), 0.97 (t, J = 7.4 Hz, 6H), 0.88 (s, 12H). MALDI-TOF-MS (m/z): [M + H]^+^ calcd. for C_70_H_78_N_2_O_2_S_4_, 1107.7; found, 1106.5. Anal. calcd. for C_70_H_78_N_2_O_2_S_4_: C, 75.91; H, 7.10; N, 2.53; S, 11.58; found: C, 74.33; H, 6.47; N, 2.46; S, 11.46.

*Synthesis of A2D4:* 1,3-Bis(5-bromo-2-thienyl)-5,7-bis(2-ethylhexyl)-4H,8H-benzo[1,2-c:4,5-c′]dithiophene-4,8-dione (60.0 mg, 78.3 µmol), [4-[bis(4-methoxyphenyl)amino]phenyl]boronic acid (68.3 mg, 0.2 mmol), tetrakis(-triphenylphosphine) palladium (0) (9.0 mg, 7.8 µmol), K_2_CO_3_ (0.5 mL, 2.5 M) and aliquat 336 (1-2 drops) were added into a 10-mL pyrex vessel. The blend was dissolved by adding 3 mL of toluene, and the vessel was sealed within a glove box under an inert atmosphere. The reaction mixture was stirred and purged with nitrogen for 15 minutes at room temperature. Following this, the mixture was irradiated at 110 °C for 1 hour in a microwave reactor, with the initial power set at 150 W. Once the reaction was complete, the organic phase was decanted to separate it from the aqueous phase, and the organic layer was concentrated using a sample concentrator. The crude product was then purified using column chromatography on silica gel packed in 20 mL syringe cartridges with a vacuum manifold, employing a gradient solvent system of hexane and chloroform. The pure product was obtained as light orange solid. (93.7 mg, 98.8 %) ^1^H NMR (400 MHz, CDCl_3_, δ): 7.78 (d, J = 4.0 Hz, 2H), 7.48 (d, J = 8.9 Hz, 4H), 7.19 (d, J = 4.0 Hz, 2H), 7.09 (d, J = 9.0 Hz, 8H), 6.93 (d, J = 8.9 Hz, 4H), 6.88 – 6.82 (m, 8H), 3.81 (s, 12H), 3.33 (d, J = 7.0 Hz, 4H), 1.78 (t, J = 5.9 Hz, 2H), 1.41 – 1.28 (m, 16H), 0.95 – 0.86 (m, 12H). MALDI-TOF-MS (m/z): [M + H]^+^ calcd. for C_74_H_74_N_2_O_6_S_4_, 1215.7; found, 1215.4. Anal. calcd. for C_74_H_74_N_2_O_6_S_4_: C, 73.11; H, 6.14; N, 2.30; S, 10.55; found: C, 72.95; H, 6.09; N, 2.16; S, 10.54.

*Synthesis of A2D5:* 1,3-Bis(5-bromo-2-thienyl)-5,7-bis(2-ethylhexyl)-4H,8H-benzo[1,2-c:4,5-c′]dithiophene-4,8-dione (80.0 mg, 0.2 mmol), 4-pyridinylboronic acid (102.5 mg, 0.4 mmol), tetrakis(-triphenylphosphine) palladium (0) (20.0 mg, 17.0 µmol), K_2_CO_3_ (0.7 mL, 2.5 M) and aliquat 336 (1-2 drops) were taken into a 10-mL pyrex vessel. The mixture was dissolved by adding 3 mL of toluene, and the vessel's cap was sealed in a glove box under an inert atmosphere. The reaction mixture was stirred and purged with nitrogen at room temperature for 15 minutes. Subsequently, it was irradiated at 110 °C for 1 hour using a microwave reactor, maintaining the initial power at 150 W. Upon completion of the reaction, the organic phase was separated from the aqueous phase via decantation, and the organic phase was concentrated using a sample concentrator. The crude product was purified through column chromatography on silica gel contained in 20 mL syringe cartridges, using a vacuum manifold with a gradient solvent system of hexane and chloroform. The final product was acquired as a dark yellow solid and the results confirm the structure of the molecules and their composition. (54.4 mg, 41.9 %) ^1^H NMR (400 MHz, CDCl_3_, δ): 8.64 (dd, J = 4.6, 1.5 Hz, 4H), 7.77 (d, J = 4.0 Hz, 2H), 7.55 (dd, J = 4.6, 1.5 Hz, 4H), 7.51 (d, J = 4.0 Hz, 2H), 3.33 (d, J = 6.9 Hz, 4H), 1.81 – 1.75 (m, 2H), 1.42 – 1.31 (m, 16H), 0.94 – 0.87 (m, 12H). MALDI-TOF-MS (m/z): [M + H]^+^ calcd. for C_44_H_46_N_2_O_2_S_4_, 763.1; found, 762.2. Anal. calcd. for C_44_H_46_N_2_O_2_S_4_: C, 69.25; H, 6.08; N, 3.67; S, 16.81; found: C, 67.11; H, 5.74; N, 2.99; S, 16.76.

*Synthesis of A2D6:* 1,3-Bis(5-bromo-2-thienyl)-5,7-bis(2-ethylhexyl)-4H,8H-benzo[1,2-c:4,5-c′]dithiophene-4,8-dione (75.0 mg, 0.1 mmol), 2-tributylstannylthiazole (91.5 mg, 0.3 mmol) and tetrakis(-triphenylphosphine) palladium (0) (11.3 mg, 9.8 µmol) were mixed in a 10-mL pyrex vessel. The mixture was dissolved by adding 3 mL of toluene, and the vessel was sealed under an inert atmosphere in a glove box. The reaction mixture was then stirred and purged with nitrogen at room temperature for 15 minutes. Following this, the mixture was irradiated at 110 °C for 1 hour in a microwave reactor, with an initial power set to 150 W. The crude product was purified using column chromatography on silica gel packed in 20 mL syringe cartridges, utilizing a vacuum manifold with a gradient solvent system of hexane and chloroform. The final pure product was collected as a dark yellow solid and characterization data supported the presence of structure and composition. (55.6 mg, 73.1 %) ^1^H NMR (400 MHz, CDCl_3_, δ): 7.83 (d, J = 3.2 Hz, 2H), 7.75 (d, J = 4.0 Hz, 2H), 7.53 (d, J = 4.0 Hz, 2H), 7.32 (d, J = 3.3 Hz, 2H), 3.33 (d, J = 7.4 Hz, 4H), 1.76 (dt, J = 12.3, 5.9 Hz, 2H), 1.45 – 1.27 (m, 16H), 0.95 – 0.86 (m, 12H). MALDI-TOF-MS (m/z): [M + H]^+^ calcd. For C_40_H_42_N_2_O_2_S_6_, 775.15; found, 774.1. Anal. calcd. for C_40_H_42_N_2_O_2_S_6_: C, 61.98; H, 5.46; N, 3.61; S, 24.82; found: C, 57.60; H, 5.00; N, 3.22; S, 23.40.

*Synthesis of A4D1:* 4,7-Bis(5-bromothiophen-2-yl)-5-fluorobenzo[c][1,2,5]thiadiazole (50.0 mg, 0.1 mmol), 9H-carbazole-9-(4-phenyl) boronic acid pinacol ester (96.9 mg, 0.3 mmol), tetrakis(-triphenylphosphine) palladium (0) (12.1 mg, 10.5 µmol), K_2_CO_3_ (0.4 mL, 2.5 M) and aliquat 336 (1-2 drops) were added to a 10-mL pyrex vessel. The mixture was dissolved by introducing 3 mL of toluene, and the vessel was sealed in a glove box under an inert atmosphere. The reaction mixture was stirred and purged with nitrogen at room temperature for 15 minutes. After that, the mixture was irradiated at 110 °C for 1 hour using a microwave reactor, with the initial power set to 150 W. Upon completion of the reaction, the organic phase was separated from the aqueous phase by decantation, and the organic layer was concentrated using a sample concentrator. The product was recrystallized via dissolving in chloroform and precipitation over cold hexane. The final product yielded as a red solid after filtration and washed with cold hexane. Presence of its structure and composition were supported by characterization data. (59.2 mg, 70.4 %) ^1^H NMR (400 MHz, CDCl_3_, δ): 8.18 (d, J = 7.6 Hz, 4H), 7.93 (d, J = 8.6 Hz, 4H), 7.72 (d, J = 8.5 Hz, 4H), 7.56 – 7.41 (m, 11H), 7.40 – 7.27 (m, 6H). MALDI-TOF-MS (m/z): [M + H]+ calcd. for C_50_H_29_FN_4_S_3_, 801.0; found, 800.8. Anal. calcd. for C_50_H_29_FN_4_S_3_: C, 74.98; H, 3.65; N, 6.99; S, 12.04; found: C, 73.74; H, 3.57; N, 6.63; S, 10.79.

*Synthesis of A4D2:* 4,7-Bis(5-bromothiophen-2-yl)-5-fluorobenzo[c][1,2,5]thiadiazole (50.0 mg, 78.3 µmol), 9-benzyl-9H-carbazole-3-boronic acid pinacol ester (100.6 mg, 0.3 mmol), tetrakis(-triphenylphosphine) palladium (0) 12.1 mg, 10.5 µmol), K2CO3 (0.4 mL, 2 M) and aliquat 336 (1-2 drops) were combined in a 10-mL Pyrex vessel. The mixture was dissolved by adding 3 mL of toluene, and the vessel cap was sealed in a glove box under an inert atmosphere. The reaction mixture was stirred and purged with nitrogen for 15 minutes at room temperature. Following this, the mixture was irradiated at 110 °C for 1 hour using a microwave reactor, with the initial power controlled at 150 W. Once the reaction was finished, the organic phase was separated from the aqueous phase by decantation, and the organic layer was concentrated using a sample concentrator. The crude product was purified through recrystallization by dissolving it in chloroform and precipitating it with cold hexane. The final pure product was obtained as a red solid after filtration from hexane. The structure and composition were supported by characterization results. (67.0 mg, 77.0 %) ^1^H NMR (400 MHz, CDCl_3_, δ): 8.46 (d, J = 8.6 Hz, 2H), 8.29 (d, J = 3.9 Hz, 1H), 8.19 (d, J = 12.3 Hz, 2H), 7.88 – 7.72 (m, 6H), 7.52 – 7.31 (m, 14H), 7.15 (d, J = 7.8 Hz, 4H), 5.54 (s, 4H). MALDI-TOF-MS (m/z): [M + H]+ calcd. for C_52_H_33_FN_4_S_3_, 829.0; found, 828.9. Anal. calcd. for C_52_H_33_FN_4_S_3_: C, 75.34; H, 4.01; N, 6.76; S, 11.60; found: C, 73.25; H, 3.56; N, 6.47; S, 11.45.

*Synthesis of A4D3:* 4,7-Bis(5-bromothiophen-2-yl)-5-fluorobenzo[c][1,2,5]thiadiazole (40.0 mg, 0.1 mmol), 9-hexyl-2-(4,4,5,5-tetramethyl-1,3,2-dioxaborolan-2-yl)-9H-carbazole (79.2 mg, 0.2 mmol), tetrakis(-triphenylphosphine) palladium (0) (4.9 mg, 4.2 µmol), K_2_CO_3_ (0.4 mL, 2.5 M) and aliquat 336 (1-2 drops) were placed into a 10-mL Pyrex vessel. The mixture was dissolved by adding 3 mL of toluene, and the vessel cap was sealed under inert conditions in a glove box. The reaction mixture was stirred and purged with nitrogen at room temperature for 15 minutes. After that, the mixture was irradiated at 110 °C for 1 hour in a microwave reactor, with the initial power controlled at 150 W. Once the reaction was complete, the organic phase was separated from the aqueous phase through decantation, and the organic layer was concentrated using a sample concentrator. The crude product was purified via column chromatography on silica gel packed in 20 mL syringe cartridges, utilizing a vacuum manifold with a gradient solvent system consisting of hexane and chloroform. The pure product was acquired as an orange-red solid. (50.0 mg, 73.0 %) ^1^H NMR (400 MHz, CDCl_3_, δ): 8.33 (d, J = 3.8 Hz, 1H), 8.20 (d, J = 3.6 Hz, 1H), 8.15 – 7.99 (m, 5H), 7.89 – 7.37 (m, 12H), 4.37 (t, J = 6.9 Hz, 4H), 1.97 – 1.86 (m, 4H), 1.35 (s, 12H), 0.88 (dt, J = 14.4, 7.1 Hz, 6H). MALDI-TOF-MS (m/z): [M + H]+ calcd. for C_50_H_45_FN_4_S_3_, 817.1; found, 816.3. Anal. calcd. for C_50_H_45_FN_4_S_3_: C, 73.50; H, 5.55; N, 6.86; S, 11.77; found: C, 72.40; H, 5.14; N, 6.62; S, 11.78.

*Synthesis of A4D4:* 4,7-Bis(5-bromothiophen-2-yl)-5-fluorobenzo[c][1,2,5]thiadiazole (50.0 mg, 0.1 mmol), [4-[bis(4-methoxyphenyl)amino]phenyl]boronic acid (91.7 mg, 0.3 mmol), tetrakis(-triphenylphosphine) palladium (0) (12.1 mg, 10.5 µmol), K_2_CO_3_ (0.4, 2.5 M) and aliquat 336 (1-2 drops) were added to a 10-mL pyrex vessel. The mixture was dissolved by adding 3 mL of toluene, and the vessel cap was sealed within a glove box under an inert atmosphere. The reaction mixture was stirred and purged with nitrogen for 15 minutes at room temperature. Following this, the mixture was irradiated at 110 °C for 1 hour with a microwave reactor while maintaining the initial power at 150 W. After the reaction was complete, the organic phase was separated from the aqueous phase by decantation, and the organic layer was concentrated using a sample concentrator. The crude product was purified by column chromatography on silica gel packed in 20 mL syringe cartridges with a vacuum manifold, utilizing a gradient solvent system of hexane and chloroform. The final pure product was obtained as a red-purple solid. (94.7 mg, 97.5 %) ^1^H NMR (400 MHz, CDCl_3_, δ): 8.22 (d, J = 4.0 Hz, 1H), 8.10 (d, J = 3.6 Hz, 1H), 7.73 (d, J = 12.9 Hz, 1H), 7.49 (d, J = 8.4 Hz, 5H), 7.09 (s, 8H), 6.86 (d, J = 9.0 Hz, 13H), 3.82 (s, 12H). ^1^H NMR (400 MHz, CDCl_3_, δ): MALDI-TOF-MS (m/z): [M + H]+ calcd. for C_54_H_41_FN_4_O_4_S_3_, 925.1; found, 924,9. Anal. calcd. for C_54_H_41_FN_4_O_4_S_3_: C, 70.11; H, 4.47; N, 6.06; S, 10.4; found: C, 69.99; H, 4.45; N, 5.69; S, 9.32.

*Synthesis of A4D5:* 4,7-Bis(5-bromothiophen-2-yl)-5-fluorobenzo[c][1,2,5]thiadiazole (60.0 mg, 0.1 mmol), 4-pyridinylboronic acid (38.7 mg, 0.3 mmol), tetrakis(-triphenylphosphine) palladium (0) (14.5 mg, 12.6 µmol), K_2_CO_3_ (0.5 mL, 2.5 M) and aliquat 336 (1-2 drops) were mixed in a 10-mL pyrex vessel. The mixture was dissolved by adding 3 mL of toluene, and the cap of the vessel was sealed in an inert atmosphere within a glove box. The reaction mixture was stirred and purged with nitrogen at room temperature for 15 minutes. Subsequently, the mixture was irradiated at 110 °C for 1 hour in a microwave reactor, with the initial power adjusted to 150 W. After the reaction was completed, the organic phase was separated from the aqueous phase through decantation, and the organic layer was concentrated using a sample concentrator. The product was purified by recrystallization, achieved by dissolving it in chloroform and precipitating over cold hexane. Ultimately, the product was obtained as a yellow solid but couldn’t be purified further due to low solubility. (190 mg) ^1^H NMR (400 MHz, CDCl_3_, δ): 8.66 – 8.61 (m, 2H), 8.39 – 8.27 (m, 4H), 7.95 (d, J = 4.1 Hz, 1H), 7.76 – 7.72 (m, 2H), 7.63 (s, 1H), 7.54 (s, 1H), 7.18 – 7.06 (m, 2H). MALDI-TOF-MS (m/z): [M + H]^+^ calcd. for C_24_H_13_FN_4_S_3_, 472.6; found, 473.6. Anal. calcd. for C_24_H_13_FN_4_S_3_: C, 61.00; H, 2.77; N, 11.86; S, 20.35; found: C, 24.06; H, 1.61; N, 3.45; S, 6.20.

*Synthesis of A4D6:* 4,7-Bis(5-bromothiophen-2-yl)-5-fluorobenzo[c][1,2,5]thiadiazole (60.0 mg, 0.1 mmol), 2-tributylstannylthiazole (117.9 mg, 0.3 mmol) and tetrakis(-triphenylphosphine) palladium (0) (14.6 mg, 12.6 µmol) were taken into a 10-mL pyrex vessel. The mixture was dissolved by adding 3 mL of toluene while inside a glove box under an inert atmosphere, and the cap was securely sealed. The reaction mixture was stirred and purged with nitrogen at room temperature for 15 minutes. Following this, the mixture was irradiated for 1 hour at 110 °C in a microwave reactor, starting with an initial power of 150 W. The crude product was purified by column chromatography on silica gel packed in 20 mL syringe cartridges, utilizing a vacuum manifold with a gradient solvent system of hexane and chloroform. The final pure product was obtained as a yellow-orange solid but couldn’t be purified further due to low solubility. (60.0 mg, 98.9 %) ^1^H NMR (400 MHz, CDCl_3_, δ): 8.29 (d, J = 4.1 Hz, 1H), 8.13 (d, J = 4.0 Hz, 1H), 7.85 – 7.83 (m, 3H), 7.64 (dd, J = 4.1, 1.3 Hz, 1H), 7.61 (d, J = 4.0 Hz, 1H), 7.33 (dd, J = 7.1, 3.2 Hz, 2H). MALDI-TOF-MS (m/z): [M + H]^+^ calcd. For C_20_H_9_FN_4_S_5_, 484.6; found, 483.9. Anal. calcd. for C_20_H_9_FN_4_S_5_: C, 49.57; H, 1.87; N, 11.56; S, 33.08; found: C, 40.12; H, 2.31; N, 7.20; S, 21.20.

*Synthesis of A5D1:* 4,7-Bis(5-bromothiophen-2-yl)-5,6-difluorobenzo[c][1,2,5]thiadiazole (50.0 mg, 0.1 mmol), 9H-carbazole-9-(4-phenyl) boronic acid pinacol ester (93.4 mg, 0.3 mmol), tetrakis(-triphenylphosphine) palladium (0) (11.7 mg, 10.1 µmol), K_2_CO_3_ (0.4 mL, 2.5 M) and aliquat 336 (1-2 dropswere combined in a 10-mL pyrex vessel. The mixture was dissolved by adding 3 mL of toluene, and the vessel was sealed in an inert atmosphere within a glove box. The reaction mixture was stirred and purged with nitrogen for 15 minutes at room temperature. Subsequently, the mixture was irradiated at 110 °C for 1 hour in a microwave reactor, during which the initial power was set to 150 W. Once the reaction was complete, the organic phase was separated from the aqueous phase through decantation, and the organic layer was concentrated using a sample concentrator. The product recrystallized by dissolving it in chloroform and precipitating over cold hexane. The final product was obtained as a yellow solid after filtration and washing with cold hexane. The presence of the expected structure and composition was supported by characterization data. (74.9 mg, 90.5 %) ^1^H NMR (400 MHz, CDCl_3_, δ): 8.15 (d, J = 7.1 Hz, 6H), 7.96 (dd, J = 8.4, 6.1 Hz, 4H), 7.68 – 7.62 (m, 6H), 7.47 – 7.42 (m, 8H), 7.30 (s, 4H). MALDI-TOF-MS (m/z): [M + H]+ calcd. for C_50_H_28_F_2_N_4_S_3_, 819.0; found, 818.6. Anal. calcd. for C_50_H_28_F_2_N_4_S_3_: C, 73.33; H, 3.45; N, 6.84; S, 11.74; found: C, 74.66; H, 3.51; N, 6.74; S, 12.05.

*Synthesis of A5D2:* 4,7-Bis(5-bromothiophen-2-yl)-5,6-difluorobenzo[c][1,2,5]thiadiazole (50.0 mg, 0.1 mmol), 9-benzyl-9H-carbazole-3-boronic acid pinacol ester (97.0 mg, 0.3 mmol), tetrakis(-triphenylphosphine) palladium (0) (11.7 mg, 10.1 µmol), K_2_CO_3_ (0.4 mL, 2.5 M) and aliquat 336 (1-2 drops) were taken into a 10-mL pyrex vessel. The blend was dissolved by adding 3 mL of toluene, and the vessel was sealed in an inert atmosphere within a glove box. The reaction mixture was stirred and purged with nitrogen at room temperature for 15 minutes. Afterwards, the mixture was irradiated at 110 °C for 1 hour in a microwave reactor, adjusting the initial power to 150 W. Upon completion of the reaction, the organic phase was separated from the aqueous phase by decantation, and the organic layer was concentrated using a sample concentrator. The crude product was then recrystallized by dissolving it in chloroform and allowing it to precipitate with cold hexane. The final product was obtained as a yellow solid after filtration through hexane and the presence of the proposed structure and composition by characterization data. (77.6 mg, 90.8 %) ^1^H NMR (400 MHz, CDCl_3_, δ): 8.47 (s, 2H), 8.32 (d, J = 3.9 Hz, 2H), 8.20 (d, J = 7.5 Hz, 2H), 7.50 – 7.37 (m, 16H), 7.16 (d, J = 6.5 Hz, 6H), 5.53 (s, 4H). MALDI-TOF-MS (m/z): [M + H]+ calcd. for C_52_H_32_F_2_N_4_S_3_, 847.0; found, 846.7. Anal. calcd. for C_52_H_32_F_2_N_4_S_3_: C, 73.74; H, 3.81; N, 6.61; S, 11.35; found: C, 70.53; H, 3.54; N, 6.24; S, 11.85.

*Synthesis of A5D3:* 4,7-Bis(5-bromothiophen-2-yl)-5,6-difluorobenzo[c][1,2,5]thiadiazole (80.0 mg, 0.2 mmol), 9-hexyl-2-(4,4,5,5-tetramethyl-1,3,2-dioxaborolan-2-yl)-9H-carbazole (153.0 mg, 0.4 mmol), tetrakis(-triphenylphosphine) palladium (0) (18.5 mg, 16.0 µmol), K_2_CO_3_ (0.6 mL, 2.5 M) and aliquat 336 (1-2 drops) were taken into a 10-mL pyrex vessel. Mixture was dissolved by adding toluene (3 mL) into the vessel and the cap of the vessel was sealed in the glove box under inert atmosphere. The reaction mixture stirred and purged with N2 at room temperature for 15 minutes. Then, the mixture was irradiated at 110 °C for 1 h using a microwave reactor by moderating the initial power at 150W. After the reaction was completed, the organic phase was separated from the aqueous phase via decantation and the organic phase concentrated in a sample concentrator. The crude product was purified with a column chromatography on silica gel filled in 20 mL syringe cartages using the vacuum manifold in a gradient solvent system of hexane/chloroform. The structure and composition were supported by characterization results. (59.6 mg, 44.3 %) ^1^H NMR (400 MHz, CDCl_3_, δ): 88.34 (d, J = 3.9 Hz, 2H), 8.13 – 8.08 (m, 4H), 7.71 (s, 2H), 7.62 (dd, J = 8.0, 1.3 Hz, 2H), 7.57 (d, J = 3.9 Hz, 2H), 7.48 (t, J = 7.6 Hz, 2H), 7.42 (d, J = 8.1 Hz, 2H), 7.25 – 7.20 (m, 2H), 4.37 (t, J = 7.2 Hz, 4H), 1.93 (p, J = 7.4 Hz, 4H), 1.44 – 1.29 (m, 12H), 0.91 – 0.87 (m, 6H). MALDI-TOF-MS (m/z): [M + H]+ calcd. for C_50_H_44_F_2_N_4_S_3_, 835.1; found, 834.3. Anal. calcd. for C_50_H_44_F_2_N_4_S_3_: C, 71.91; H, 5.31; N, 6.71; S, 11.52; found: C, 68.13; H, 4.67; N, 5.91; S, 11.07.

*Synthesis of A5D4:* 4,7-Bis(5-bromothiophen-2-yl)-5,6-difluorobenzo[c][1,2,5]thiadiazole (50.0 mg, 0.1 mmol), [4-[bis(4-methoxyphenyl)amino]phenyl]boronic acid (88.3 mg, 0.3 mmol), tetrakis(-triphenylphosphine) palladium (0) (11.7 mg, 10.1 µmol), K_2_CO_3_ (0.4 mL, 2.5 M) and aliquat 336 (1-2 drops) were placed in a 10-mL pyrex vessel. The mixture was dissolved by adding 3 mL of toluene, and the vessel cap was sealed under inert atmosphere within a glove box. The reaction mixture was stirred and purged with nitrogen at room temperature for 15 minutes. Afterwards, the mixture was subjected to irradiation at 110 °C for 1 hour in a microwave reactor, with the initial power set at 150 W. Once the reaction was finished, the organic phase was separated from the aqueous phase by decantation, and the organic layer was concentrated using a sample concentrator. The crude product was purified via column chromatography on silica gel packed in 20 mL syringe cartridges, utilizing a vacuum manifold and a gradient solvent system of hexane and chloroform. The final pure product was obtained as a red-purple solid. (90.6 mg, 95.1 %) ^1^H NMR (400 MHz, CHCl_3_, δ): 8.24 (d, J = 4.0 Hz, 2H), 7.52 (d, J = 7.9 Hz, 4H), 7.31 (s, 2H), 7.09 (s, 8H), 6.94 (s, 4H), 6.86 (d, J = 9.0 Hz, 8H), 3.82 (s, 12H). MALDI-TOF-MS (m/z): [M + H]+ calcd. for C_54_H_40_F_2_N_4_O_4_S_3_, 943.1; found, 942.9. Anal. calcd. for C_54_H_40_F_2_N_4_O_4_S_3_: C, 68.77; H, 4.28; N, 5.94; S, 10.2; found: C, 68.13; H, 4.28; N, 5.50; S, 9.70.

*Synthesis of A5D5:* 4,7-Bis(5-bromothiophen-2-yl)-5,6-difluorobenzo[c][1,2,5]thiadiazole (60.0 mg, 0.1 mmol), 4-pyridinylboronic acid (37.3 mg, 0.3 mmol), tetrakis(-triphenylphosphine) palladium (0) (14.0 mg, 12.1 µmol), K_2_CO_3_ (0.5 mL, 2.5 M) and aliquat 336 (1-2 drops) were taken into a 10-mL pyrex vessel. The mixture was dissolved by introducing 3 mL of toluene, and the vessel cap was sealed inside a glove box under an inert atmosphere. The reaction mixture was stirred and purged with nitrogen at room temperature for 15 minutes. Subsequently, the mixture was irradiated at 110 °C for 1 hour in a microwave reactor, with the initial power set at 150 W. Upon completion of the reaction, the organic phase was separated from the aqueous phase by decantation, and the organic layer was concentrated using a sample concentrator. The product was purified by recrystallization, achieved by dissolving it in chloroform and precipitating over cold hexane. Ultimately, the product was obtained as a yellow solid but couldn’t be purified further due to low solubility. (98 mg) ^1^H NMR (400 MHz, DMSO, δ): 8.75 – 8.48 (m, 4H), 7.93 – 7.46 (m, 8H). MALDI-TOF-MS (m/z): [M + H]^+^ calcd. for C_24_H_12_F_2_N_4_S_3_, 490.6; found, 491.4. Anal. calcd. for C_24_H_12_F_2_N_4_S_3_: C, 58.76; H, 2.47; N, 11.42; S, 19.61; found: C, 33.30; H, 1.57; N, 5.27; S, 10.04.

*Synthesis of A5D6:* 4,7-Bis(5-bromothiophen-2-yl)-5,6-difluorobenzo[c][1,2,5]thiadiazole (60.0 mg, 0.1 mmol), 2-tributylstannylthiazole (113.6 mg, 0.3 mmol) and tetrakis(-triphenylphosphine) palladium (0) (14.0 mg, 12.1 µmol) were mixed in a 10-mL Pyrex vessel. The blend was dissolved by adding 3 mL of toluene inside a glove box under inert conditions, and the vessel was sealed. The reaction mixture was then stirred and purged with nitrogen at room temperature for 15 minutes. Afterwards, the mixture was irradiated in a microwave reactor at 110 °C for 1 hour, with an initial power of 150 W. The crude product was purified by column chromatography on silica gel packed in 20 mL syringe cartridges, using a vacuum manifold and a gradient solvent system of hexane and chloroform. The final product was attained as a yellow-orange solid but couldn’t be purified further due to low solubility. (38.9 mg, 63.9 %) ^1^H NMR (400 MHz, CDCl_3_, δ): 8.30 (d, J = 4.1 Hz, 2H), 7.85 (d, J = 3.2 Hz, 2H), 7.66 (d, J = 4.0 Hz, 2H), 7.34 (d, J = 3.2 Hz, 2H). MALDI-TOF-MS (m/z): [M + H]^+^ calcd. For C_20_H_8_F_2_N_4_S_5_, 502.6; found, 501.9. Anal. calcd. for C_20_H_8_F_2_N_4_S_5_: C, 47.79; H, 1.60; N, 11.15; S, 31.89; found: C, 44.08; H, 1.58; N, 9.10; S, 27.10.

*Synthesis of A7D1:* 3,6-Bis(5-bromo-2-thienyl)-2,5-bis(2-hexyldecyl)-2,5-dihydro-pyrrolo[3,4-c]pyr- 1,4-dione (60.0 mg, 66.2 µmol), 9H-carbazole-9-(4-phenyl)boronic acid pinacol ester (61.1 mg, 0.2 mmol), tetrakis(triphenylphosphine)palladium(0) (7.6 mg, 6.6 µmol), K_2_CO_3_ (0.300, mL, 2.5 M), and aliquat 336 (1-2 drops) were placed in a 10-mL Pyrex vessel. The mixture was dissolved by adding 3 mL of toluene, and the vessel was sealed with a cap in an inert atmosphere within the glove box. The reaction mixture was stirred and purged with nitrogen for 15 minutes at room temperature. After that, the mixture was subjected to irradiation at 110 °C for 1 hour using a microwave reactor, with the initial power set to 150 W. Once the reaction was complete, the organic phase was separated from the aqueous layer by decantation, and the organic layer was concentrated using a sample concentrator. The pure product was isolated as a yellow solid following purification by column chromatography on silica gel packed in 20 mL syringe cartridges using a vacuum manifold with a gradient solvent system of hexane and chloroform. The final product was obtained as a purple-blue solid. (56.8 mg, 69.7 %) ^1^H NMR (400 MHz, CDCl_3_, δ): 9.01 (d, J = 4.0 Hz, 2H), 8.17 (d, J = 7.6 Hz, 4H), 7.92 (d, J = 8.6 Hz, 4H), 7.69 – 7.64 (m, 4H), 7.58 (d, J = 4.1 Hz, 2H), 7.52 – 7.42 (m, 8H), 7.32 (ddd, J = 8.0, 6.9, 1.3 Hz, 4H), 4.13 (d, J = 7.8 Hz, 4H), 2.04 (s, 2H), 1.38 (d, J = 7.0 Hz, 12H), 1.24 (d, J = 12.3 Hz, 34H), 0.88 – 0.80 (m, 12H). MALDI-TOF-MS (m/z): [M + H]+ calcd. for C_82_H_94_N_4_O_2_S_2_, 1231.8; found, 1231.6. Anal. calcd. for C_82_H_94_N_4_O_2_S_2_: C, 79.96; H, 7.69; N, 4.55; S, 5.21; found: C, 80.00; H, 7.93; N, 4.32; S, 5.05.

*Synthesis of A7D2:* 3,6-Bis(5-bromo-2-thienyl)-2,5-bis(2-hexyldecyl)-2,5-dihydro-pyrrolo[3,4-c]pyrrole-1,4- (60.0 mg, 66.2 µmol), 9-benzyl-9H-carbazole-3-boronic acid pinacol ester (63.4 mg, 0.2 mmol), tetrakis(-triphenylphosphine) palladium (0) (7.6 mg, 6.6 µmol), K_2_CO_3_ (0.3 mL, 2.5 M) and aliquat 336 (1-2 drops) were added to a 10-mL Pyrex vessel. The mixture was dissolved by introducing 3 mL of toluene, and the vessel cap was sealed in an inert atmosphere within a glove box. The reaction mixture was stirred and purged with nitrogen at room temperature for 15 minutes. Following this, the mixture was subjected to irradiation at 110 °C for 1 hour in a microwave reactor, with the initial power adjusted to 150 W. Once the reaction was complete, the organic phase was separated from the aqueous phase by decantation, and the organic layer was concentrated using a sample concentrator. The crude product was purified through column chromatography on silica gel packed in 20 mL syringe cartridges, utilizing a vacuum manifold with a hexane/chloroform gradient solvent system. The pure product was obtained as a green-blue solid. (36.5 mg, 43.8 %) ^1^H NMR (400 MHz, CDCl_3_, δ): 9.01 (d, J = 4.1 Hz, 2H), 8.63 (s, 1H), 8.42 (d, J = 1.5 Hz, 2H), 8.18 (dd, J = 13.4, 7.7 Hz, 4H), 7.89 (dd, J = 8.2, 1.0 Hz, 1H), 7.76 (dd, J = 8.6, 1.8 Hz, 2H), 7.51 (d, J = 4.1 Hz, 2H), 7.39 (d, J = 8.5 Hz, 4H), 7.36 (s, 2H), 7.14 (dd, J = 15.2, 6.0 Hz, 8H), 5.54 (d, J = 5.4 Hz, 4H), 4.13 (d, J = 7.6 Hz, 4H), 2.06 (s, 2H), 1.40 (s, 48H), 0.81 (d, J = 3.0 Hz, 12H). MALDI-TOF-MS (m/z): [M + H]^+^ calcd. for C_84_H_98_N_4_O_2_S_2_, 1259.9; found, 1258.7. Anal. calcd. for C_84_H_98_N_4_O_2_S_2_: C, 80.08; H, 7.84; N, 4.45; S, 5.09; found: C, 79.88; H, 7.78; N, 4.24; S, 5.19.

*Synthesis of A7D3:* 3,6-Bis(5-bromo-2-thienyl)-2,5-bis(2-hexyldecyl)-2,5-dihydro-pyrrolo[3,4-c]pyrrole-1,4- (60.0 mg, 0.1 mmol), 9-hexyl-2-(4,4,5,5-tetramethyl-1,3,2-dioxaborolan-2-yl)-9H-carbazole (62.4 mg, 0.2 mmol), tetrakis(-triphenylphosphine) palladium (0) (3.8 mg, 3.3 µmol), K_2_CO_3_ (0.3 mL, 2.5 M) and aliquat 336 (1-2 drops) added to a 10-mL Pyrex vessel. The mixture was dissolved by adding 3 mL of toluene, and the vessel cap was sealed under an inert atmosphere within a glove box. The reaction mixture was stirred and purged with nitrogen at room temperature for 15 minutes. The mixture was then irradiated at 110 °C for 1 hour in a microwave reactor, with the initial power adjusted to 150 W. Once the reaction was complete, the organic phase was separated from the aqueous phase through decantation, and the organic layer was concentrated using a sample concentrator. The crude product was purified using column chromatography on silica gel packed in 20 mL syringe cartridges with a vacuum manifold, employing a gradient solvent system of hexane and chloroform. The final pure product was acquired as a purple solid. (50.6 mg, 61.2 %) ^1^H NMR (400 MHz, CDCl_3_, δ): 9.02 (d, J = 4.1 Hz, 2H), 8.11 (d, J = 8.2 Hz, 4H), 7.67 (d, J = 1.5 Hz, 2H), 7.60 – 7.38 (m, 10H), 4.35 (t, J = 7.1 Hz, 4H), 4.14 (d, J = 7.6 Hz, 4H), 2.05 (s, 2H), 1.95 – 1.88 (m, 4H), 1.43 – 1.30 (m, 64H), 0.88 (t, J = 7.0 Hz, 18H). MALDI-TOF-MS (m/z): [M + H]+ calcd. for C_82_H_110_N_4_O_2_S_2_, 1247.9; found, 1246.8. Anal. calcd. for C_82_H_110_N_4_O_2_S_2_: C, 78.92; H, 8.89; N, 4.49; S, 5.14; found: C, 79.32; H, 8.79; N, 4.27; S, 5.27.

*Synthesis of A7D4:* 3,6-Bis(5-bromo-2-thienyl)-2,5-bis(2-hexyldecyl)-2,5-dihydro-pyrrolo[3,4-c]pyrrole-1,4- (60.0 mg, 66.2 µmol), [4-[bis(4-methoxyphenyl)amino]phenyl]boronic acid (57.7 mg, 0.2 mmol), tetrakis(-triphenylphosphine) palladium (0) (7.6 mg, 6.6 µmol), K_2_CO_3_ (0.3 mL, 2.0 M) and aliquat 336 (1-2 drops) were taken into a 10-mL pyrex vessel. The mixture was dissolved by 3 mL of toluene, and the vessel was sealed under an inert atmosphere. The reaction mixture was stirred and purged with nitrogen at room temperature for 15 minutes. Subsequently, the mixture was irradiated at 110 °C for 1 hour in a microwave reactor, maintaining the initial power at 150 W. Upon completion of the reaction, the organic phase was separated from the aqueous phase by decantation, and the organic layer was concentrated using a sample concentrator. The crude product was purified by column chromatography on silica gel in 20 mL syringe cartridges with a vacuum manifold, employing a gradient solvent system of hexane and chloroform. The final pure product was obtained as a dark blue solid. (87.2 mg, 97.1 %) ^1^H NMR (400 MHz, CDCl_3_, δ): 7.45 (s, 4H), 7.09 (d, J = 8.1 Hz, 10H), 6.87 (t, J = 10.2 Hz, 14H), 4.05 (d, J = 6.6 Hz, 4H), 3.81 (s, 12H), 1.99 (s, 2H), 1.34 – 1.19 (m, 46H), 0.85 – 0.79 (m, 12H). MALDI-TOF-MS (m/z): [M + H]+ calcd. for C_86_H_106_N_4_O_6_S_2_, 1355.9; found, 1356.8. Anal. calcd. for C_86_H_106_N_4_O_6_S_2_: C, 76.18; H, 7.88; N, 4.13; S, 4.73; found: C, 76.29; H, 8.09; N, 4.01; S, 4.57.

*Synthesis of A7D5:* 3,6-Bis(5-bromo-2-thienyl)-2,5-bis(2-hexyldecyl)-2,5-dihydro-pyrrolo[3,4-c]pyrrole-1,4- (80.0 mg, 88.2 µmol), 4-pyridinylboronic acid (27.1 mg, 0.2 mmol), tetrakis(-triphenylphosphine) palladium (0) (10.2 mg, 8.8 µmol), K_2_CO_3_ (0.4 mL, 2.0 M) and aliquat 336 (1-2 drops) were added to a 10-mL pyrex vessel. The mixture was dissolved by incorporating 3 mL of toluene, after which the vessel cap was sealed in a glove box under an inert atmosphere. The reaction mixture was stirred and purged with nitrogen for 15 minutes at room temperature. Following this, the mixture was irradiated in a microwave reactor at 110 °C for 1 hour, regulating the initial power at 150 W. After the reaction was complete, the organic layer was separated from the aqueous layer through decantation, and the organic phase was concentrated using a sample concentrator. The crude product was then purified by column chromatography on silica gel packed in 20 mL syringe cartridges with a vacuum manifold, utilizing a gradient solvent system of hexane and chloroform. The final purified product was obtained as a blue solid. (162.4 mg, 203.8 %) ^1^H NMR (400 MHz, CDCl_3_, δ): 8.92 (d, J = 4.3 Hz, 1H), 8.67 – 8.65 (m, 2H), 7.64 (dd, J = 2.8, 1.3 Hz, 2H), 7.55 – 7.52 (m, 3H), 7.49 – 7.43 (m, 4H), 3.44 (s, 4H), 1.66 (s, 2H), 1.26 (s, 46H), 0.88 (s, 12H). MALDI-TOF-MS (m/z): [M + H]^+^ calcd. for C_56_H_78_N_4_O_2_S_2_, 903.4; found, 904.2. Anal. calcd. for C_56_H_78_N_4_O_2_S_2_: C, 74.45; H, 8.70; N, 6.20; S, 7.10; found: C, 74.21; H, 8.50; N, 5.08; S, 6.90.

*Synthesis of A7D6:* 3,6-Bis(5-bromo-2-thienyl)-2,5-bis(2-hexyldecyl)-2,5-dihydro-pyrrolo[3,4-c]pyrrole-1,4- (85.0 mg, 0.1 mmol), 2-tributylstannylthiazole (87.7 mg, 0.2 mmol) and tetrakis(-triphenylphosphine) palladium (0) (10.8 mg, 9.4 µmol) were taken into a 10-mL pyrex vessel. The blend was dissolved by adding 3 mL of toluene inside a glove box under an inert atmosphere, after which the vessel cap was sealed. The reaction mixture was then stirred and purged with nitrogen at room temperature for 15 minutes. Following this, the mixture was irradiated for 1 hour at 110 °C in a microwave reactor, starting with an initial power of 150 W. The crude product was subsequently purified by column chromatography on silica gel, using 20 mL syringe cartridges and a vacuum manifold with a gradient solvent system consisting of hexane and chloroform. The purified product was obtained as a blue solid, its structure and composition were confirmed by characterization data. (55.0 mg, 63.9 %) ^1^H NMR (400 MHz, CDCl_3_, δ): 8.99 (d, J = 4.2 Hz, 1H), 8.10 (s, 4H), 7.85 (d, J = 3.2 Hz, 1H), 7.63 (d, J = 4.2 Hz, 1H), 7.38 (d, J = 3.2 Hz, 1H), 4.54 – 4.02 (m, 4H), 2.00 – 1.91 (m, 2H), 1.42 – 1.11 (m, 48H), 0.85 (dd, J = 14.8, 8.1 Hz, 12H). MALDI-TOF-MS (m/z): [M + H]^+^ calcd. For C_52_H_74_N_4_O_2_S_4_, 915.4; found, 914.5. Anal. calcd. for C_52_H_74_N_4_O_2_S_4_: C, 68.23; H, 8.15; N, 6.12; S, 14.01; found: C, 70.61; H, 9.49; N, 3.64; S, 9.09.


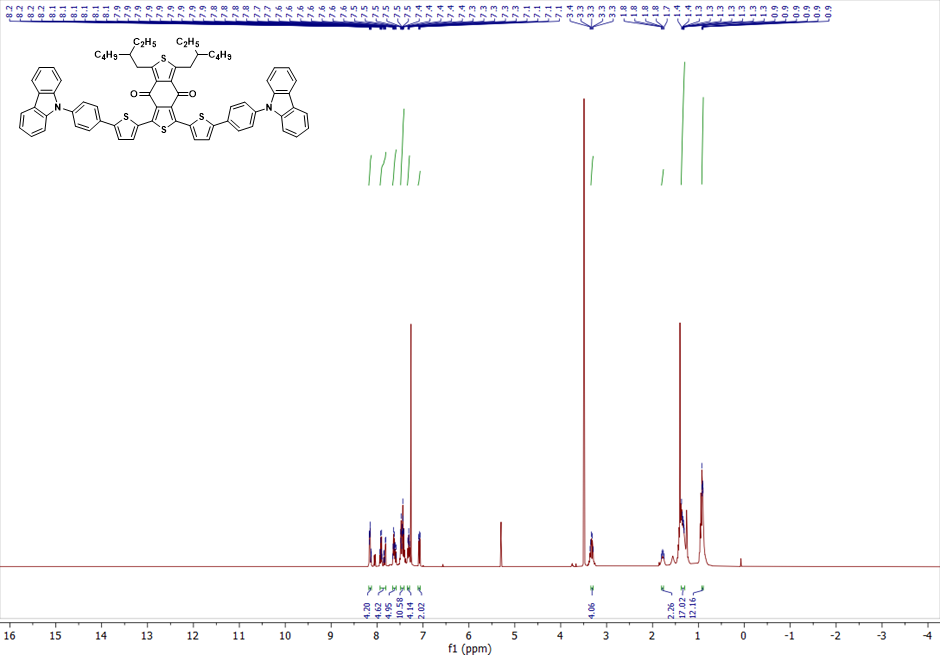


Figure S36. 1H-NMR spectrum of small molecule A2D1 in CDCl_3_ measured at 400 MHz and 25 °C.


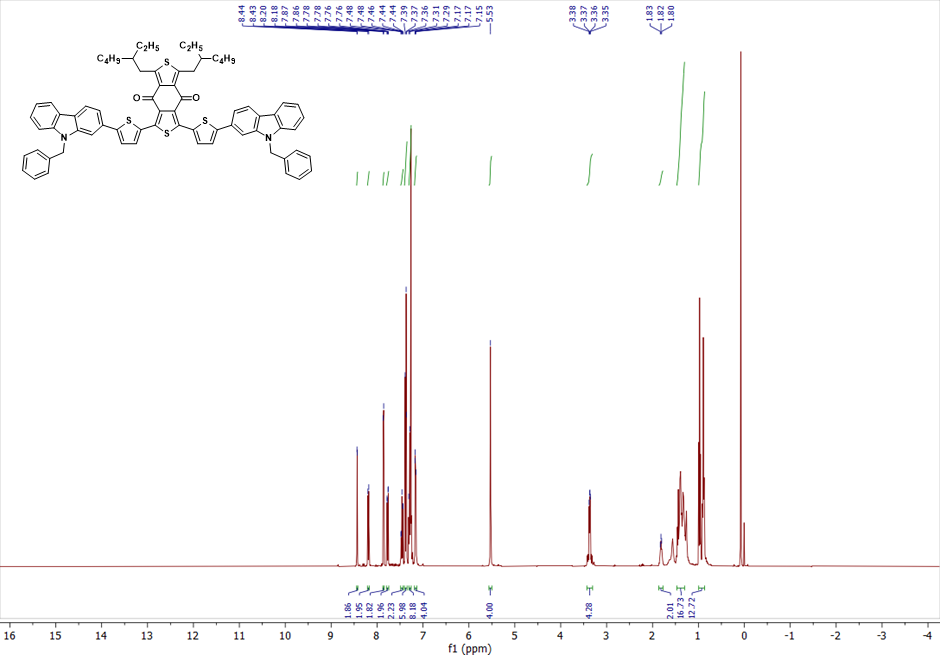


Figure S37. 1H-NMR spectrum of small molecule A2D2 in CDCl_3_ measured at 400 MHz and 25 °C.


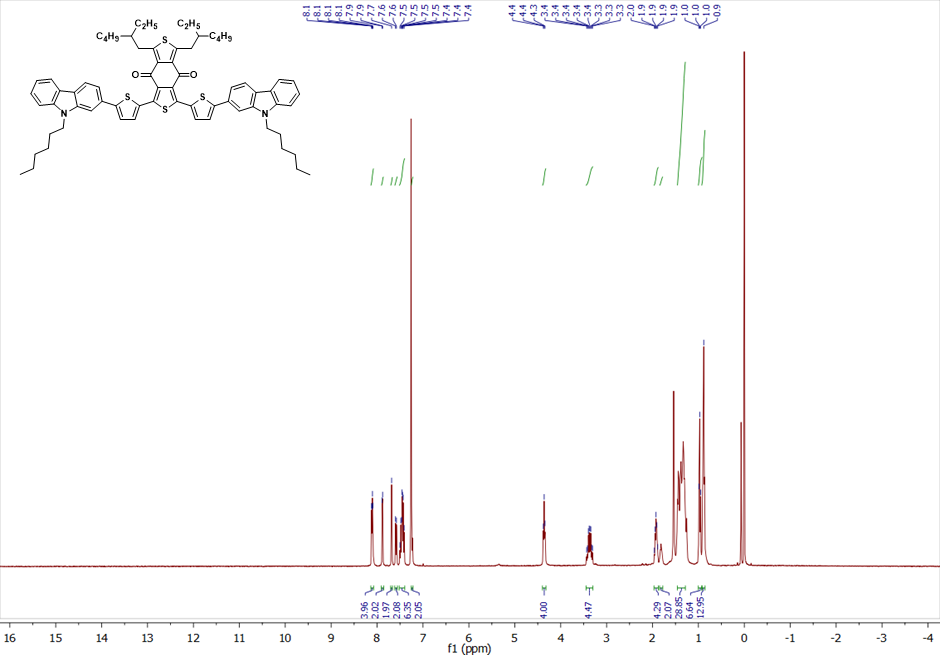


Figure S38. 1H-NMR spectrum of small molecule A2D3 in CDCl_3_ measured at 400 MHz and 25 °C.


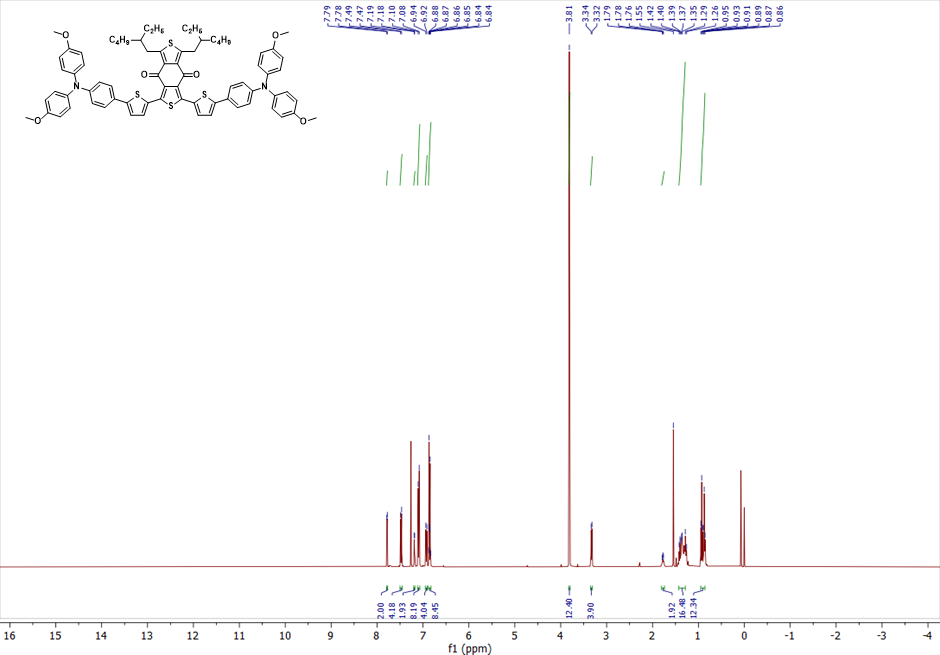


Figure S39. 1H-NMR spectrum of small molecule A2D4 in CDCl_3_ measured at 400 MHz and 25 °C.


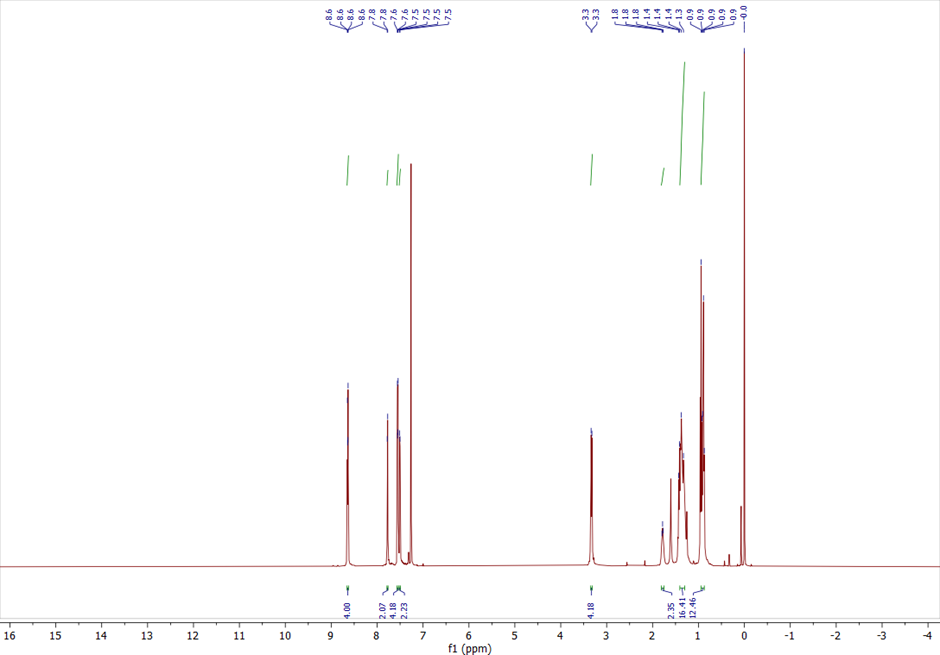


Figure S40. 1H-NMR spectrum of small molecule A2D5 in CDCl_3_ measured at 400 MHz and 25 °C.


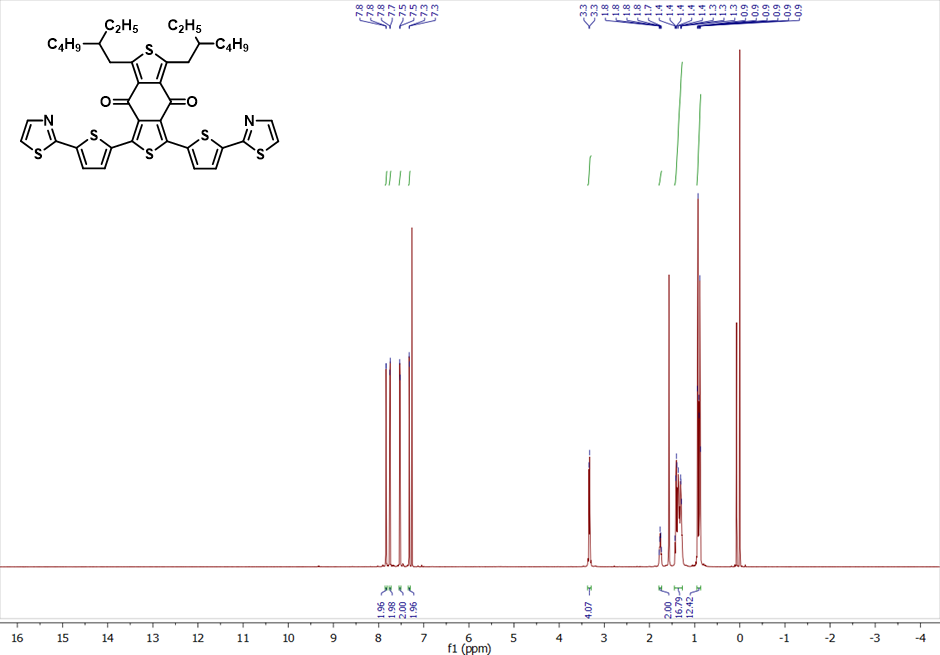


Figure S41. 1H-NMR spectrum of small molecule A2D6 in CDCl_3_ measured at 400 MHz and 25 °C.


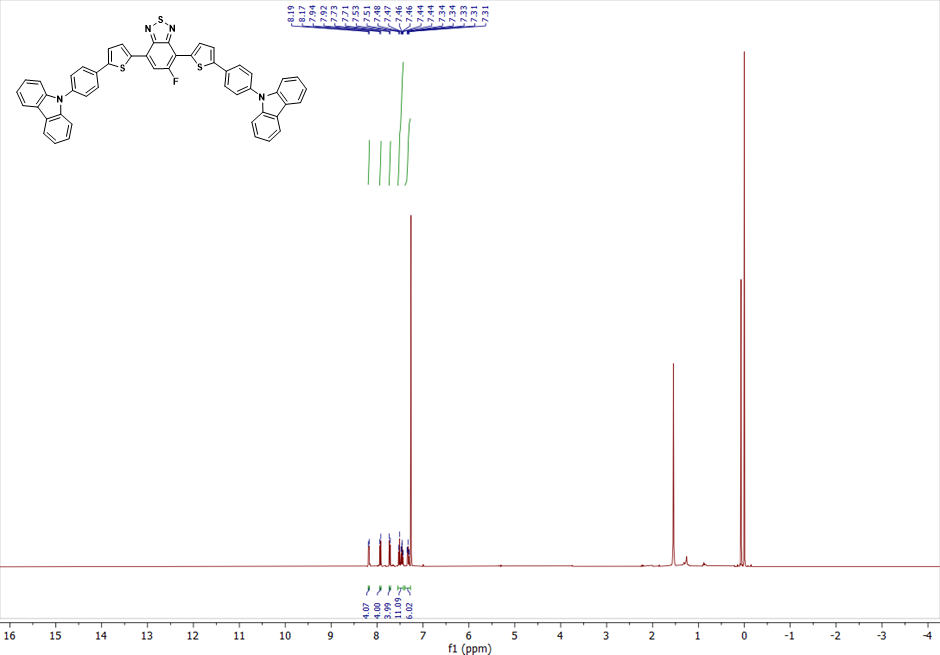


Figure S42. 1H-NMR spectrum of small molecule A4D1 in CDCl_3_ measured at 400 MHz and 25 °C.


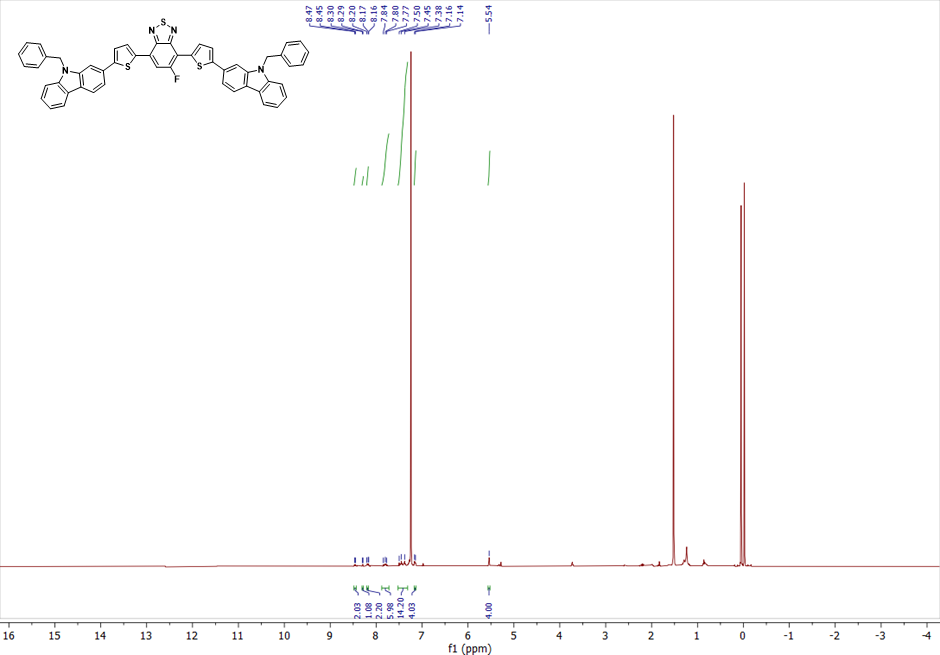


Figure S43. 1H-NMR spectrum of small molecule A4D2 in CDCl_3_ measured at 400 MHz and 25 °C.

*
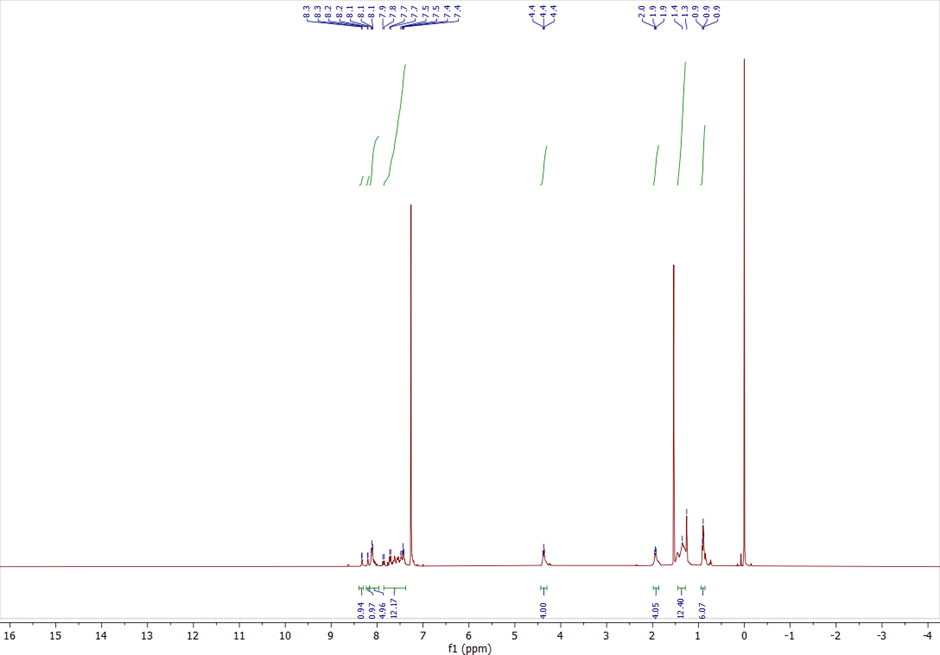
*

Figure S44. 1H-NMR spectrum of small molecule A4D3 in CDCl_3_ measured at 400 MHz and 25 °C.


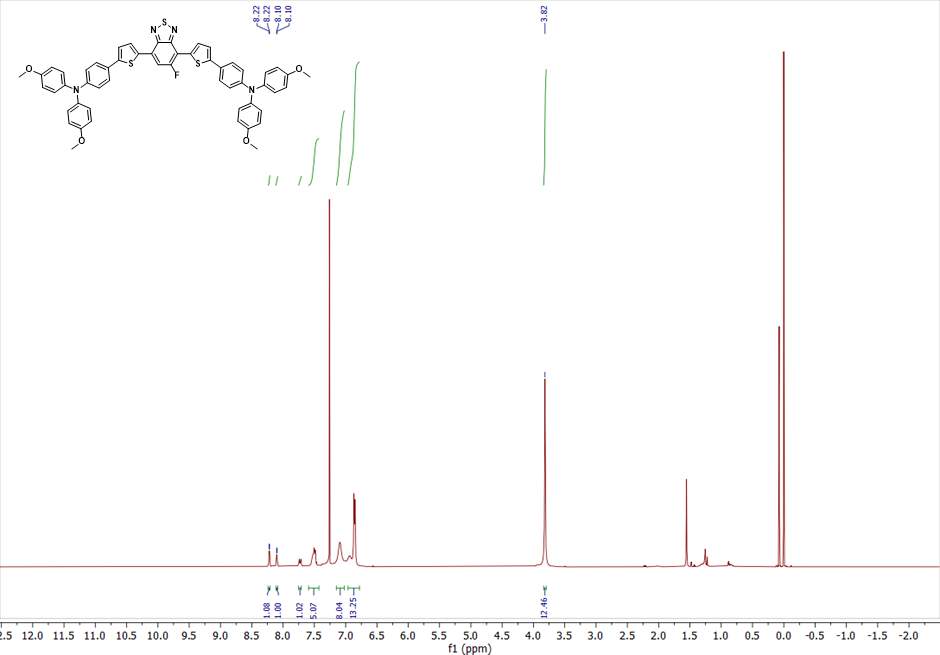


Figure S45. 1H-NMR spectrum of small molecule A4D4 in CDCl_3_ measured at 400 MHz and 25 °C.


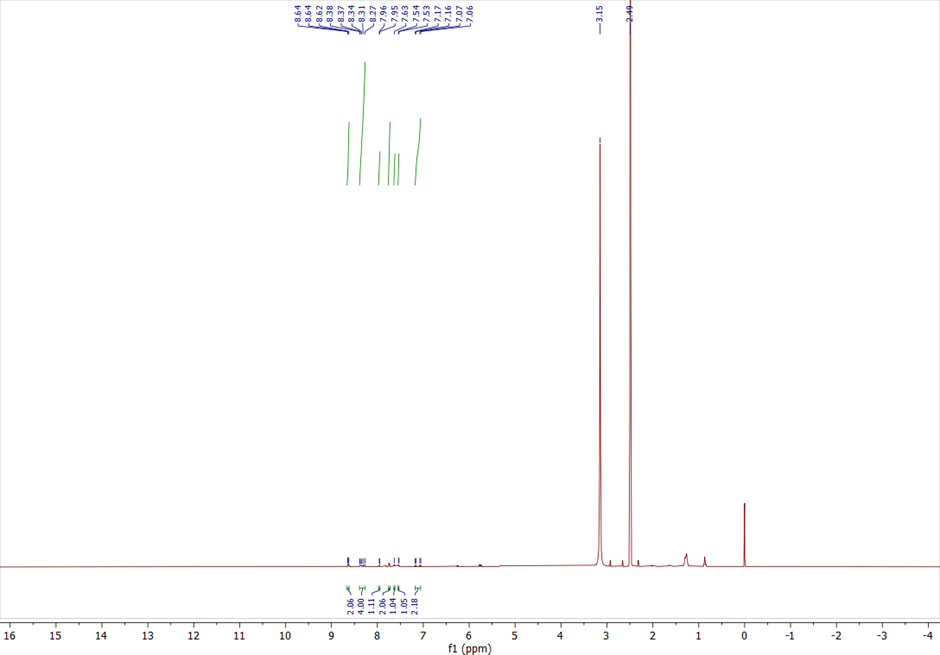


Figure S46. 1H-NMR spectrum of small molecule A4D5 in d-DMSO measured at 400 MHz and 25 °C.


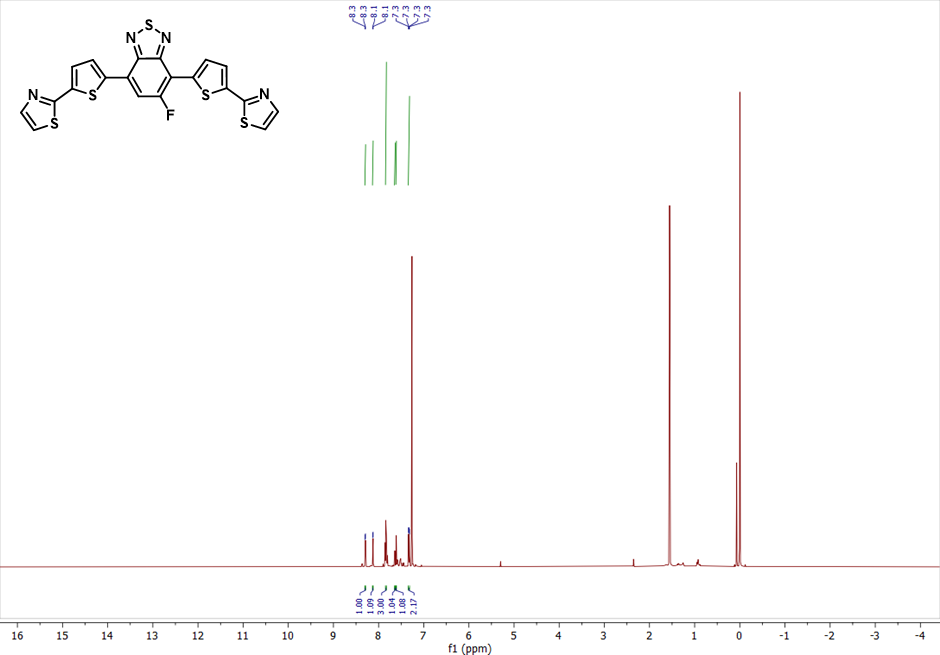


Figure S47. 1H-NMR spectrum of small molecule A4D6 in CDCl_3_ measured at 400 MHz and 25 °C.


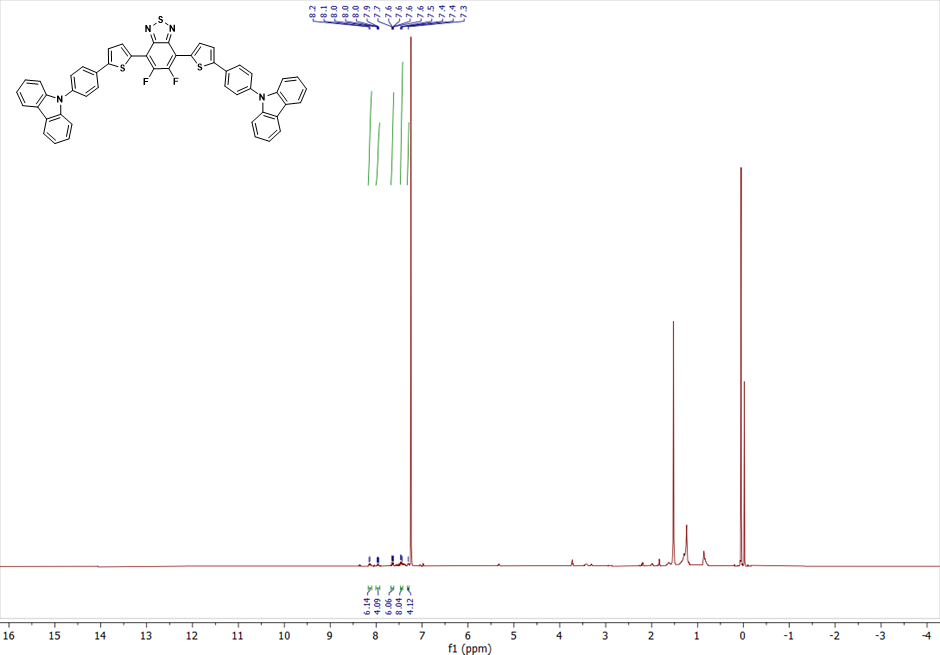


Figure S48. 1H-NMR spectrum of small molecule A5D1 in CDCl_3_ measured at 400 MHz and 25 °C.


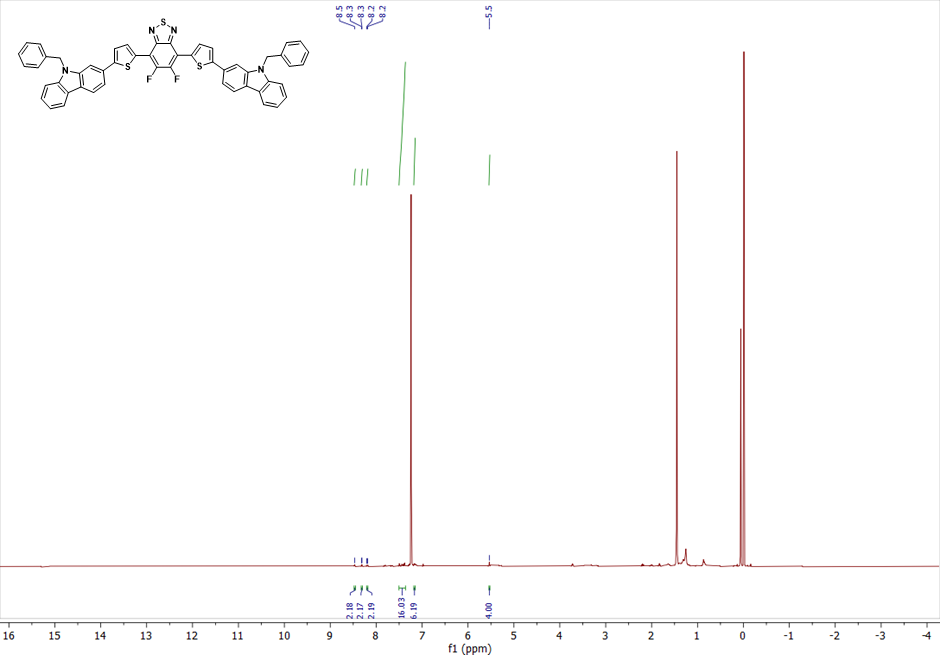


Figure S49. 1H-NMR spectrum of small molecule A5D2 in CDCl_3_ measured at 400 MHz and 25 °C.


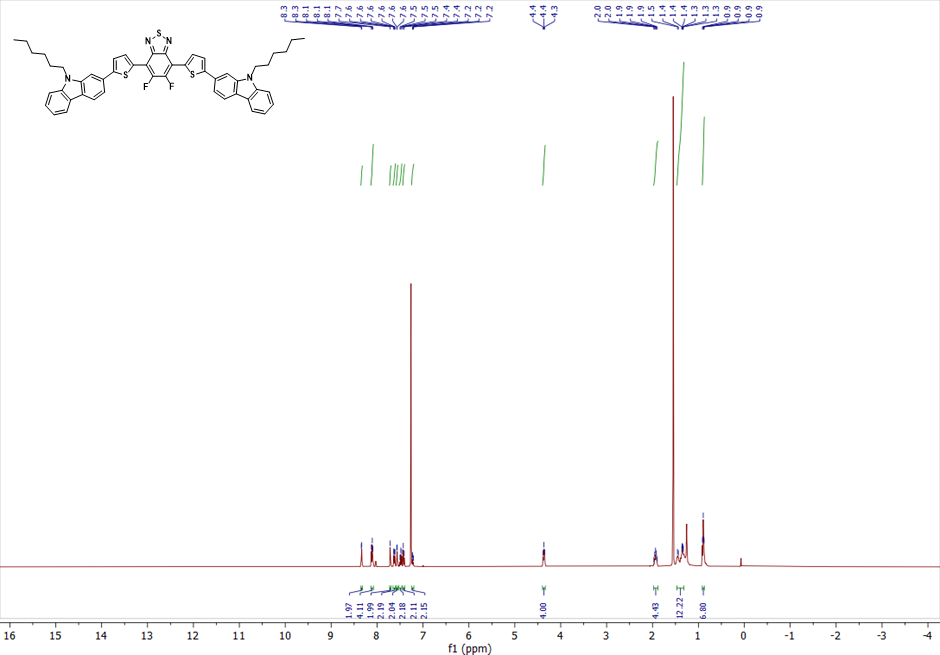


Figure S50. 1H-NMR spectrum of small molecule A5D3 in CDCl_3_ measured at 400 MHz and 25 °C.


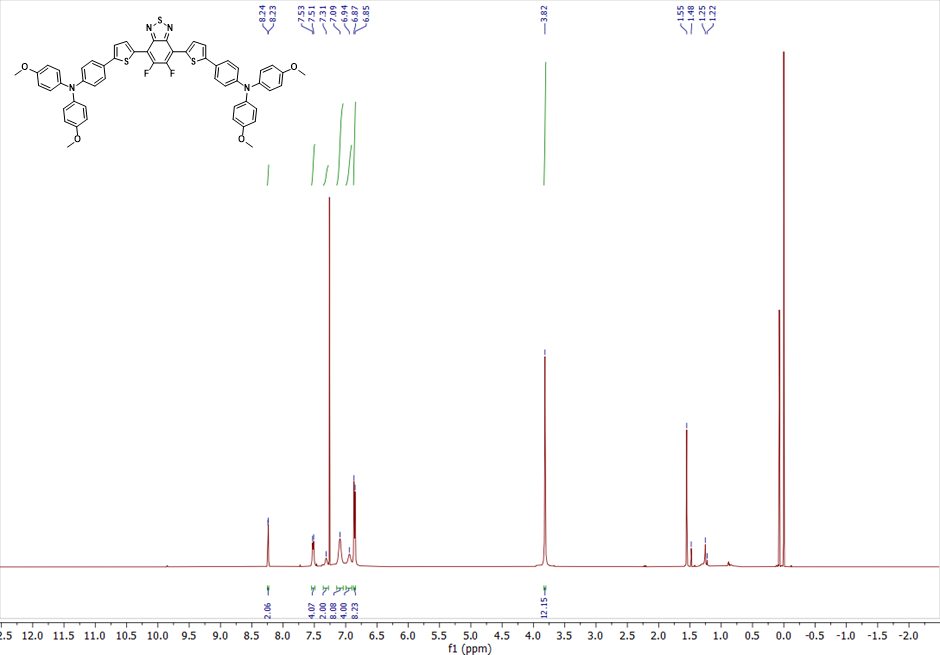


Figure S51. 1H-NMR spectrum of small molecule A5D4 in CDCl_3_ measured at 400 MHz and 25 °C.


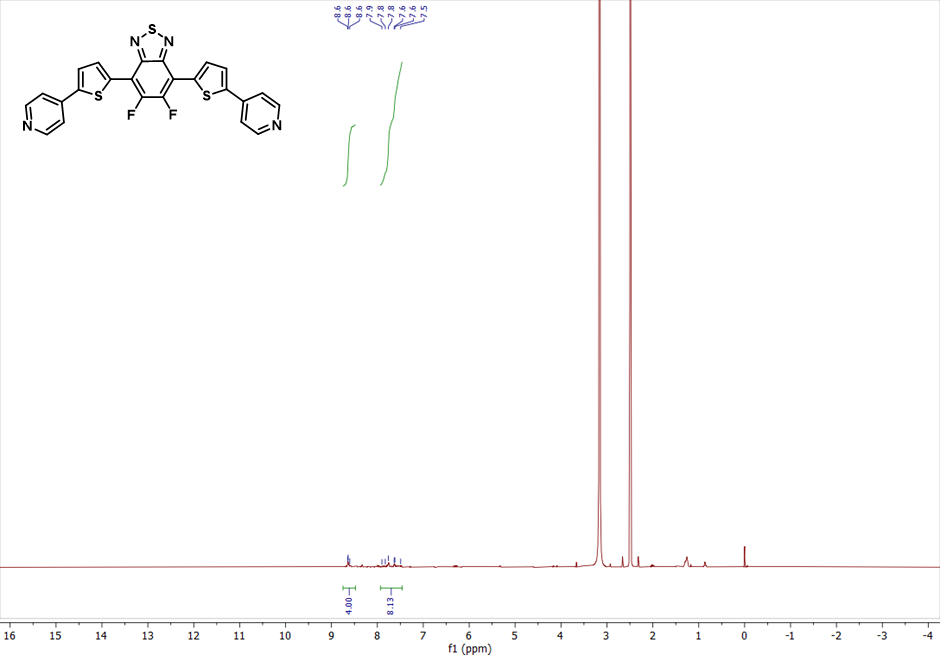


Figure S52. 1H-NMR spectrum of small molecule A5D5 in d-DMSO measured at 400 MHz and 25 °C.


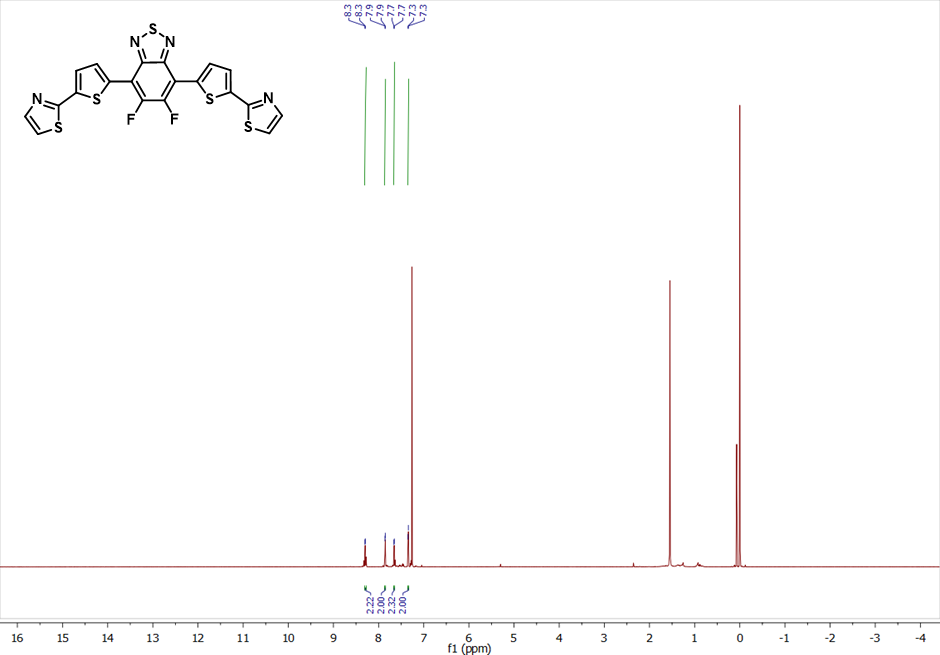


Figure S53. 1H-NMR spectrum of small molecule A5D6 in CDCl_3_ measured at 400 MHz and 25 °C.


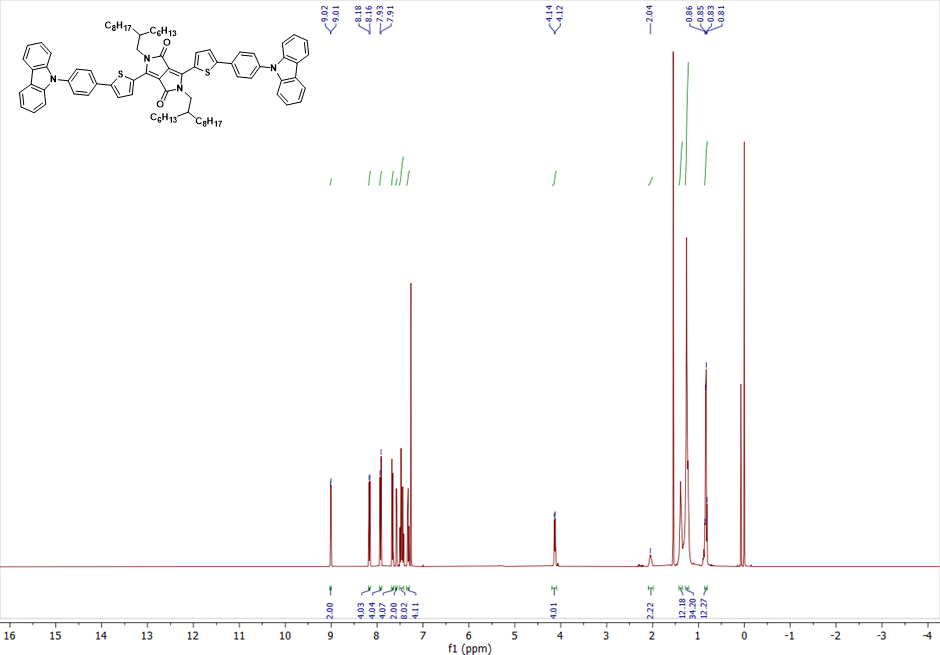


Figure S54. 1H-NMR spectrum of small molecule A7D1 in CDCl_3_ measured at 400 MHz and 25 °C.


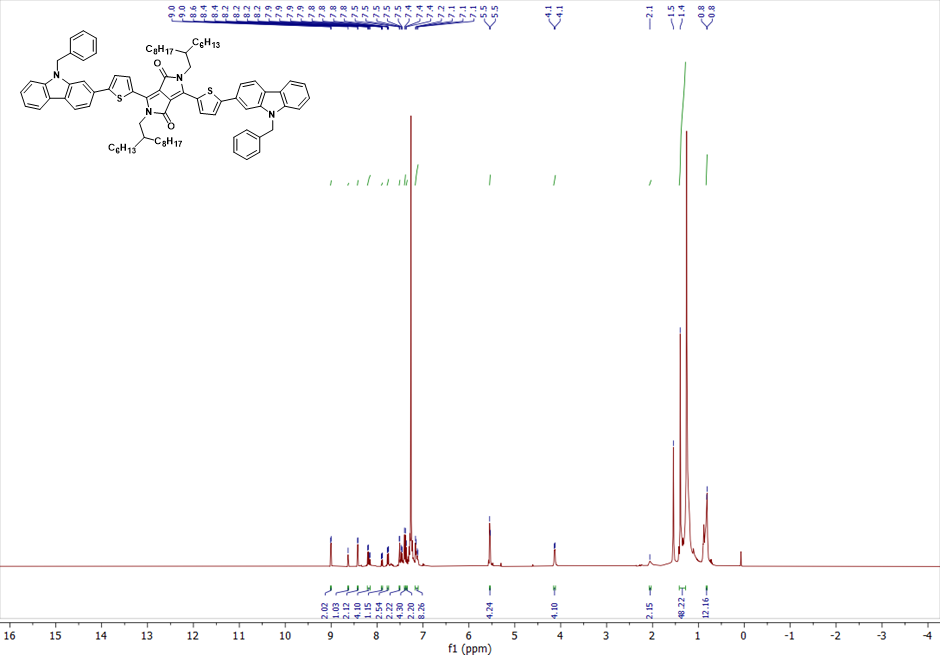


Figure S55. 1H-NMR spectrum of small molecule A7D2 in CDCl_3_ measured at 400 MHz and 25 °C.


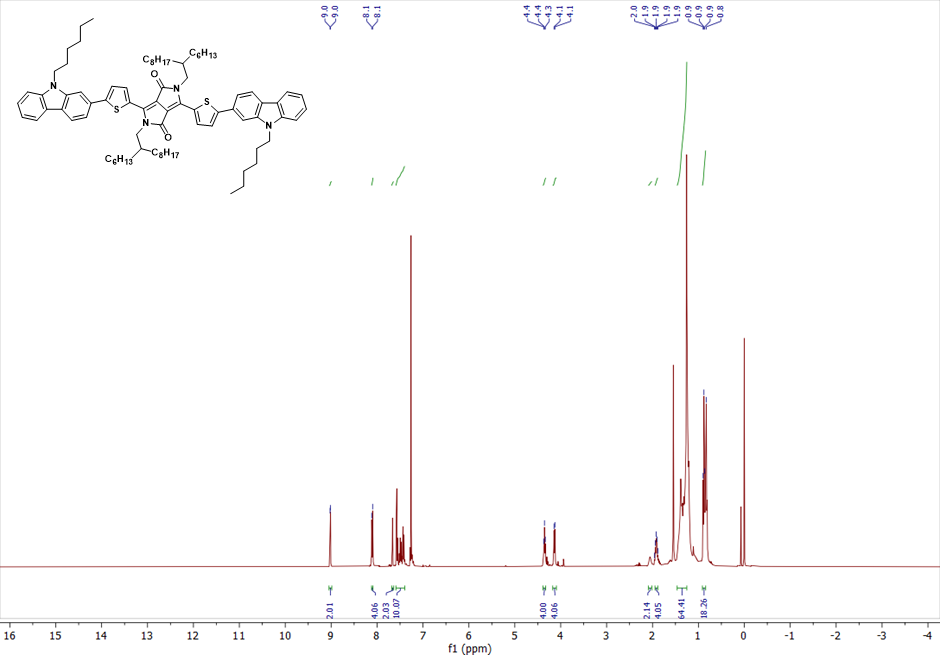


Figure S56. 1H-NMR spectrum of small molecule A7D3 in CDCl_3_ measured at 400 MHz and 25 °C.


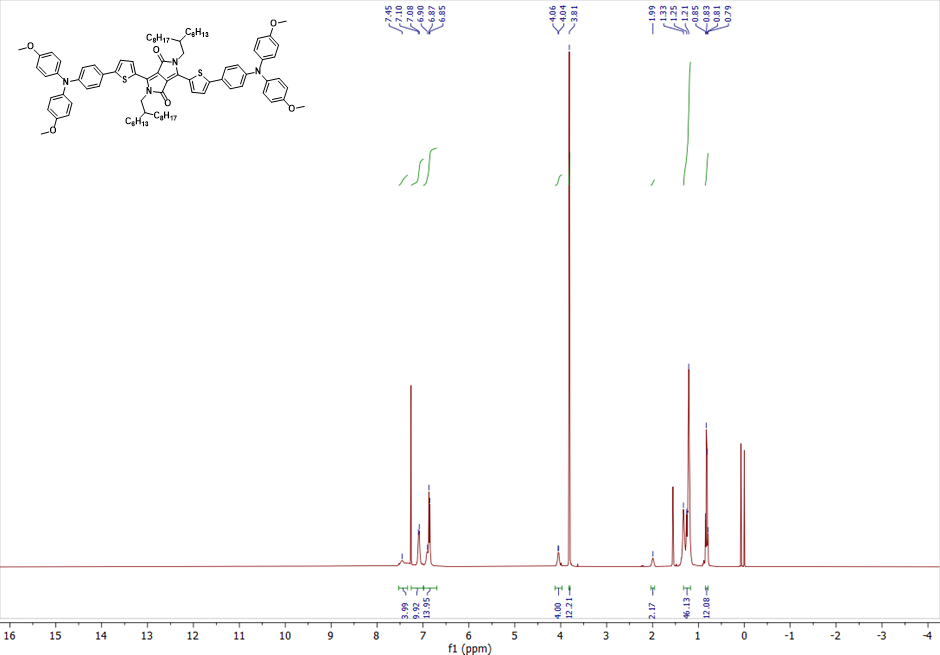


Figure S57. 1H-NMR spectrum of small molecule A7D4 in CDCl_3_ measured at 400 MHz and 25 °C.


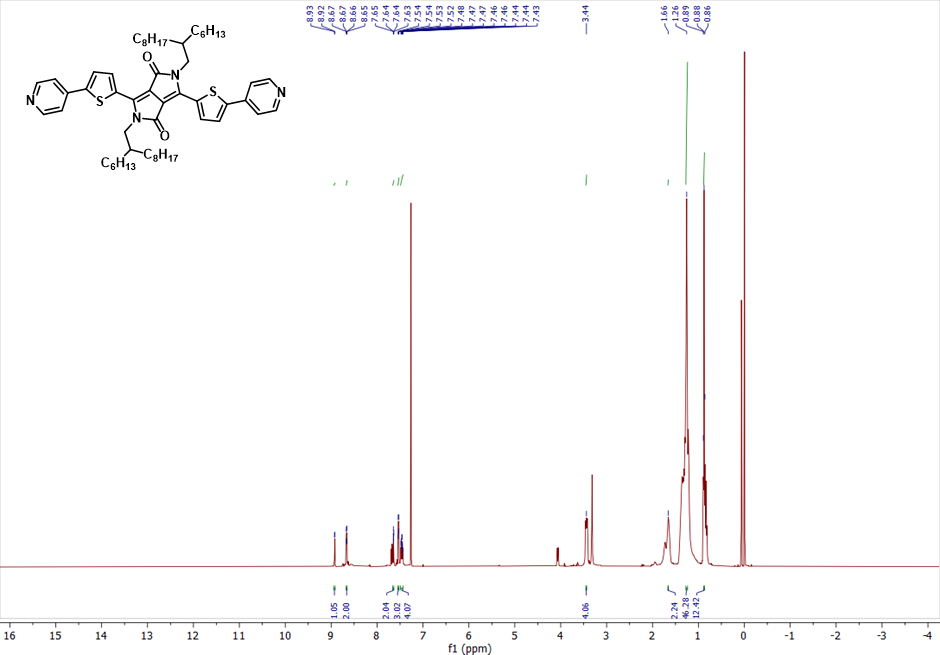


Figure S58. 1H-NMR spectrum of small molecule A7D5 in CDCl_3_ measured at 400 MHz and 25 °C.


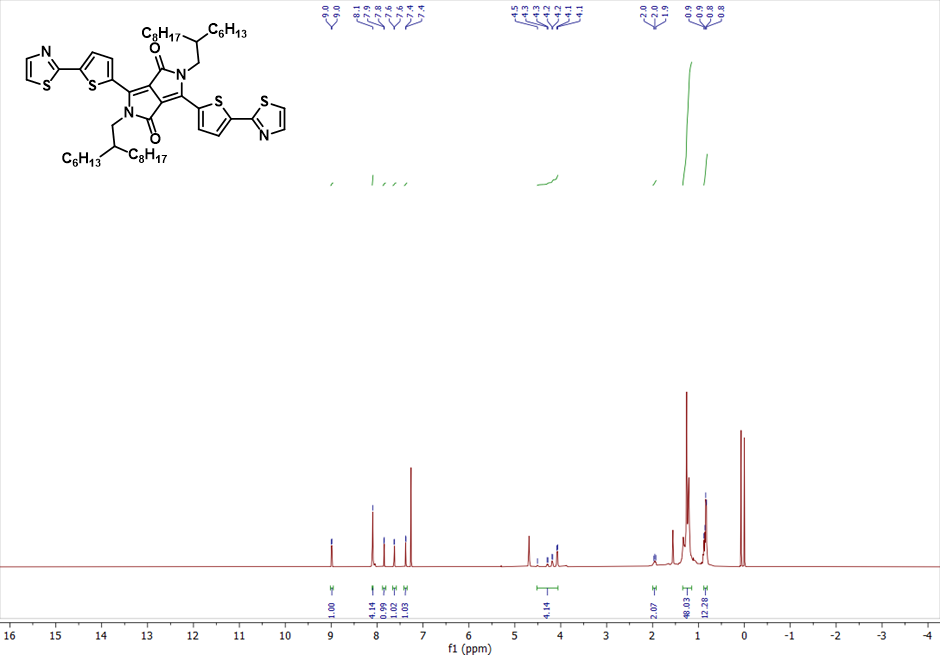


Figure S59. 1H-NMR spectrum of small molecule A7D6 in CDCl_3_ measured at 400 MHz and 25 °C.

Figure S60. HR-MALDI-TOF mass spectrum of A2D1.

Figure S61. HR-MALDI-TOF mass spectrum of A2D2.

Figure S62. HR-MALDI-TOF mass spectrum of A2D3.

Figure S63. HR-MALDI-TOF mass spectrum of A2D4.

Figure S64. HR-MALDI-TOF mass spectrum of A2D5.

Figure S65. HR-MALDI-TOF mass spectrum of A2D6.

Figure S67. HR-MALDI-TOF mass spectrum of A4D1.

Figure S68. HR-MALDI-TOF mass spectrum of A4D2.

Figure S69. HR-MALDI-TOF mass spectrum of A4D3.

Figure S70. HR-MALDI-TOF mass spectrum of A4D4.

Figure S71. HR-MALDI-TOF mass spectrum of A4D5.

Figure S72. HR-MALDI-TOF mass spectrum of A4D6.

Figure S573. HR-MALDI-TOF mass spectrum of A5D1.

Figure S74. HR-MALDI-TOF mass spectrum of A5D2.

Figure S75. HR-MALDI-TOF mass spectrum of A5D3.

Figure S76. HR-MALDI-TOF mass spectrum of A5D4.

Figure S77. HR-MALDI-TOF mass spectrum of A5D5.

Figure S78. HR-MALDI-TOF mass spectrum of A5D6.

Figure S79. HR-MALDI-TOF mass spectrum of A7D1.

Figure S80. HR-MALDI-TOF mass spectrum of A7D2.

Figure S81. HR-MALDI-TOF mass spectrum of A7D3.

Figure S82. HR-MALDI-TOF mass spectrum of A7D4.

Figure S83. HR-MALDI-TOF mass spectrum of A7D5.

Figure S84. HR-MALDI-TOF mass spectrum of A7D6.
